# Complete Genome Sequence and Comparative Metabolic Profiling of the Prototypical Enteroaggregative *Escherichia coli* Strain 042

**DOI:** 10.1371/journal.pone.0008801

**Published:** 2010-01-20

**Authors:** Roy R. Chaudhuri, Mohammed Sebaihia, Jon L. Hobman, Mark A. Webber, Denisse L. Leyton, Martin D. Goldberg, Adam F. Cunningham, Anthony Scott-Tucker, Paul R. Ferguson, Christopher M. Thomas, Gad Frankel, Christoph M. Tang, Edward G. Dudley, Ian S. Roberts, David A. Rasko, Mark J. Pallen, Julian Parkhill, James P. Nataro, Nicholas R. Thomson, Ian R. Henderson

**Affiliations:** 1 Department of Veterinary Medicine, University of Cambridge, Cambridge, United Kingdom; 2 Pathogen Genomics, The Wellcome Trust Sanger Institute, Cambridge, United Kingdom; 3 School of Biosciences, The University of Nottingham-Sutton Bonington Campus, Sutton Bonington, United Kingdom; 4 School of Immunity and Infection, University of Birmingham, Birmingham, United Kingdom; 5 School of Life and Environmental Sciences, University of Birmingham, Birmingham, United Kingdom; 6 Centre for Molecular Microbiology and Infection, Imperial College London, London, United Kingdom; 7 College of Agricultural Sciences, Pennsylvania State University, University Park, Pennsylvania, United States of America; 8 Faculty of Life Sciences, University of Manchester, Manchester, United Kingdom; 9 Institute for Genome Sciences, University of Maryland School of Medicine, Baltimore, Maryland, United States of America; 10 Center for Vaccine Development, University of Maryland School of Medicine, Baltimore, Maryland, United States of America; University of Hyderabad, India

## Abstract

**Background:**

*Escherichia coli* can experience a multifaceted life, in some cases acting as a commensal while in other cases causing intestinal and/or extraintestinal disease. Several studies suggest enteroaggregative *E. coli* are the predominant cause of *E. coli*-mediated diarrhea in the developed world and are second only to *Campylobacter* sp. as a cause of bacterial-mediated diarrhea. Furthermore, enteroaggregative *E. coli* are a predominant cause of persistent diarrhea in the developing world where infection has been associated with malnourishment and growth retardation.

**Methods:**

In this study we determined the complete genomic sequence of *E. coli* 042, the prototypical member of the enteroaggregative *E. coli*, which has been shown to cause disease in volunteer studies. We performed genomic and phylogenetic comparisons with other *E. coli* strains revealing previously uncharacterised virulence factors including a variety of secreted proteins and a capsular polysaccharide biosynthetic locus. In addition, by using Biolog™ Phenotype Microarrays we have provided a full metabolic profiling of *E. coli* 042 and the non-pathogenic lab strain *E. coli* K-12. We have highlighted the genetic basis for many of the metabolic differences between *E. coli* 042 and *E. coli* K-12.

**Conclusion:**

This study provides a genetic context for the vast amount of experimental and epidemiological data published thus far and provides a template for future diagnostic and intervention strategies.

## Introduction

The predominant facultative anaerobe resident in the human colon is the Gram-negative motile bacillus *Escherichia coli* [1]. *E. coli* colonises the infant gut within hours of birth. However, *E. coli* has a dichotomous existence; while the majority of *E. coli* strains exist within the mammalian intestinal tract as harmless commensals, paradoxically several evolutionary lineages have deviated from this harmless lifestyle to become pathogens. Current dogma suggests that such latter strains of *E. coli* have acquired additional genetic elements, encoding specific virulence factors, which enable the organism to cause disease when infecting an otherwise healthy individual. The resulting clinical syndromes include extraintestinal infections, such as urinary tract infections, septicaemia and meningitis, and intestinal infections mediating diarrhea. Those strains causing intestinal infections can be divided into six separate and major categories or pathotypes viz. enteroaggregative *E. coli* (EAEC), enteroinvasive (EIEC), enteropathogenic *E. coli* (EPEC), enterotoxigenic *E. coli* (ETEC), enterohaemorrhagic *E. coli* (EHEC) and diffuse adhering *E. coli* (DAEC) [2], [3]. The pathotype to which a particular strain belongs is defined by the clinical manifestation of disease, the repertoire of virulence factors, epidemiology and phylogenetic profiles [4].

EPEC were recognised as pathogens almost half a century ago [2]. However, it was not until much later that EPEC, EAEC and DAEC were distinguished from each other on the basis of their patterns of adherence to HEp-2 cells. Unlike the localised “microcolony-forming” pattern of adherence associated with EPEC, or the diffuse adherence pattern associated with DAEC, EAEC display a characteristic aggregative or “stacked-brick” pattern of adherence [5]. Based on these different adherence profiles Nataro *et al* demonstrated a significant association of EAEC with diarrhea in a case control study of children in Chile [5]. Immediately following the discovery of EAEC as a category of pathogenic *E. coli*, several epidemiological reports cast doubt on the pathogenic nature of EAEC [6]. However, the ability of this pathotype to mediate diarrhea was left in no doubt when a volunteer study demonstrated that EAEC strain 042 elicited diarrhea in the majority of volunteers [7]. Since these ground-breaking observations many studies have demonstrated the association of EAEC and diarrhea in both developing countries and industrialised nations. Thus, EAEC have been significantly associated with (i) endemic diarrhea in infants in developing and industrialised nations, particularly persistent diarrhea, (ii) persistent diarrhea in HIV-positive patients, (iii) traveller's diarrhea, (iv) food/water-borne outbreaks and (v) sporadic cases of diarrhea [6]. Indeed, two large prospective surveillance studies in the UK and USA identified EAEC amongst the most commonly isolated bacterial species from individuals with diarrhea, with isolation rates similar to *Campylobacter jejuni* and greater than *Salmonella* sp. [8], [9]. Furthermore, a meta-analysis of the previously published case control studies from a variety of different geographical regions clearly supports a role for EAEC in mediating diarrheal disease, and the increasing number of reports in which EAEC is implicated as the agent mediating diarrhea suggest that this is an important emerging pathogen [10].

The clinical features of EAEC-mediated diarrheal infection are normally a watery-mucoid stool, which is only occasionally bloody, with low-grade fever and little or no vomiting [4]. In patients with an active infection, EAEC elicits intestinal inflammation as determined by the presence of proinflammatory cytokines and fecal lactoferrin [11]–[14]. Studies using human intestinal tissue grown *in vitro* have indicated that EAEC has the ability to adhere to both ileal and colonic mucosa and that it can cause mucosal toxicity defined by crypt dilation, microvillous vesiculation and epithelial cell extrusion although such histopathology has not been observed in natural active human infections [15]. However, EAEC do form an aggregative biofilm embedded in a thick mucous blanket and it is this biofilm that may be related to its capacity to cause disease [6], [12].

As not all strains of EAEC elicited diarrhea, the EAEC strain 042 which caused diarrhea in the volunteer study became the prototypical EAEC strain for the study of virulence factors and EAEC pathogenicity [7]. The first virulence factors to be identified were the aggregative adherence fimbriae or AAFs, followed by a wide array of functionally distinct factors including the AafB invasion [11]; a secreted protein called dispersin, which binds LPS thereby neutralising the negative charge of the cell [16]; the plasmid-encoded Pet and EAST-1 toxins [17], [18]; the chromosomally encoded ShET1 toxin [19]; Pic, a mucinase widely associated with pathogenic *E. coli* and *Shigella* sp. [19], and more recently a novel type VI secretion system [20]. Despite these investigations, the genetic basis for EAEC-mediated diarrhea has not been established. Here we report the first complete genome sequence and virulence factor repertoire of EAEC, targeting strain 042, the prototypical member of this pathotype. We also present the results of comprehensive comparative genome studies with all sequenced *E. coli* strains, and comparative metabolic profiling of EAEC strain 042.

## Results and Discussion

### Genetic Complement

#### Genome structure and general features

**Figure 1:**
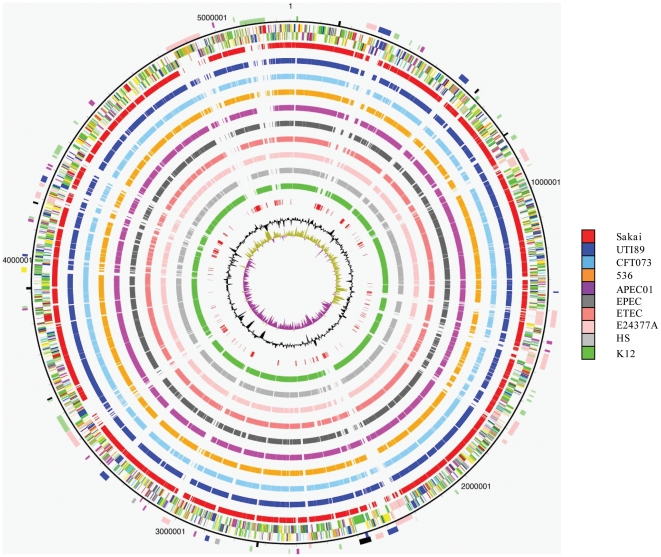
Circular representation of the *E. coli* O42 chromosome. From the outside in, the outer circle 1 marks the position of regions of difference (mentioned in the text) including prophage (light pink) fimbrial operons (Dark green) as well as regions differentially present in other *E. coli* strains: blue (Present in 0157:H7 & absent/divergent in UPEC CFT073) Light Green (Present in 0157:H7 absent/divergent in UPEC CFT073). Circle 2 shows the size in bps. Circles 3 and 4 show the position of CDSs transcribed in a clockwise and anticlockwise direction, respectively (for colour codes see below); circle 4 to 13 show the position of *E. coli* O42 genes which have orthologues (by reciprocal FASTA analysis) in other *E. coli* strains (see methods): Sakai (0157:H7; red), UT189 (UPEC; dark blue), CFT073 (UPEC; light blue), 536 (UPEC; orange), APEC 01 (APEC; dark pink), E2348/69 (EPEC; black), H10407 (ETEC; salmon pink), E24377A (ETEC; pale pink), HS (grey), and K-12 MG1655 (green). Circle 14 sows the position of genes unique to *E. coli* 042 unique (red). Circle 15 shows a plot of G+C content (in a 10 Kb window). Circle 16 shows a plot of GC skew ([G−C]/[G+C]; in a 10 Kb window). Genes in circles 3 and 4 are colour coded according to the function of their gene products: dark green = membrane or surface structures, yellow = central or intermediary metabolism, cyan = degradation of macromolecules, red = information transfer/cell division, cerise  = degradation of small molecules, pale blue  = regulators, Salmon pink = pathogenicity or adaptation, black = energy metabolism, orange = conserved hypothetical, pale green = unknown, brown = pseudogenes.

**Figure 2:**
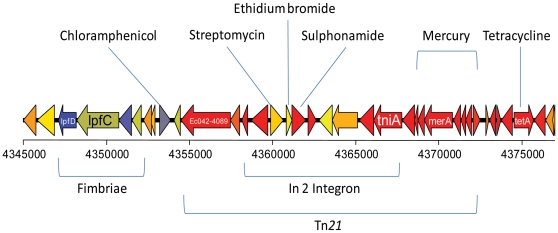
Gene organisation of the Tn21 element containing loci encoding antibiotic resistance. The Tn21 element is inserted between genes *lpfA* and *glmS* and constitutes ROD 66. The presence of this locus is consistent with the phenotypic information garned from the BioLog assays.

The genome of EAEC 042 consists of a circular chromosome of 5,241,977 bp and one plasmid pAA of 113,346 bp. The general features of the EAEC 042 genome are presented in Table 1. A total of 4,886 genes were identified in the chromosome, 100 (2%) of which do not have any match in the database, 556 (11%) are conserved hypothetical proteins, with no known function and only 481 (10%) seem to be mobile elements such as integrases, transposases, or phage related. We have identified 78 genomic islands in EAEC 042 that are differentially distributed/represented and/or sequence diverged among the sequenced *E. coli* genomes, these islands are designated regions of difference (ROD) (Fig. 1; Table S1). The overall size of these RODs is 1.26 Mb (24% of the chromosome). The RODs encode virulence determinants, metabolic proteins, proteins with no obvious functions and mobile elements such as prophage and a conjugative transposon. The conjugative transposon Tn*2411* (within ROD66) is highly similar to Tn*21* and carries a variety of genes encoding antibiotic resistance (Fig. 2). The functional significance of these genes is discussed below. Nine prophage regions, designated 042p1–042p9, were identified in the EAEC 042 genome (Table 2). Four of the prophage were lambdoid in nature (042p2, 042p3, 042p4 and 042p6) and were highly similar to each other however only three (042p1, 042p3 and 042p6) appeared to carry cargo genes (see Fig. S1 and File S1). The content of the remaining ROD are discussed in detail later.

**Table 1:** Major features of the *E. coli* 042 genome.

	Chromosome	Plasmid
**Size (bp)**	5,241,977	113,346
**Predicted CDSs**	4,810	152
**G+C content (%)**	50.56	49.55
**Coding regions (%)**	86.6	80.4
**Average CDS length (bp)**	943	629
**tRNAs**	93	0
**rRNA**	22	0
**Pseudogenes**	110	32

**Table 2:** Characteristics of the prophage elements present in EAEC 042.

ROD	Position	Size	Site of insertion	Cargo genes	Position of cargo genes
**ROD15**	877116..917926	40811	15bp direct repeat	*gtrAB* and Ec042-0853 encoding bactoprenol glucosyl transferase involved in O-antigen modification	914835..917583
**ROD18**	1396781..1450149	53369	Imperfect repeat		
**ROD21**	1533876..1584276	50401	15bp direct repeat	*sitABCD* encoding iron transport proteins	1580324..1583773
**ROD26**	1763886..1811200	47315	Palindromic repeat	Ec042-1724 encoding putative exonuclease	1807070..1809541
**ROD27**	1832521..1843545	11025	Intragenic in Ec042-1748		
**ROD30**	2214943..2256641	41699	tRNA-Arg; 31-mer direct repeat		
**ROD30**	2256892..2272385	15494	tRNA-Met	Ec042-2203 encoding putative exonuclease VIII	2268605..2271046
**ROD34**	2551911..2560079	8169	tRNA-Pro	Ec042-2429, similar to proQ, structural element that influences osmotic activation of proP at posttranslational level; Ec042-2430, PerC-like protein, similar to transcriptional activator of LEE in EPEC/EHEC	2557981..2558798
**ROD60**	4198051..4218142	20092			
**ROD61**	4236703..4247732	11030	tRNA SelC(p)		

On the basis of nucleotide sequence homology, the plasmid pAA belongs to the IncFIIA family. The plasmid includes 152 CDS, of which 32 are pseudogenes. Of the remainder, there are 7 that encode hypothetical proteins with no match in the database, 23 encode conserved hypothetical proteins with no predicted function, 55 have transfer, replication or plasmid maintenance functions, there are 18 mobile element-derived genes that encode transposases, and the remaining 17 CDS have demonstrated or predicted roles in virulence (Table 1 and Fig. S2). Insertions in the plasmid include genes encoding many of the well-characterised EAEC 042 virulence factors and include the cytopathic toxin Pet, the AAF/II aggregative fimbriae, the AggR transcriptional regulator, dispersin and its cognate secretion machinery Aat, and operons encoding a putative iron transport system and a polysaccharide biosynthesis pathway all of which are discussed later.

#### *E. coli* core genome and pangenome

The EAEC 042 genome is largely colinear with that of the previously sequenced *E. coli* genomes except for a few inversions and insertions/deletions (Fig. S3). A box-plot showing the estimated core genome size (i.e. the genes conserved in all *E. coli* strains), as a function of the number of genomes sequenced for 100 randomly selected strain combinations is shown in Fig. S4. An exponential decay curve was fit using the R function nlrq [21], and gave a predicted core genome size of 2356 genes (Table S2). This is larger than the previous estimate of ∼2200 [22], [23], possibly due to our inclusion of genes that are present but unannotated in some strains. The predicted core genome size is close to the number of genes conserved across all the genomes included in this study, suggesting that the number of possible gene deletions is close to saturation, and that further *E. coli* genome sequencing projects are unlikely to identify many novel gene deletions. The analysis indicates an open *E. coli* pangenome, as has been found in previous studies [22], [24], with an estimated 360 new genes being identified with each additional genome sequenced (Fig. S5). The *E. coli* core genome was further compared with the non-*coli Escherichia albertii* and *Escherichia fergusonii*, with 2173 genes found to be conserved (Table S2). Comparisons with the other available intact enterobacterial genomes showed that 967 genes were conserved across the family (Table S2).

#### *E. coli* phylogeny

A phylogeny was constructed based on the concatenated sequences of 2173 genes that are conserved in all *E. coli* strains and in *E. albertii* and *E. fergusonii*, which were included as outgroup sequences. The results are shown in Fig. S6. The established *E. coli* sub-groups (A, B1, B2, D and E) are all monophyletic with the exception of group D, which is divided by the root. *E. coli* strains SECEC SMS-3-5 and IAI39 cluster with group B2, which includes many extraintestinal pathogenic *E. coli* strains, whereas strains EAEC 042 and UMN026 cluster with groups A, B1, E and the *Shigella* strains. This corresponds with the conclusions drawn in a recent MLST study, where it was proposed to classify strains such as SMS-3-5 and IAI38 in a new group F [25]. However, we prefer to designate the two groups as D1 and D2, to retain compatibility with the previous nomenclature and to follow the precedent of group B.

### Metabolic Profiling

*E. coli* K-12 strains, such as *E. coli* MG1655, have been used to characterise many of metabolic pathways we understand today. However, recent publications have described what most *E. coli* biologists have known for some time; due to prolonged laboratory passage and a variety of treatments to remove λ-phage and the F plasmid, *E. coli* K-12 strains are not archetypal strains representing the biology of the genus [26]. The genotype of the *E. coli* K-12 strain MG1655 (F^−^, λ-, *ilvG*, *rfb*-50, *rph*-1) given by the *E. coli* stock Center, reflects only some of the differences between *E. coli* MG1655 and other *E. coli* strains. These differences extend beyond the additional virulence factors carried by pathogenic strains and include central metabolic functions carried by other *E. coli* strains but lost by *E. coli* K-12 strains [26]. To reveal a more representative metabolic profile for *E. coli* strains BioLog Phenotype Microarrays (PMs) were performed on EAEC 042 and compared with similar analyses of *E. coli* MG1655 (Table S3 and S4). The genetic basis accounting for significant differences between the strains are described. The major differences between the strains can be summarized into two main categories: resistance to antimicrobials and differences in nutrient utilization.

#### Antibiotic resistance/drug resistance

Soon after the discovery of the EAEC pathovar it was noted that many clinical isolates of EAEC displayed multiple antibiotic resistance [27]. Antibiotic resistance among EAEC strains is typically higher than among other diarrheagenic pathovars, perhaps accounting for the increasing isolation of EAEC from epidemiologic studies [28]. A variety of studies from geographically distinct areas have reported high levels of resistance to tetracycline, spectinomycin, streptomycin, trimethoprim-sulfamethoxazole and ampicillin [29]–[31]. The antibiotic resistance profile of *E. coli* 042 derived from PMs revealed resistance to sulphonamides, chloramphenicol, aminoglycosides and tetracyclines that was not exhibited by *E. coli* MG1655 (Table S3). This is consistent with the presence on the EAEC 042 chromosome of Tn*2411*, a Tn*21*-like transposon (Fig. 2). Tn*2411* possesses genes encoding resistance to chloramphenicol (*cat*) and tetracycline (*tetA*) and also includes a class 1 integron In2 that carries antibiotic resistance cassettes *aadA1* (streptomycin and spectinomycin), *suI* (sulfonamide) and *emrE* (ethidium bromide) (Fig. 2). Interestingly, the PM data revealed there was no difference between the ability of *E. coli* MG1655 and EAEC 042 to grow in the presence of ethidium bromide even though *E. coli* MG1655 does not possess the Tn*2411* element possessing *emrE*. This can be explained by the fact that *E. coli* MG1655 possesses both the *emrE* (prophage associated) and the predicted multidrug efflux system *emrYK* on the chromosome, genes that are absent in the equivalent sections of the EAEC 042 genome. In addition, the Tn*2411* element also possesses genes for mercury resistance (*merRTPCAD*). However, the PMs do not include an assay for growth in mercuric chloride. Nevertheless, the high identity between the *mer* genes in the Tn*2411* element on the EAEC 042 chromosome and Tn*21* strongly suggests that the EAEC 042 *mer* resistance is functional [32].

The PMs revealed EAEC 042 is more resistant to arsenite and antimony chloride than *E. coli* MG1655. This phenotype is most likely the result of the *E. coli* MG1655 *ars* operon lacking *arsA* (coding for the catalytic subunit of the ATP-driven arsenite/antimonite pump) or *arsD* (the trans-acting transcriptional repressor protein) genes (Fig. S7). Previous work has shown that in the absence of the ArsA ATPase subunit, ArsB confers only partial arsenite resistance by translocating these ions into the periplasm using energy derived either from the proton pumping respiratory chain or from F_0_F_1_ ATPase [33].

**Figure 3:**
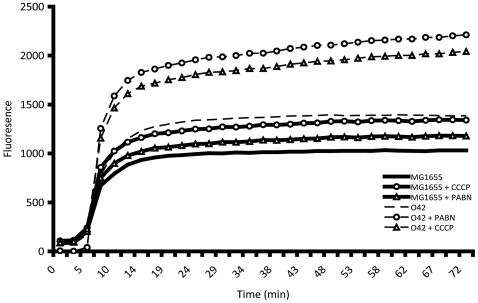
Measurement of small molecule uptake. Hoescht 33342 is a substrate of the major AcrAB-TolC efflux systems. Accumulation by *E. coli* MG1655 and EAEC 042 was measured fluorometrically in the presence or absence of the efflux pump inhibitor PAβN and the proton-motive force inhibitor CCCP. Efflux of Hoescht 33342 is inhibited in the presence of both PAβN and CCCP in EAEC 042 and *E. coli* MG1655. However, Hoescht 33342 accumulates to higher levels in EAEC 042 than *E. coli* MG1655 suggesting EAEC 042 possesses a more permeable membrane.

The PMs revealed that *E. coli* MG1655 is more resistant to acriflavine than EAEC 042. However, both EAEC 042 and *E. coli* MG1655 possess *acrAB* (Ec042-0500/0501) and *tolC* (Ec042-3326) the gene products of which act in concert to form an efflux system that confers acriflavine resistance [34], [35]. To determine whether *E. coli* MG1655 was more efficient than EAEC 042 at effluxing compounds such as acriflavine, the accumulation of Hoescht 33342 was determined. The accumulation of Hoescht 33342 reached a significantly higher steady state within EAEC 042 than *E. coli* MG1655 (Fig. 3). The addition of the efflux pump inhibitor PAβN increased accumulation of the dye by both *E. coli* MG1655 and EAEC 042 although the effect was much greater for the latter (Fig. 3). This indicates that the greater amount of this compound accumulated by EAEC 042 relative to *E. coli* MG1655 is likely to be a result of increased permeability of EAEC 042 rather than lack of efflux activity. Similar results were obtained with the use of carbonyl cyanide-m-chlorophenyl hydrazone (CCCP) a proton motive force inhibitor which inhibits the action of other efflux systems (Fig. 3). Nevertheless, the increase in dye accumulation in the presence of PAβN and CCCP demonstrates that active efflux systems are present in EAEC 042. The higher resistance of *E. coli* MG1655 to acriflavine is thus most likely due to decreased uptake and is perhaps unsurprising as the related dye acridine orange was used to select K-12 derivatives lacking the F plasmid [26].

To validate the genome sequence and PM data the minimum inhibitory concentration (MIC) for acriflavine and a panel of other antimicrobials was determined (Table 3). The PM data was predictive of MIC although this correlation was not absolute. EAEC 042 was significantly more resistant to chloramphenicol, tetracycline, streptomycin and spectinomycin than *E. coli* MG1655, most likely as a result of the carriage of the specific resistance genes. There were no other significant (i.e. two dilutions or greater) differences in susceptibility to any of the other antimicrobials tested between EAEC 042 and *E. coli* MG1655.

**Table 3:** Minimum inhibitory concentrations (mg/L) of antibiotics, dyes and biocides against *E. coli* strains.

	Strain	
Agent	*E. coli* MG1655	EAEC 042
Chloramphenicol	**4** a	**>32**
Tetracyline	**2**	**>32**
Streptomycin	**2**	**>128**
Spectinomycin	**16**	**>128**
Nalidixic acid	**8**	**2**
Ciprofloxacin	<0.015	<0.015
Gentamicin	0.5	0.5
Erythromycin	32	32
Ceftriaxone	0.06	0.06
Cloxacillin	>128	>128
Triclosan	<0.03	<0.03
Ethidium bromide	512	256
Acriflavine	128	64

a Numbers in bold represent significant (greater than two dilution) differences compared to MG1655.

Interestingly, susceptibility testing revealed EAEC 042 was four-fold more susceptible than *E. coli* MG1655 to nalidixic acid. This correlated with the PM data which indicated a reduced ability of EAEC 042 to grow in the presence of nalidixic acid compared to *E. coli* MG1655. PM data also demonstrated that *E. coli* MG1655 is more resistant than EAEC 042 to a variety of β-lactamase antibiotics, rifampicin, macrolides, and some other antimicrobials (Table S4). This could be due to increased uptake of the antibiotics, as described above, or may reflect the increased ability of EAEC 042 to recruit iron, as described below.

#### Iron acquisition

Iron is an essential nutrient for bacterial growth and is a major limitation to successful colonisation within the mammalian host. Mammals have high-affinity iron-binding proteins such as transferrin and lactoferrin that ensure free iron availability is maintained at extremely low levels. Counteracting these protective measures, pathogens have evolved a variety of high-affinity iron scavenging systems such as siderophore production, haem and haemoglobin uptake transporters [35]. We have reviewed differences in iron transport systems between the *E. coli* K-12 strain MG1655 and EAEC 042 and these are summarised in Table S5. Consistent with other pathogenic strains of *E. coli*, EAEC 042 possesses several additional iron-uptake systems compared to *E. coli* MG1655. These systems include the Shu transporter, required for the uptake of haem; the Fit siderophore system for the uptake of ferrichrome; the Yersiniabactin uptake system found on the *Yersinia* high pathogenicity island; the SitABCD system which can recruit iron and manganese, a predicted bacterioferritin and a plasmid-encoded ferric (III) citrate transporter. Previous work has demonstrated that the Yersiniabactin system is intact and functional in EAEC 042 [36]. Moreover, whilst both strains contain the *efeUOB* oxidase-dependent ferrous iron transporter, the *E. coli* MG1655 transporter is non-functional due to a frameshift mutation in *efeU* [37]. To determine whether *E. coli* EAEC 042 was more efficient at sequestering iron we measured the total iron content of mid-exponential phase cultures of EAEC 042 and *E. coli* MG1655 strains grown in Neidhardt's Rich Defined Medium. We found that *E. coli* 042 contained 13.7±0.6 pmoles iron/mg protein compared to 8.15±0.976 pmoles iron/mg protein in *E. coli* MG1655. Thus, *E. coli* 042 contains 1.68-fold more iron than *E. coli* MG1655; a finding consistent with other pathogenic strains of *E. coli* (M. Goldberg, unpublished).

Recent studies by Goldberg and Lund (personal communication) have shown that the enhanced ability of pathogenic strains to scavenge iron can prove disadvantageous under oxidising conditions. Oxidative stress damages iron-binding proteins which results in an increase in free iron levels in the cytoplasm, triggering Fenton reactions [38], [39]. The enhanced free-radical production resulting from this reaction can overwhelm the cells' free-radical scavenging systems, causing further damage to iron binding proteins including the Fur repressor, leading to increased iron uptake and even greater oxidative damage. Cells containing larger amounts of intracellular iron are therefore more likely to succumb to oxidative stress than cells containing less. We found that *E. coli* 042 was more susceptible to the redox-cycling compound menadione (MIC = 1 mg/ml) than *E. coli* MG1655 (MIC = 2 mg/ml) indicating EAEC 042 is more susceptible to oxidative stress than *E. coli* MG1655. The PM data indicated that *E. coli* 042 is more susceptible to gyrase inhibitors (e.g. Nalidixic acid) and β-lactam antibiotics (e.g. Oxacillin, Phenethicillin) than *E. coli* MG1655 (Table S4 and 3). The ability of EAEC 042 to sequester higher levels of iron, in conjunction with the increased susceptibility to the oxidising reagent menadione, strongly suggests the increased susceptibility of EAEC 042 to these antibiotics is due to the triggering of Fenton reactions in a manner previously described by Kohanski and colleagues [39].

#### Carbon source utilization

Bacteria require a sufficient supply of carbon to feed their metabolic pathways. In their native environments heterotrophic organisms encounter limited amounts of complex mixtures of carbon sources that are often present at low concentrations. As a result microbial cells have developed multiple different systems to utilise a wide array of different substrates as carbon sources. Such differences are utilised in diagnostic tests to differentiate between particular species and strains of bacteria. PMs for sole carbon source utilization showed that EAEC 042 can utilise 2-Deoxy-D-Ribose more effectively than *E. coli* MG1655 (Table S5). Previous reports have shown that *E. coli* K-12 cannot use this carbon source, but *Salmonella enterica* serovar Typhimurium can, using the *deoXKPQ* gene products [40]. EAEC 042 possesses homologues of the *S.* Typhimurium *deoXKPQ* (Ec042-4753–4756).

N-acetyl-D-galactosamine and N-acetyl-D-glucosamine are components of intestinal mucin, as well as peptidoglycan. The PM screening demonstrated that EAEC 042 utilises N-acetyl-D-galactosamine and N-acetyl-D-glucosamine better than *E. coli* MG1655 (Table S3), confirming previous work showing that K-12 strains are unable to use these substrates as sole carbon sources [41]. In *E. coli* O157:H7 the genes for N-acetyl-D-galactosamine and N-acetyl-D-glucosamine utilisation were identified as the *agaZVWEFASYBCDI* gene cluster. In *E. coli* MG1655 a portion of this locus is missing due to site-specific recombination between *agaW* and *agaA* [42], however this locus is present and intact in EAEC 042 (Fig. S8). This observation is consistent with the recent demonstration that EAEC 042 can utilise intestinal mucin as a carbon source [43].

L-sorbose utilization by pathogenic *E. coli* and *Shigella* differs between strains [44], [45]. The PM data shows that EAEC 042 is significantly better at utilising L-sorbose than *E. coli* MG1655 (Table S3). Genome comparison between the two strains (Fig. S9) shows that EAEC 042, like other *E. coli* and *Shigella* pathotypes carries the *sorEMABFDC* operon (Ec042-4384–4390), located between *ybiC* and *rluF*. *E. coli* MG1655 does not carry this operon. BLAST analysis of the CDS in this region in EAEC 042 confirmed that the genes have high identity to previously described *sor* genes and thus are presumed to be functional.

Conversely, PM data showed that *E. coli* MG1655 grows and metabolizes D-Serine, Mucic acid (D-galactarate), β-D-Allose and D-Xylose more effectively than EAEC 042 (Table S4). A previous report has highlighted extensive genomic variability in the *argW*-*dsdCXA* genomic island in *E. coli* strains [46]. *E. coli* MG1655 has the *dsdCXA* gene cluster that codes for the ability to utilise D-serine, whereas EAEC 042 lacks these genes which accounts for the metabolic difference in serine utilization between the strains. Alignment of the *gar* operon from *E. coli* MG1655 which encodes the galactarate metabolic operon [47], [48] with the equivalent region of the EAEC 042 genome shows that the single ORF in *E. coli* MG1655 encoding the D-galactarate dehydrogenase enzyme (*garD*) is two CDS in EAEC 042, suggesting that enzyme function and hence metabolism of D-galactarate will have been disrupted in EAEC 042 by a mutation in this gene (Fig. S10). EAEC 042 is also defective in D-Allose metabolism, compared to *E. coli* MG1655 in the PMs. Alignment of the genomes centred on the *als* operon (Fig. S11) [49], [50] shows complete absence of the *als* operon (*rpiB*, *rpiR*, *alsBACE* and *K*) in EAEC 042. EAEC 042 also shows a lower level of metabolism compared to *E. coli* MG1655 when xylose is used as a sole carbon source. This can be explained by the absence from EAEC 042 of the *xylE* gene encoding the Major Facilitator Superfamily low-affinity xylose proton symporter. There is a second xylose uptake system, an ABC transporter (*xylFGH*) present in both strains which has been reported to be the dominant xylose transport system under both aerobic and anaerobic conditions [51] and functionality of this system leading to a reduced, but nonetheless effective uptake of xylose is consistent with the metabolic differences between *E. coli* MG1655 and EAEC 042 when xylose is the sole carbon source.

**Figure 4:**
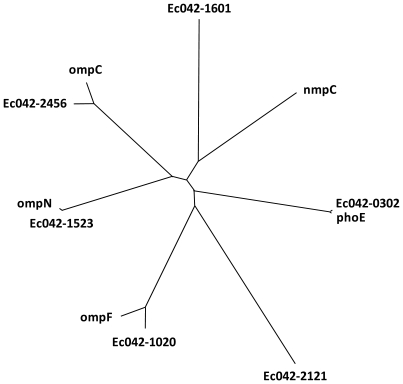
Phylogenetic analyses of the porin CDS from EAEC 042 and *E. coli* K-12. The CDS encoding *ompN*, *ompC*, *ompF* and *phoE* demonstrated little divergence. By comparison that encoding *ompD*(*nmpC*) demonstrates greater divergence and Ec042-2121 is present on an evolutionary distinct lineage.

Many of the phenotypic differences between EAEC 042 and *E. coli* MG1655, which were observed in the PMs, do not have an easily identifiable genetic basis. However, the increased ability of EAEC 042 to take up certain compounds, as measured in the PAβN experiments (Fig. 3), may explain why EAEC 042 is capable of metabolizing several compounds that *E. coli* MG1655 is not. The normal pathway for uptake of small molecules is via porins [52], [53]. Porins act as molecular sieves to allow passive diffusion of low molecular weight solutes (<600 Da) into the cell. Although structurally similar, the different porins have differing pore sizes, ionic selectivity and expression profiles allowing the bacterium to adapt to the variable environments [54]–[56]. In contrast to *E. coli* MG1655, which possesses four porin genes (*ompF*, *ompC*, *phoE* and *ompN*) and one pseudogene (*nmpC*/*ompD*), EAEC 042 possesses six intact genes encoding porins. Like *E. coli* MG1655, EAEC 042 possesses *ompF* (Ec042-1020), *ompC* (Ec042-2456), *phoE* (Ec042-0302) and *ompN* (Ec042-1523), but it also possesses an apparently functional *ompD* (Ec042-1601) and an additional phylogenetically distinct porin (Ec042-2121) that is differentially represented amongst pathogenic *E. coli* but whose precise function is unknown (Fig. 4 and Fig. S12). In addition to the important physiological roles played by porins, these molecules are under constant selective pressure due to their recognition by the phages, colicins and the immune system. Indeed, OmpD from *S. enterica* Typhimurium was recently shown to be a key target of a protective T-independent antibody response and its universal presence amongst non-typhoidal *Salmonella* suggest it plays an important role in the ability of enteric organisms, such as EAEC 042, to persist in the intestine and interact with the host [57].

**Figure 5:**
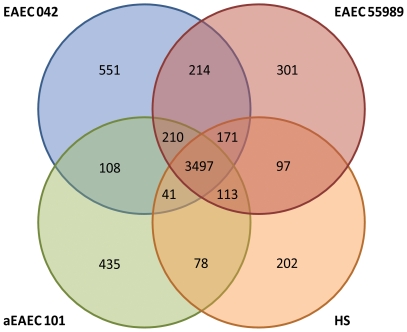
Comparison of the genetic content of the three genome sequenced EAEC isolates (042, 55989 and 101) with the commensal strain *E. coli* HS. The four strains share a large proportion of common genes. Only 210 EAEC specific genes were found (see text for details).

### Host-Pathogen Interactions

Pathogenic bacteria produce a wide array of virulence factors which allow the organism to colonise a specific niche within the host and to mediate disease. The majority of these factors are proteins. These proteins are either secreted factors, which interact directly with the host, or form part of the machinery required for translocation of the secreted molecules to the exterior of the bacterial cell [58]. To ascertain if specific virulence factors are associated with the clinical characteristics of EAEC-mediated diarrhea, the repertoire of genes from three EAEC strains were compared with the commensal *E. coli* strain HS (Fig. 5). *E. coli* HS was chosen as a base for comparison rather than the lab adapted *E. coli* strain K-12; current dogma suggests genes responsible for pathogenesis are absent from commensal isolates. These analyses revealed 3497 CDS (72.7%) in EAEC 042 that are common to all four sequenced *E. coli* genomes, and 210 (4.4%) EAEC-specific genes (Table S6). However, detailed analyses of the EAEC-specific genes revealed that 120 were mobiles elements, 53 were present in the lab strain *E. coli* K-12, 18 were involved in O-antigen/colonic acid biosynthesis and 6 were pseudogenes. Only 13 CDS represented genes which might be labelled virulence factors viz. the putative polysaccharide biosynthetic *shf* locus (Ec042-4770–4772), the iron recruitment *fec* locus (Ec042-4776–4782) and three genes of a previously described Type VI secretion locus (Ec042-4562, Ec042-4563 and Ec042-4571); these are discussed in detail later. The paucity of conserved virulence factors reflects the previously described phylogenetic heterogeneity of EAEC [59]. The 551 EAEC 042-specific genes represent many of the previously described virulence factors discussed below.

### Protein Transport

EAEC 042 possesses the major inner membrane translocation machines viz. Tat (Ec042-4216–18) and Sec, for export of proteins to the periplasm. It is not surprising to find within EAEC 042 a complete repertoire of *E. coli* Sec components as many are essential for survival (e.g. SecY) and the non-essential components (e.g. SecB) are required for efficient functioning of the cell. In contrast, not all bacteria possess the Tat system and none of the Tat system components are essential for the survival of *E. coli* [58], [60]. Screening of all EAEC 042 CDS using TatP and TatFind identified a number of substrate molecules present within EAEC 042. However, none were unique to EAEC 042, and all had homologues within the non-pathogenic laboratory strain *E. coli* K-12, suggesting EAEC 042 does not possess any virulence factors which require the Tat system for secretion.

All members of the major protein secretion systems (Type 1–6 and the Chaperone-Usher pathways) required for translocation of proteins to the exterior of the cell are represented in EAEC 042 [58]. The Type 4 secretion system encodes the plasmid conjugation system (Fig S2) and will not be discussed further.

#### Chaperone-usher/fimbriae

*E. coli* 042 possesses 11 chaperone-usher family fimbrial operons on the chromosome and an additional system on the plasmid (Fig. S13). The number of these operons in EAEC 042 is similar to those of other *E. coli* strains. Although the chromosomally-encoded fimbrial operons are found in at least one of the previously sequenced *E. coli* strains, they display significant sequence divergence, particularly between the fimbrial adhesins and fimbrial structural subunits, presumably due to immunological pressures. No definitive explanation exists for such a large repertoire of fimbrial adhesins however it has been suggested for *Salmonella*, which possesses a similar diversity in fimbrial systems, that the differing systems reflect adaptation to colonizing different hosts; an alternative explanation may be that they are required for colonising different sites within the same host [61].

The AAF/II plasmid encoded fimbrial system is one of the better characterized virulence factors of EAEC 042. The fimbriae belong to the Dr family of fimbrial adhesins. Expression of the fimbrial genes is regulated by *aggR*, an AraC class of transcriptional activator, which also regulates a variety of chromosomally encoded genes. The fimbriae are essential for biofilm formation and mediating interaction with host cells while AafB has been implicated in epithelial cell invasion [62]. The adherence phenotype mediated by the AAF/II fimbriae is modulated by the Type 1 secreted surface protein termed dispersin, which is described below.

#### Type 1 secretion systems

In addition to the secreted substrate protein, normal Type 1 protein secretion systems (T1SSs) of Gram-negative bacteria are composed of three basic subunits viz. a TolC-like outer membrane pore-forming protein (OMP) and two inner membrane associated proteins, respectively termed the membrane-fusion protein (MFP), which contacts with the TolC-like protein, and the ATP-binding cassette (ABC) protein, which transduces energy to the system [58]. EAEC 042 possesses three T1SSs: the plasmid-encoded *aat* locus and two chromosomally-encoded systems (Ec042-0526–0531 and Ec042-3196–3202) [16]. The *aap* gene (Ec042-pAA055) encodes Dispersin, the secreted substrate protein for the plasmid encoded T1SS. In contrast to the normal T1SSs, the *aat*-encoded dispersin secretion system, while containing MFP (AatD; Ec042-pAA011), ABC (AatC and AatP; Ec042-pAA007 and 010) and OMP (AatA; Ec042-pAA008) proteins, also contains an additional component (AatB; Ec042-pAA009) whose function is unknown [16].

The Ec042-0526–0531 locus is homologous to a syntenic locus in *E. coli* O157:H7 and possesses genes encoding an RTX toxin, a TolC-like OMP, an MFP, an ABC protein and a CueR-like transcriptional regulator (Fig. S14). The substrate molecule for both systems resembles an RTX family exoprotein however in both systems it is frameshifted; in EAEC 042 it is represented by CDS Ec042-0527–0530. The Ec042-3196–3202 locus encodes genes for the synthesis and secretion of the H47 ribosomally-encoded peptide microcin antibiotic [63]. While this locus encodes an MFP and an ABC protein for secretion of the peptide, in contrast to the RTX locus it does not encode a TolC-like OMP. Previous studies have demonstrated that such systems can commandeer the chromosomally encoded TolC protein (Ec042-3326) and this is supported by the demonstrable promiscuity of TolC interaction with other MFP and ABC proteins responsible for efflux of a wide variety of substrate molecules e.g. AcrAB [34].

#### Type 2 secretion systems

CDS Ec042-3242–55 encode a complete Type 2 secretion system (T2SS). Recent investigations have demonstrated that this locus is transcribed from a promoter upstream of Ec042-3255 resulting in a polycistronic mRNA coding for all components of the T2SS [64]. Comparisons with the other sequenced *E. coli* genomes reveal that the location and sequences of the genes encoding this T2SS are conserved in many pathogenic strains of *E. coli* (Fig. S15). *E. coli* K-12 possesses *yghJ*-*gspO*(*pppA*)-*gspC*(*yghF*) and the distal *gspL*-*gspM* genes but not the remainder of the genes suggesting that this locus has undergone genetic attrition in certain strains and acquisition of the genes predates divergence of genus *E. coli* (Fig. S15). The locus was previously described for ETEC H10407 and is essential for secretion of heat-labile enterotoxin (LT) [65]. However, neither EAEC 042 nor many of the other strains possess LT-toxin suggesting that this T2SS has alternate substrate molecules. Analysis of Ec0420-3255 (*yghJ*) reveals homology to AcfD, a colonization factor of *Vibrio cholerae* that is part of the Tcp T2SS responsible for secretion of cholera toxin, a toxin homologous to *E. coli* LT [66]. AcfD, like YghJ, is a lipoprotein suggested to be involved in the normal functioning of the T2SS. However, the lipoprotein sorting signal is similar to the secreted T2SS substrate molecule PulA from *Klebsiella oxytoca* [67] suggesting that YghJ is in fact a secreted extracellular lipoprotein and the substrate of this T2SS, though of unknown function.

#### Type 3 secretion systems

Certain *E. coli* rely on the T3SS to produce flagellar systems and to inject effector proteins into host cells [68]. EAEC 042 possesses the genes for an intact flagellar system encoding the H18 serotype. The EAEC 042 genome also contains an additional flagellar gene cluster, Flag-2, that potentially encodes a second flagellar system, similar to lateral flagellar systems in other bacteria. However, a frame shift disruption in an essential flagellar gene *lfgC* has probably rendered this system non-functional in EAEC 042 [69].

As reported previously, the EAEC 042 genome contains two non-flagellar T3SS gene clusters, ETT2 (EC042-3044–3075) and *eip* (Ec042-4007–4012), inserted next to *glyU* and *selC* tRNA genes, respectively. ETT2 has been proposed to be an active system due to the absence of obvious ablating mutations [70], [71]. We have identified several CDS that encode known and potential effectors that could be secreted via these systems (Table S7). Most of these are orthologues of EHEC and ETEC genes but few are unique to EAEC 042 relative to all the analysed *E. coli* genomes (Table S7). The gene product of EC042-3075, encoded at one end of the main ETT2 gene cluster, shows homology to glucoamylases and has been tentatively identified as a potential ETT2 effector [72]. Searches for T3SS effector genes outside the *glyU* and *selC* islands failed to identify any of the phage-encoded effectors that predominate in EHEC. However, it has been suggested that non-phage-encoded effector genes in *E. coli* encode currently or formerly active ETT2 effectors. Consistent with this view, we found that 15 of the 19 non-phage-encoded effector genes from the EHEC O157 genomes have positional orthologs in the EAEC 042 genome (Table S7). Plus the EAEC 042 genome houses one additional non-phage-encoded effector gene: Ec042-1240, which encodes leucine-rich repeats and is a relative of the *ipaH* gene family from *Shigella* spp. At least four previously undescribed putative effector genes disrupted by frame shifts in EHEC are apparently intact in the EAEC 042 genome (Ec042-1600, Ec042-4064, Ec042-4074, Ec042-4075), adding weight to the idea that the ETT2 T3SS is still active or was recently so. Experiments to demonstrate roles for these novel effectors are under way.

The T3SS of EPEC has been demonstrated to interact with intimin in a Tir dependent manner to promote intimate attachment to the host cell. While no Tir like molecules could be identified in EAEC 042, several intimin-like proteins are present viz. Ec042-0333, Ec042-2220 and three outer membrane proteins, Ec042-2711–2713, similar to proteins on CS54 island of *S.* Typhimurium. The mechanism by which these molecules are secreted remains enigmatic, however it is clear they do not exit via the T3SS and recent publications suggest a secretion mechanism analogous to the T5SS [73].

#### Type 5 secretion systems

Based on differences in the modes of biogenesis the Type 5 secretion system (T5SS) has been divided into three subclasses termed the autotransporters (AT-1; T5aSS), the two-partner secretion system (TPS; T5bSS) and the trimeric autotransporters (AT-2; T5cSS) [58], [74], [75]. EAEC 042 appears to possess the full complement of T5SS encoding genes including 14 AT-1 systems, one TPS system and two AT-2 systems (Fig. S16).

Members of the classical AT-1 autotransporter family possess a conserved architecture of five domains [58]. The two major domains are the functional secreted passenger, or α, domain, which may be released into the extracellular milieu or remain attached to the cell surface, and the β-domain, an integral OMP which mediates secretion of the passenger domain across the outer membrane [58]. EAEC 042 possesses 14 genes encoding AT-1 proteins with a repertoire almost identical to that of *E. coli* O157:H7 (Fig. S16). Two genes encode the previously described mucinase Pic (Ec042-4593) and the plasmid encoded toxin Pet (Ec042-pAA035), both of which are serine proteases secreted into the extracellular milieu; Pic cleaves mucin and confers fitness for intestinal colonization whereas Pet is a toxin discussed later [19], [43], [76]. In contrast, the remaining genes encode surface associated AT-1 proteins [77].

**Figure 6:**
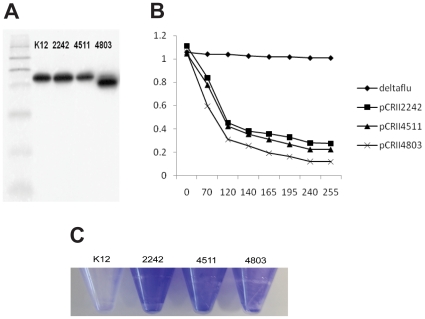
Functional characteristics of the EAEC 042 *agn43* alleles. The three Antigen 43 encoding genes (Ec042-2242, Ec042-4511 and Ec042-4803) were cloned into a high copy number vector (pCRII-TOPO) and expressed in *E. coli* TOP10. (A) Western immunoblot of *E. coli* outer membrane fractions probed with anti-α^43^ antisera demonstrating all three alleles produced cross reacting species. As expected from nucleotide sequence analysis Ec042-4803 produces a smaller passenger domain than Ec042-2242, Ec042-4511 and Antigen 43 from *E. coli* K-12. Autoaggregation assays (B) and biofilm assays (C) of *E. coli* expressing each *agn43* allele demonstrated that each gene product was capable of promoting autoaggregation or biofilm in a manner similar to that previously described. There was no appreciable difference between the proteins in their capacity to induce autotaggregation or biofilm formation. A strain lacking a functional *agn43* allele (*E. coli* deltaflu) failed to autoaggregate and failed to form a biofilm.

EAEC 042 possesses three copies of *agn43* encoding an autotransporter termed Antigen 43 (Ec042-2242, -4511, -4803) which was previously implicated in the ability of *E. coli* to cause disease [78]. Two of these genes (Ec042-2242 and 4511) are closely related (87% identity) while the other gene is more divergent (72 and 65% identity, respectively). Interrogation of Genbank reveals that multiple alleles of *agn43* can occur within a single strain and such occurrences are not limited to any one branch of the *E. coli* phylogeny [78]. No absolute correlation exists between the clinical disease manifested by a particular strain of *E. coli* and the presence or absence of a particular allele of *agn43*. As recently reported, the dichotomy in the grouping can be explained by a region of significant diversity, encompassing the C-terminus of α^43^ and the N-terminus of β^43^, overlapping the point of cleavage between the two domains. Thus, while Ec042-2242 and Ec042-4511 possess the empirically determined cleavage site Ec042-4803 does not. Nevertheless, no defect in the processing of the Ag43 protein derived from Ec042-4803 was detected and expression of Ec042-4803 promoted biofilm formation and cell-cell aggregation in a manner similar to expression of the other alleles (Fig 6). Both allelic groups possess promoters containing three GATC sites with similar spacing, suggesting that members of both families undergo reversible phase variation in a deoxyadenosine methyltransferase- and OxyR-dependent fashion [78].

Four of the CDS (Ec042-1219, -1258, -2590 and -1642) appear to encode non-functional AT-1 proteins while the remaining 6 CDS are variably present in pathogenic and non-pathogenic *E. coli* strains suggesting that these genes may be important for the ability of *E. coli* to colonise the gut. A reasonable explanation for the varying nature of the AT-1 genes (as intact or pseudogenes) across the breadth of the *E. coli* phylogeny may be functional redundancy; many of these proteins have been shown to perform the same *in vitro* functions i.e. cell-cell aggregation and biofilm formation, thus loss of one gene may be compensated for by the presence of another [77].

**Figure 7:**
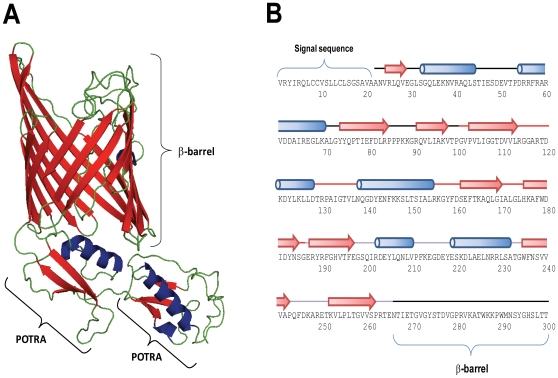
Structural predictions of YtfM. (A) The conserved TpsB homologue YtfM was modelled using SWISS-MODEL and is predicted to form an outer membrane β-barrel structure with two POTRA domains extending into the periplasm. The model was based on the crystal structure of FhaC, the well-characterised TpsB translocator of *B. pertussis* filamentous haemagglutinin; the first POTRA domain could not be modelled. (B) Secondary structure predictions of the N-terminal domain of YtfM using PsiPred predicts the presence of three POTRA domains based on the structural motif β–α–α–β–β; the POTRA domains are highlighted by red, green and cyan lines. The amino acid sequence corresponding to the β-barrel is truncated. Arrows corresponds to β-strands whereas α-helices are depicted by cylinders.

In contrast to the AT-1s discussed above, the TPS system consists of two separate proteins; an outer membrane β-barrel protein (TpsB) and a cognate substrate protein (TpsA) both containing signal sequences to mediate inner membrane translocation. Interrogation of the EAEC 042 genome reveals two genes encoding proteins bearing similarity to the TpsB proteins i.e. *yaeT* (Ec042-0175) and *ytfM* (Ec042-4702), a gene of unknown function [79], [80]. YaeT is an essential protein which plays an unknown role in recruiting β-barrel OMPs into the OM [80]. It exists in complex with a number of accessory factors and consists of a periplasmically located repetitive domain comprising five POTRA repeat motifs and a C-terminal integral OM β-barrel domain [80], [81]. Like YaeT, the TpsB proteins also consist of a periplasmically located POTRA domain (two POTRA repeats) and an OM β-barrel domain. However, in contrast to the YaeT proteins, the TpsB proteins do not form heterooligomeric complexes and while YaeT is promiscuous for OMPs in general, mediating insertion of OMPs into the OM, TpsB proteins are selective for their genetically linked TpsA protein and allow for translocation across the OM [58]. Phylogenetic analyses reveal that YtfM is related to both YaeT and TpsB proteins but forms a distinct cluster with a group of homologous proteins [82]. Tertiary structural predictions indicate that YtfM adopts a similar structure to YaeT and TpsB proteins, possessing two POTRA repeats and a β-barrel domain (Fig. 7). However, secondary structure predictions indicate YtfM possesses three POTRA domains; the first POTRA could not be modeled against known structures (Fig. 7). Immediately, downstream of *ytfM* is *ytfN* (Ec042-4703). The genetic linkage of these genes is universally conserved in *E. coli* and a wide range of Gram-negative bacteria (Fig. S16). The conserved linkage of these molecules, and the identical phenotypes derived for deletion of each of these molecules suggests YtfN is a substrate molecule for YtfM analogous to the TpsA protein. Further support for the theory that YtfM/N form a TPS system is derived from the demonstration that both possess signal sequences for location to the periplasm. YtfM does not function in a manner analogous to YaeT as it is not essential for viability and the levels of OMPs present in the OM are not affected [79]. However, in contrast to the TpsA molecules, YtfN does not possess a conserved TPS motif suggesting the targeting of this molecule to YtfM is different to the TPS systems characterized to date. Furthermore, analysis of YtfN with the BetaWRAP program reveals that YtfN possesses only a partial β-helix structure, a structural organization which appears to be a feature of TpsA proteins. While the function of this locus remains elusive, this locus deserves significantly more attention as several studies using a variety of Gram-negative bacteria with mutations in either YtfM or YtfN have shown those organisms to be severely attenuated in infectious disease models suggesting use as a target for therapeutic or preventive interventions [83]–[85].

EAEC 042 possesses genes encoding two trimeric AT-2 proteins (Ec042-0536 and 3912). Ec042-3912 is orthologous to the recently characterized UpaG protein of UPEC CFT073 [86]. This gene transcends the *E. coli* phylogeny and is present in *E. albertii* suggesting acquisition before divergence of the *E. coli* species. Like many of the classical AT-1 proteins, UpaG mediates cell-cell aggregation, biofilm formation and adherence to epithelial cells. Furthermore, like other members of the AT-2 family, it binds extracellular matrix proteins [86]. The presence of UpaG in the commensal *E. coli* HS suggests that UpaG does not play a significant role in disease *per se* but may be required for efficient colonization of the gut. However, recent studies have suggested that immunization with UpaG mediates a protective effect against challenge with virulent extraintestinal isolates of *E. coli* in mouse models of infection [73]. The literature provides no clues to the function of Ec042-0536.

#### Type 6 secretion systems

Within the genome of 042, we found three predicted type VI secretion systems (T6SS). T6SS are found in a wide variety of Gram-negative pathogens and symbionts [87], and it is speculated that one of the associated proteins, called Hcp, forms a tube that is capable of translocating effector proteins into host cells. A few translocated proteins have been identified, and the best characterized are part of a protein family called “Vgr” for valine-glycine repeat protein. There are eight homologs conserved between all three T6SSs, however there is little shared synteny within these clusters (Fig. S17).

Two of the T6SSs identified (Ec042-4524–4555 and approximately 4 kb downstream Ec042-4562–4577) have been previously characterized with the latter locus under the control of AggR [88]. Interestingly, Ec042-4562, Ec042-4563 and Ec042-4571 are amongst the few genes conserved within EAEC strains 042, 101-1 and 55989. The third T6SS (Ec042-0210–0226) encoded within the EAEC 042 genome is inserted at the *aspV* tRNA locus. This T6SS is more widely distributed than the other two, and the ca. 23 kb region encoding these genes is >95% identical on the nucleotide level to genomes of *E. coli* urinary tract isolates UMN026, 536, and UTI89, avian isolates APEC O1 and BEN2908, the cerebro-spinal fluid isolate S88, and fecal isolates ED1a and EAEC 55989 [89]–[92]. There is no experimental evidence that these genes are expressed. This locus is also the only one of the three that encodes a putative Forkhead-associated domain protein (Ec042-0219), which is proposed in the T6SS of *Pseudomonas aeruginosa* [93] to be phosphorylated by a serine-threonine kinase in response to an environmental cue, resulting in activation of secretion. However, no kinase or phosphatase capable of modulating a phosphorylation event was found encoded within this putative T6SS.

### Toxins

The pAA plasmid carries the genes for two toxins, the plasmid-encoded toxin Pet, and EAST-1 (Ec042-pAA040) (Fig. S2). The well characterized Pet is a serine protease autotransporter (SPATE) secreted by EAEC 042 which induces dilation of crypt openings and rounding and extrusion of enterocytes in human tissue explants [15], [18]. Once secreted Pet exerts its toxic effects by being internalized into host cells where it cleaves the host cytoskeletal protein spectrin [94], [95]. While Pet may play a role in EAEC mediated disease it is only present in a minority of strains [59]. In contrast, EAST-1, encoded by the *astA* gene adjacent to *pet*, is present in a wide variety of strains and different pathovars [59]. EAST-1 induces an increase in short circuit current in Ussing chambers indicative of a net anion secretion [96], [97]. However, the presence of EAST-1 in EAEC 17-2, a strain which did not cause diarrhea in volunteers, suggests that EAST-1 is not the sole mediator of diarrhea, an observation confirmed by studies which demonstrated EAST-1 from EAEC 17-2 had identical activities to EAST-1 derived from EAEC 042 in *in vitro* models of toxicity [7], [97].

In addition to Pet and EAST-1, EAEC 042 chromosomally encodes at least two additional toxins, ShET1 and HlyE. HlyE (Ec042-1231) is a 34-kDa, predominantly α-helical, protein which oligomerises into higher order structures to form a pore-forming toxin mediating cytolytic and cytopathic effects on cultured human cells [98]. Several lines of evidence, including a complex regulatory circuit and recognition by convalescent antisera, indicate a role in disease [99], [100]. However, the occurrence of *hlyE* amongst non-pathogenic bacteria [101] suggests that if HlyE plays a role in mediating disease then it is a minimal role. In contrast to HlyE, the ShET1 toxin is a subunit toxin encoded by *setA* and *setB* (Ec042-4593a,b), which are thought to form an oligomeric toxin consisting of a single 20-kDa SetA protein associated with a pentamer of SetB subunits [19]. ShET1 appears to induce intestinal secretion via cAMP and cGMP, however the precise mechanism of action and detailed biochemistry remains elusive. Unusually, the *setAB* genes are encoded within the *pic* gene but on the complementary strand and thus have the same prevalence characteristics and disease associations as *pic* [19].

### Surface Polysaccharides

Polysaccharides are major determinants of virulence in Gram-negative bacteria. Surface exposed polysaccharides play a dual role mediating interaction of the bacterium with the environment whilst creating a barrier to noxious substances [102]. EAEC 042 possesses four major loci involved in polysaccharide synthesis which distinguishes it from *E. coli* K-12 laboratory strains. These are Ec042-2270–99, which are responsible for synthesis of the O44 serotype O-antigen of lipopolysaccharide, two copies of the Shf locus encoding a myristoyl transferase for modifying the lipid A moiety of LPS (Ec042-4769–72 and Ec042-pAA021–023) and a locus encoding capsular polysaccharide (Ec042-3230–40).

Analysis of the CDS from Ec042-3230 to Ec042-3240 revealed the presence of a Group 2 capsule gene cluster with conserved regions 1 and 3 flanking a central variable region 2 encoding for the biosynthesis of the particular capsular polysaccharide (Fig. S18). Genes Ec042-3230–3235 encode proteins that were 99% identical to the KpsF, E, D, U, C and S proteins encoded by region 1 of group 2 capsule gene clusters involved in polysaccharide export. Likewise Ec042-3239 encoded a protein that was 96% identical to KpsT while the protein encoded by Ec042-3240 was 99% identical to KpsM indicating the presence of an inner-membrane ABC capsular polysaccharide exporter [102], [103]. Three CDS are present in the central region 2 and are likely to be important in the biosynthesis of the particular capsular polysaccharide expressed by this strain of *E. coli*. No capsule has been described for EAEC 042 yet this locus is apparently intact and is thus worthy of further exploration.

The chromosomal *shf* locus consists of four monocistronically transcribed genes previously designated *shf*, *rfbU*, *virK* and *msbB2*. In contrast, the plasmid-encoded locus contains only *shf*, *rfbU* and *virK* but displays 98% nucleotide sequence identity with the chromosomal locus over 3155 bp (Fig. S19). Both loci possess the previously identified PhoP/Q, magnesium and temperature regulated promoter with no interruptions in the CDS suggesting both loci can be transcribed to produce the three proteins [104]. The function of the locus has remained enigmatic. A recent investigation of the locus revealed an insertion in the plasmid copy of *shf* diminished biofilm formation of EAEC 042 but that deletion of the downstream genes did not [105]. This observation is difficult to reconcile since the locus is monocistronic and since a second copy of the gene exists on the chromosome. However, the demonstrated role of RfbU and MsbB2 in LPS modification suggests that this locus is likely to be involved in altering the cell surface through modification of various domains of LPS but only under specific environmental conditions. A variety of studies have demonstrated that this locus is widespread amongst EAEC isolates, and given its important role in the ability of other organisms to cause disease it warrants further study in relation to EAEC pathogenesis [59].

### Conclusion

EAEC is an increasingly recognized enteric pathogen, implicated in diverse clinical and epidemiologic scenarios. Full understanding of the pathogenesis and epidemiology of this organism has been hampered by considerable genomic diversity of clinical isolates. The heterogeneity of virulence was clearly demonstrated in volunteers, but the basis of this heterogeneity has not been characterized. Here, we present the first comprehensive genomic analysis of the prototype strain 042, which was shown to be virulent in adult volunteers. Though our analyses do not yet suggest the basis of this enhanced pathogenicity, the genome of 042 was found to possess many genetic characteristics of pathogenic *Shigella*, *Salmonella* and diarrheagenic *E. coli* strains. These factors include (but are not limited to) apparently complete type II, III, and VI secretion systems (including likely effectors), multiple autotransporter proteins, several proven and putative adhesins, polysaccharide and lipopolysaccharide modification loci, and iron scavenging systems. Comparative functional genomics, where the phenome of the organism can be related to the genome, has shown that some of the differences between EAEC 042 and *E. coli* MG1655 can be related to other genomic differences, often due to loss of single genes, or simple mutations, but also because whole operons are not present in one strain or the other, or that EAEC 042 has acquired a mobile genetic element. This series of experiments demonstrates the feasibility of whole genome-phenome comparisons in search of roles for genes of unknown function. Our studies have provided substantial grist for further experimental studies of EAEC pathogenesis.

## Materials and Methods

### Bacterial Strain and Sequencing

The EAEC O44:H18 strain 042 was isolated from a child with diarrhea in the course of an epidemiologic study in Lima, Peru, in 1983 and was subsequently shown to cause diarrhea in adult volunteers. The sequenced strain was obtained from the original stock kept at the Center for Vaccine Development and was subjected to minimal laboratory passages. The whole genome was sequenced to a depth of 8× coverage from pUC19 (insert size 2.8–5 kb) and pMAQ1b (insert size 5.5–10 kb) small insert libraries using dye terminator chemistry on ABI3700 automated sequencers. End sequences from larger insert plasmid (pBACe3.6, 20–30 kb insert size) libraries were used as a scaffold. The sequence was assembled and finished as described previously [3].

### Gene Prediction, Annotation and Comparative Analysis

CDSs were first identified using GeneHacker followed by manual inspection of start codons and ribosome binding sequences of each CDS. Intergenic regions of >150 bp were further reviewed for the presence of small CDSs encoding proteins with significant homology to known proteins. Functional annotation of the CDSs was made on the basis of results of homology searches against the public non-redundant protein database (http://www.ncbi.nlm.nih.gov/) by BLASTP. Genes for tRNAs, tmRNA, rRNAs and other small RNAs were identified by using the Rfam database through the Rfam website (http://www.sanger.ac.uk/Software/Rfam/index.shtml). We also searched the EAEC 042 genome for all the RNA genes that have been identified in *E. coli* K-12 and Sakai by BLASTN. The annotated genome sequences of EAEC 042 have been deposited in the public databases database (accession numbers: N554766 for EAEC 042 complete genome and FN554767 for the EAEC 042 plasmid pAA).

A search for type-III-secretion effectors in the genome was performed using a previously described set of effectors as the input to BLASTP searches of EAEC 042 CDS predictions [106]. In addition, comparisons of the EAEC 042 and EHEC genomes via the xBASE facility were used to identify positional orthologs of known EHEC effectors [107].

### *E. coli* Core Genome and Pangenome

To assess the size of the core genome and pangenome of *E. coli* a set of 24 complete or almost complete *E. coli* and *Shigella* genomes was selected. These included the complete genome sequences of *E. coli* strains K-12 MG1655 (GenBank accession U00096), O157:H7 EDL933 (AE005174 and AF074613), CFT073 (AE014075), SMS-3-5 (CP000970–4), APEC O1 (CP000468, DQ381420 and DQ517526), E24377A (CP000795–801), 536 (CP000247), C ATCC8739 (CP000946), UTI89 (CP000243–4), HS (CP000802), EDa1 (CU928162), IAI1 (CU928160), IAI39 (CU928164), S88 (CU928161), UMN026 (CU928163), EAEC 042 (N554766), E2348/69 (FM180568–70) and H10407, together with those of *Shigella sonnei* Ss046 (CP00038–39 and CP000641–3), *Shigella boydii* Sb227 (CP000036–7), *Shigella dysenteriae* (CP000034–5 and CP000640), *Shigella flexneri* 2a 2457T (AE014073) and *S. flexneri* 5 8401 (CP000266). Also included were the unfinished genomes of *E. coli* strains 101-1 (RefSeq accession NZ_AAMK00000000), F11 (NZ_AAJU00000000), E22 (NZ_AAJV00000000), B171 (NZ_AAJX00000000), B7A (NZ_AAJT00000000) and E110019 (NZ_AAJW00000000). Genome sequences are also available from additional strains of *E. coli* K-12, O157:H7 and *S. flexneri* 2a, but these were omitted from the analysis due to their similarity to strains *E. coli* MG1655, EDL933 and 2457T, respectively.

The protein sequences predicted to be encoded by every annotated CDS from each of the chosen genomes were used as the query sequences in blastp searches of the total protein set, to assess the presence/absence of homologous proteins encoded by the other genomes. A homologue was considered to be present if a hit was found with >60% identity over at least 80% of the length of the query protein. To overcome potential problems associated with variations in annotation between the different genomes, the predicted protein sequences were also used as the query sequences in tblastn searches against the total set of genome sequences. If a homologue was identified in any of the genomes in the tblastn search that was not identified in the blastp search, using the same criteria as above, then an unannotated gene potentially encoding a homologous protein was considered to be present. The size of the core genome and pangenome were assessed using methods similar to those previously described [22], [108].

As the *E. coli* K-12 MG1655 genome is thought to be the most comprehensively annotated strain, 2370 annotated genes from that genome which were conserved in all the other strains were used to represent the *E. coli* core genome (listed in Table S2). These genes were used as the query sequences in further tblastn searches against a database consisting of complete and almost complete non-*E. coli* enterobacterial genomes. The included genomes were from *Escherichia albertii*; *Escherichia fergusonii*; *Citrobacter koseri*; *S. enterica* serovars Typhi, Typhimurium and Gallinarum; *Yersinia pestis* strains KIM, CO92, Angola, Antiqua, Nepal516 and Pestoides F; *Yersinia pseudotuberculosis* strains IP 31758, IP 32953, PB1/+ and YPIII; *Yersinia enterocolitica*; *Serratia proteamaculans*; *Proteus mirabilis*; *Photorhabdus luminescens*; *Klebsiella pneumoniae*; *Erwinia tasmaniensis*; *Enterobacter* sp. 638; *Enterobacter sakazakii* and *Pectobacterium atrosepticum*. Organisms from the genera *Sodalis*, *Wigglesworthia*, *Buchnera* and *Blochmannia* were omitted from this analysis since they are endosymbionts with reduced genomes that are not representative of the genomes of free-living enterobacteria. The same criteria of >60% identity over at least 80% of the length of the query sequence were used to determine the presence or absence of homologues.

### Phylogenetic Relationships within *E. coli*

To investigate the phylogenetic relationships among the *E. coli* genomes we selected a set of 2173 *E. coli* K-12 genes that were conserved in all the other *E. coli* genomes and in *E. albertii* and *E. fergusonii*, which were included as an outgroup. The homologous sequences from our representative set of *E. coli* genomes were compiled and aligned using ClustalW [109]. A maximum likelihood phylogeny was obtained using the general time reversible (GTR/REV) model with the CAT approximation of rate heterogeneity as implemented in RAxML version 7.0.4 [110]. Support for individual branches was assessed by conducting 100 non-parametric bootstrap replicates, using the rapid algorithm implemented in RAxML [111]. The phylogeny was displayed using MEGA version 4 [112].

#### Molecular biology techniques and functional assays

PCR reactions were performed using BioLine Readymix according to manufacturer's instructions. Primers for amplification of Ec042-2242, 4511 and 4803 alleles possessed the following sequences 5′ CTGAGCTCCGTGAACAGTTTACCGGTGC-3′ (forward primer), 5′-CAGAAGGTCCCGGCCACACCCCCGTTTTTGACA-3′ (2242), 5′-GGCCGGGACCTTCTGACAGAACCATCGCCTCTC-3′ (4511) and 5′-TTTCTAGATCATCAGGTGTGAATGACAGG-3′ (4803). All reactions were performed with a 60°C annealing temperature. Reaction products were analysed by DNA agarose electrophoresis as previously described. Products were cloned into pCRII-TOPO (Invitrogen) according to manufacturers' instructions. Proteins were analysed by SDS-PAGE and Western immunoblotting as previously described [113].

The standard autoaggregation assay was performed as previously described; overnight cultures were allowed to stand and at various time points samples were removed from the top of the culture medium and the OD_600_ was measured [113]. Autoaggregation is noted as a decrease in the OD_600_ value. A biofilm assay was performed essentially as previously described; bacteria were grown overnight in 2 ml of LB medium in 15 ml polystyrene tubes before staining with crystal violet to visualise the biofilm [113].

Based on the sequence and phenotypic PM data the susceptibility of strains to a range of compounds was determined using the agar dilution method following guidelines of the British Society for Antimicrobial Chemotherapy [114]. To determine whether any of the changes in antimicrobial susceptibility seen between strains were due to altered membrane permeability/active efflux of antimicrobials the accumulation of the fluorescent dye Hoescht 33342 (bis-benzimide), a substrate of the major AcrAB-TolC efflux system was measured. Experiments were repeated at least four times in the presence and absence of the efflux pump inhibitor phenyl-arginine-β-naphthylamide (PAβN) or CCCP as previously described [115].

### Phenotype Microarrays

Phenotype microarray growth and respiration analyses were performed as described previously by Biolog Inc. (Hayward, California USA) [116], [117]. Colonies from the test strains (*E. coli* 042 and *E. coli* K-12 MG1655) were inoculated from pregrowth R2A or LB agar plates [116] into Biolog inoculating fluid and then grown in Biolog defined minimal medium or nitrogen-, sulphur- or phosphorous-free versions of the medium in twenty PM plates, which tested nearly 2000 phenotypes. Growth at 36°C and respiration was measured every 15 minutes for 24 hours as colour changes using an Omnilog reader. Tests were performed in duplicate, and the mean signal in arbitrary units calculated for each replicate before the arithmetic difference of the mean EAEC 042 signal minus the mean of the *E. coli* MG1655 signal for each test well was calculated.

## Supporting Information

Table S1 Regions of difference (RODS) in the EAEC 042 genome.
(0.04 MB XLS)

Table S2 List of genes conserved in all sequenced *E. coli* genomes.
(1.99 MB DOC)

Table S3 Comparison of EAEC 042 and *E. coli* MG1655 metabolism by phenotype microarray, where EAEC 042 shows greater metabolic activity.
(0.12 MB DOC)

Table S4 Comparison of EAEC 042 and *E. coli* MG1655 metabolism by phenotype microarray, where *E. coli* MG1655 shows greater metabolic activity.
(0.07 MB DOC)

Table S5 Comparison of iron transport / storage genes from MG1655, O157 (Sakai) and O42.
(0.05 MB XLS)

Table S6 List of EAEC 042 CDS conserved in the other sequenced EAEC genomes (101-1 and 55989), but absent from the genome of the commensal *E. coli* HS.
(0.20 MB DOC)

Table S7 Type 3 secretion system effector genes in the EAEC 042 genome.
(0.05 MB DOC)

Figure S1 Genetic similarity of the EAEC 042 prophage. Nine prophage regions, designated 042p1–042p9, were identified in the EAEC 042 genome (see Table 2 in manuscript). The figure represents homology of the phage elements; solid lines indicate complete identity, the absence of lines within the boxes reflect little or no homology and the presence of dashed lines indicates partial identity. Four of these were lambdoid in nature (042p2, 042p3, 042p4 and 042p6) and were highly similar to each other and to the lambdoid prophages of *E. coli* O157:H7 [1], and *E. coli* O127:H6 strain E2348/69 (EPEC) [2]. Some of the related lambdoid prophages in EAEC 042 and *E. coli* O157:H7 are integrated in corresponding genomic locations and share some sequence identity; 042p3 and Sp10, 042p4 and Sp11/Sp12, and 042p6 and Sp14 are found relative to each other in the respective bacterial genomes. The EAEC 042 lambda-like prophages also have high homology to lambdoid prophages in several of the other sequenced pathogenic *E. coli* genomes including avaian pathogenic (APEC), uropathogenic (UPEC) and enterotoxigenic (ETEC). In addition, 042p4 has high homology to the K-12 cryptic prophage Qin and genome comparison shows where the deletions have occurred in the Qin genome to render it defective. 042p5 and 042p7 have some sequence similarity to O157:H7 Sakai prophage Sp7 which is of an unstudied type [1], [3]. Half of prophage 042p1, from the non-tail end to the lysis module, is related to lambda, P22 and PP8 in EPEC, but the remainder of the prophage, which encodes mainly structural proteins, is highly related to the *Shigella flexneri* serotype converting phage SfV [4]. 042p1 encodes bactoprenol glucosyl transferase (GtrB) and glucose translocase (GtrA), at the very end of the prophage, after the tail genes. These proteins are involved in O-antigen modification and have homology to the serotype-converting proteins of SfV (87% protein identity to GtrA and GtrB) and P22 (88% protein identity to GtrA and 77% to GtrB) [4], [5]. It is therefore likely that the presence of 042p1 in the genome of EAEC 042 alters the serotype of the host bacterium. 042p3 encodes an iron ABC transport system SitABCD which is carried at the tail end of the prophage and is discussed later. The only other EAEC 042 prophage for which we have identified putative cargo is 042p6 which encodes a putative exodeoxyribonuclease VIII. 042p9 is a P4-like satellite phage, and although there is no P2-like phage in the EAEC 042 genome for it to parasitise, it is possible that 042p9 is able to induce virion morphogenesis in a different helper phage [6]. 042p8 has no sequence similarity to any currently known phages and its only identified homologue is ROD-9 in ETEC H10407. Five of the EAEC 042 prophages (042p1, 042p2, 042p3, 042p4, and 042p6) appear to have all the necessary genes to produce fully functional phages. The other prophage regions, with the exception of the satellite prophage 042p9, are probably non-functional prophage-like remnants.
(1.20 MB DOC)

Figure S2 Genetic map of pAA, the large virulence plasmid of EAEC 042. (A) On the basis of nucleotide sequence homology, the plasmid pAA belongs to the IncFIIA family and carries just one identifiable replicon consisting of Ec042-pAA152 (RepA) and Ec042-pAA153 (CopB), which is in contrast to many of the F-family plasmids that have multiple replicons. It possesses auxiliary stable inheritance functions including Ec042-pAA136–137 encoding the type 2 partitioning proteins ParM and ParR, a Hok/Sok post-segregational killing system (Ec042-pAA106) and the putative pair of Ec042-pAA147–148 encoding a RelE/StbE homologue. As for most IncFII plasmids, pAA appears to encode a complete F-like conjugative transfer system (Ec042-pAA066–100), most closely related to those of pUT189 and R100, and does not seem to lack any standard component which might explain why attempts to transfer a derivative tagged with an antibiotic resistance marker (IH, unpublished) have been unsuccessful. However, analysis of the predicted gene products identified two CDS with unusual features that might be worth investigating as the basis of a transfer defect: TraP (Ec042-pAA092) shows N-terminal segments differing significantly from the nearest relatives despite high overall sequence alignment; and TrwB (Ec042-pAA069) contains an internal region towards the end of the protein with a significant amplification of a run of PQQP repeats which may have caused it to become non-functional. Functional analyses are needed to determine whether these features could be responsible for the Tra- phenotype. Other transfer-associated genes are: Ec042-pAA129 encoding a putative anti-restriction gene; Ec042-pAA113 encoding Ssb (single stranded DNA binding protein); Ec042-pAA109–110 encoding the SOS-induced response proteins PsiA/B associated with plasmid transfer; and Ec042-pAA101 encoding a lytic transglycosylase that helps hydrolyse cell wall in recipients prior to conjugative transfer. With respect to overall organisation it is interesting that the point at which a large amount of mobile DNA has been inserted into the plasmid is between finO (Ec042-pAA066) at the end of the transfer region and the rep region (Ec042-pAA0152–0153). In many, but not all, F-like plasmids these are contiguous and there is evidence that this region is involved in coordination between the transfer process and plasmid copy number/replication. However, in F there is an IS insertion in the finO gene itself, so there is precedent for disruption of this region. Plasmid pAA is relatively unusual in having most of the transposable elements inserted into this particular region. Thus, the plasmid has many standard elements but with features that render it worthy of further study.
(0.43 MB DOC)

Figure S3 Global comparison between EAEC 042 chromosome and those of EHEC, UPEC and K-12. ACT comparison (http://www.sanger.ac.uk/Software/ACT) of amino-acid matches between the complete six-frame translations (computed using TBLASTX) of the whole genome sequences of enterohaemorrhagic *E. coli* O157:H7 str. Sakai (EHEC; EMBL acc: BA000007), enterohaemorrhagic *E. coli* O157:H7 EDL933 (EHEC; EMBL acc: AE005174), uropathogenic *E. coli* strain CFT073 (UPEC; EMBL acc: AE014075), and *E. coli* strain K-12 MG1655 (K-12; EMBL acc: U00096). Forward and reverse strands of DNA are shown for each genome (dark grey lines). The red bars between the DNA lines represent individual TBLASTX matches, with inverted matches coloured blue.
(1.37 MB DOC)

Figure S4 Box-plot showing the estimated core genome size as a function of the number of genomes sequenced. The plot was constructed based on 100 randomly selected permutations of 30 genome sequences. The curve is approaching the limit suggesting that the 2356 conserved genes identified in this study represent the minimal *E. coli* genome and that this number is unlikely to reduce much further with additional *E. coli* genome sequencing.
(0.10 MB DOC)

Figure S5 The *E. coli* pan-genome. A. Box-plot showing the total size of the *E. coli* pan-genome as a function of the number of genomes sequenced. B. Box-plot showing the number of additional genes discovered with each genome sequenced. The plots were constructed using the same method as Fig. S4. Both indicate an open pan-genome, with ∼360 new genes being identified with each additional genome sequenced.
(0.16 MB DOC)

Figure S6 Phylogenetic relationships among sequenced *E. coli* and *Shigella* genomes. The genomes of *Escherichia albertii* and *Escherichia fergusonii* are included as outgroup sequences. The tree was obtained by maximum likelihood analysis of a concatenated alignment of 2173 genes, using the general time reversible (GTR/REV) model with the CAT approximation of rate heterogeneity as implemented in RAxML version 7.0.4. The numbers on individual branches indicate the percentage support from 100 non-parametric bootstrap replicates, performed using the rapid algorithm implemented in RAxML. The major *E. coli* phylogenetic groups are indicated.
(0.04 MB DOC)

Figure S7 Alignment of the ars operon from of *E. coli* K12 with EAEC 042. Alignments reveal genetic lesions in the *E. coli* K12 locus which result in the lack of the arsD and arsA genes. The loss of these genes confer upon *E. coli* K12 a reduced ability to grow in the presence of arsenite and antimony chloride.
(0.12 MB DOC)

Figure S8 Alignment of the EAEC 042 and *E. coli* MG1655 aga gene clusters. *E. coli* MG1655 lacks the agaE and agaF genes and has a truncated agaA gene. These truncations result in decreased N-acetyl-D-galactosamine and N-acetyl-D-glucosamine utilisation.
(0.22 MB DOC)

Figure S9 The sor operon of EAEC 042. EAEC 042 possesses the sor operon and has the ability to utilise sorbose as a sole carbon source. *E. coli* K-12 lacks the operon and can not utilise this carbon source, as confirmed by the BioLog phenotyping arrays.
(0.22 MB DOC)

Figure S10 Comparison of the genomic region from *E. coli* K-12 and EAEC 042 encoding garD. The EAEC 042 gene is disrupted whereas it is uninterrupted in *E. coli* K-12. Disruption of garD resulted in the inability of EAEC 042 to utilise D-galactarate as a sole carbon source. *E. coli* K12 can utilise this substrate.
(0.15 MB DOC)

Figure S11 Genetic architecture of the region encoding the als locus from *E. coli* K-12 and the similar region from EAEC 042. The als locus is absent from EAEC 042 explaining the inability of EAEC 042 to utilise allose as a sole carbon source; *E. coli* K12 is capable of using allose as a sole carbon source. This prediction was confirmed by BioLog PMs.
(0.20 MB DOC)

Figure S12 Sequence alignment of the *E. coli* K-12 and EAEC 042 porins. All the porins identified in EAEC 042 were aligned with those from *E. coli* K12 MG1655. NmpC (OmpD) is a pseudogene in *E. coli* K12 characterised by a C-terminal deletion. Identical residues are marked by asterisks, whereas similar residues are marked by periods and colons. The amino acid sequences of the OmpF, OmpC, PhoE and OmpN alleles from EAEC 042 and *E. coli* MG1655 are relatively well conserved (see Figure 4 in manuscript). In contrast, the OmpD proteins are more divergent and Ec042-2121 is sufficiently divergent to represent an apparently different lineage. Such divergence may be indicative of additional functions.
(0.22 MB DOC)

Figure S13 Genetic architecture and distribution of the Chaperone-Usher systems of EAEC 042. (A) The organisation of the fimbrial loci from EAEC 042 are depicted. CDS flanking the fimbrial loci are depicted by green arrows, CDS encoding regulators are depicted by purple arrows, CDS encoding chaperones are indicated with black arrows, ushers are depicted with red arrows and the secreted fimbrial subunits by blue arrows. CDS of unknown function are depicted by orange arrows. The EAEC CDS designation for the first flanking gene is indicated along with the common name for the loci. The loci designated 0022 and 3336 are uncharacterised fimbrial loci and the numbers represent the EAEC CDS designation for the gene encoding the putative usher protein. (B) Phylogenetic distribution of the fimbrial loci amongst pathogenic and non-pathogenic lineages of *E. coli*. The loci encoding the EAEC fimbrial systems demonstrate a differential distribution amongst the *E. coli* phylogeny but with some evidence of phylogenetic clustering.
(0.26 MB DOC)

Figure S14 Genetic architecture of the RTX-like Type 1 secretion locus. An alignment of the genomic regions encompassing the T1SS is depicted, demonstrating the absence of the locus in *E. coli* K12 and the presence of the locus in EAEC 042 and *E. coli* O157:H7. In both pathogenic strains the gene encoding the putative secreted RTX-like protein is frameshifted and would be predicted to encode a non-functional protein. The frameshifts occur at different positions within the respective genes; in EAEC 042 there are 3 frameshifts whereas in *E. coli* O157:H7 there is only one frameshift. The position of the OMP, ABC, MFP and a putative regulator are indicated.
(0.40 MB DOC)

Figure S15 Genomic architecture of the Type II secretion system (T2SS) apparatus of EAEC 042 and representative *E. coli* strains. The T2SS locus (gsp) appears intact in EAEC 042 and a variety of phylogenetically disparate strains of pathogenic *E. coli*. In contrast, the locus possesses a lesion in *E. coli* K12 and is absent in *E. coli* CFT073. Where the locus is intact it is always syntenic with yghJ a gene encoding a putative secreted lipoprotein.
(0.88 MB DOC)

Figure S16 Phylogenetic distribution of members of the Type 5 secretion system present in EAEC 042. The Autotransporters (AT-1) are differentially represented amongst the *E. coli* phylogeny, though several members of this group show phylogenetic clustering. The Two-partner secretion system (TPS) is present in all strains of *E. coli* suggesting an important physiological role. The trimeric Autotransporters (AT-2) are differentially represented amongst the *E. coli* phylogeny. Only upaG, a extracellular matrix binding protein, shows a wide distribution and is present in both commensal and pathogenic lineages suggesting the protein is not directly responsible for the ability of *E. coli* to mediate disease.
(0.18 MB DOC)

Figure S17 Comparison of three type VI secretion systems (T6SSs) of EAEC 042, and a separate Vgr-encoding region. Genes depicted by the schematic are indicated by the “Ec042” numbers on the right of the figure. Genes are designated by arrows, which are colored by xBASE to correspond to GC content, except for Hcp- and Vgr-homologs, which are shown in purple and white, respectively. Red blocks connecting genes indicate significant (P<0.05) identity on the amino acid level among homologs in all three T6SSs. Blue connectors indicate homologs that were only found within two T6SSs. The locus labeled Ec042-1584–1588 depicts a chromosomal region that encodes a Vgr-homolog, but is not in proximity to the other T6SSs. One of the T6SS identified (Ec042-4562–4577) had been previously characterized [7] as a locus under the control of AggR, the master regulator of virulence in this strain. The genes are part of a 117-kb pathogenicity island that is inserted at the pheU locus. This T6SS does not encode an Hcp homolog [7], however it is speculated that the third gene in this locus, previously designated aaiC, is a functional homolog. BLASTN results of this entire locus indicate that a nearly identical 18.4 kb sequence is found in enteroaggregative *E. coli* (EAEC) strain 55989 [8]. This is consistent with the previous report [7] that homologs of aaiC and the first gene of this locus designated aaiA are widely distributed among strains of EAEC. Unlike Hcp from other organisms, which is found widely within T6SSs [9], homologs of AaiC were only found within putative T6SSs of the atypical EAEC strain 101-1 (33% amino acid identity; GenBank AAMK02000010) and Citrobacter youngae ATCC 29220 (30% amino acid identity; GenBank EEK20055). Therefore, the function of this T6SS may be specific to the pathogenesis of EAEC and a limited number of other pathogens.
(0.21 MB DOC)

Figure S18 Genetic architecture of the capsular polysaccharide locus from EAEC 042. The locus is required for the biosynthesis of a group 2 capsular polysaccharide and appears to contain all the functional genes necessary for capsular production. Three CDS are present in the central region 2 and are likely to be important in the biosynthesis of the particular capsular polysaccharide expressed by this strain of *E. coli*. The ORF Ec042-3238 had 33% identity and 53% homology over 202 amino acids to CMP-N-acetylneuraminic acid synthetase enzymes from a number of bacterial species. This enzyme catalyses the conversion of CTP and neuraminic (NeuNAc) acid to form CMP-NeuNAc a key sugar activation step for the subsequent incorporation of NeuNAc into polysaccharides. At this stage in the absence of biochemical data one cannot be unequivocal about the enzymatic activity of the encoded protein but one can be sure it is involved in the activation of a nine-carbon sugar, which may be NeuNAc. Gene Ec042-3237 is predicted to encode a large protein of 1113 amino acids and has significant homology to the HAD super family of hydrolase enzymes and a number of putative glycosyl transferases from *Salmonella enterica*. This analysis would suggest a role in polysaccharide biosynthesis but in the absence of any structural data for the capsular polysaccharide expressed by this strain assigning a precise function to this protein is difficult. The remaining ORF in region 2 Ec042-3236 has significant homology (50% over 206 amino acids) with a putative acetyl-transfersae from *Neisseria meningitidis* and less homology to similar acetyl-transferases from a number of other bacteria. One possibility is that this is an acetyl-transferase that acetylates the capsular polysaccharide of this strain. Acetylation of the K1 polysaccharide of *E. coli* is known to occur and has been suggested as a mechanism by which the antigenicity of the cell surface polysaccharide may be modified in a stochastic fashion [10]. Analysis of the sequences 800 bp 5′ to Ec042-3230 showed no differences to those sequences 5′ to group 2 capsule gene clusters in strains of *E. coli* known to encode group 2 capsules such as UT189, CFT073 and APEC 01. This conservation of the promoter and regulatory region would indicate that regulation of expression of this capsule gene cluster in Ec042 involves SlyA, H-NS, IHF and BipA as previously demonstrated for other group 2 capsule gene clusters [11]. Implicit in this is that the expression will be temperature regulated being on at 37°C but not at 20°C [12]. A similar analysis of the sequences 5′ to Ec042-3240 showed no differences to those sequences 5′ to KpsM in other group 2 capsule gene clusters. The ops site 28 bp 5′ to kpsM, which is the cis-acting site for RfaH and is essential to allow read through transcription from the region 3 promoter into region 2 [13] was also conserved 28 bp 5′ to Ec042-3240. This conservation of the region 3 promoter and regulatory sequences would indicate that this group 2 capsule gene cluster is functional. The gene 5′ to region 3 in Ec042 is the gene encoding for the putative general secretion pathway protein YghD. This is the same organization as with other group 2 capsule gene clusters. Likewise the sequence 5′ to Ec042-3230 including the ORF Ec042-3229 also flank group 2 capsule gene clusters indicating these flanking regions have been acquired with the group 2 capsule gene cluster.
(0.11 MB DOC)

Figure S19 Comparison of the EAEC 042 chromosomal- and plasmid-based copies of the shf loci. The sequences surrounding the loci are not homologous. The ca. 3 kb of nucleotide sequence encompassing shf, rfbU and virK is identical; msbB2 is absent from the plasmid copy but present in the chromosomal copy. The similarity of Shf to the *Staphylococcus epidermidis* protein IcaB, which is required for exopolysaccharide modification and biofilm formation, indicated that Shf might also play a role in polysaccharide modification, a hypothesis supported by the presence of a polysaccharide deacetylase domain within Shf [14]. Recently, RfbU was discovered to be responsible for the addition of α-1,7-GlcN to the R3 core region of LPS and has been renamed WabB [15]. The R3 core is found in a variety of pathogenic *E. coli* and *Shigella* and is of significant biomedical interest. The function of VirK remains elusive. Initially described in *Shigella*, where it was found to be necessary for localisation of the autotransporter IcsA to the bacterial surface and subsequent intracellular spreading [16], VirK has been characterised in *Salmonella* where it has also been shown to be important for virulence [17]. MsbB2 was recently shown to act as a myristoyl transferase which modifies the lipid A portion of LPS and acts in a manner analogous to the chromosomal gene lpxM [18]. Furthermore, several investigations have demonstrated the importance of this gene for full virulence in *Shigella* and *E. coli*. Deletion of both msbB2 and lpxM results in altered membrane fatty acid composition and susceptibility to a variety of antibiotics and detergents suggesting defects in membrane biogenesis [19].
(0.15 MB DOC)

File S1 References for supplementary data.
(0.04 MB DOC)

## References

[1] Chang DE, Smalley DJ, Tucker DL, Leatham MP, Norris WE (2004). Carbon nutrition of *Escherichia coli* in the mouse intestine.. Proc Natl Acad Sci U S A.

[2] Kaper JB, Nataro JP, Mobley HL (2004). Pathogenic *Escherichia coli*.. Nat Rev Microbiol.

[3] Iguchi A, Thomson NR, Ogura Y, Saunders D, Ooka T (2009). Complete genome sequence and comparative genome analysis of enteropathogenic *Escherichia coli* O127:H6 strain E2348/69.. J Bacteriol.

[4] Nataro JP, Kaper JB (1998). Diarrheagenic *Escherichia coli*.. Clin Microbiol Rev.

[5] Nataro JP, Kaper JB, Robins-Browne R, Prado V, Vial P (1987). Patterns of adherence of diarrheagenic *Escherichia coli* to HEp-2 cells.. Pediatr Infect Dis J.

[6] Harrington SM, Dudley EG, Nataro JP (2006). Pathogenesis of enteroaggregative *Escherichia coli* infection.. FEMS Microbiol Lett.

[7] Nataro JP, Deng Y, Cookson S, Cravioto A, Savarino SJ (1995). Heterogeneity of enteroaggregative *Escherichia coli* virulence demonstrated in volunteers.. J Infect Dis.

[8] Wilson A, Evans J, Chart H, Cheasty T, Wheeler JG (2001). Characterisation of strains of enteroaggregative *Escherichia coli* isolated during the infectious intestinal disease study in England.. Eur J Epidemiol.

[9] Nataro JP, Mai V, Johnson J, Blackwelder WC, Heimer R (2006). Diarrheagenic *Escherichia coli* infection in Baltimore, Maryland, and New Haven, Connecticut.. Clin Infect Dis.

[10] Huang DB, Nataro JP, DuPont HL, Kamat PP, Mhatre AD (2006). Enteroaggregative *Escherichia coli* is a cause of acute diarrheal illness: a meta-analysis.. Clin Infect Dis.

[11] Harrington SM, Strauman MC, Abe CM, Nataro JP (2005). Aggregative adherence fimbriae contribute to the inflammatory response of epithelial cells infected with enteroaggregative *Escherichia coli*.. Cell Microbiol.

[12] Nataro JP (2005). Enteroaggregative *Escherichia coli* pathogenesis.. Curr Opin Gastroenterol.

[13] Steiner TS, Nataro JP, Poteet-Smith CE, Smith JA, Guerrant RL (2000). Enteroaggregative *Escherichia coli* expresses a novel flagellin that causes IL-8 release from intestinal epithelial cells.. J Clin Invest.

[14] Steiner TS, Lima AA, Nataro JP, Guerrant RL (1998). Enteroaggregative *Escherichia coli* produce intestinal inflammation and growth impairment and cause interleukin-8 release from intestinal epithelial cells.. J Infect Dis.

[15] Henderson IR, Hicks S, Navarro-Garcia F, Elias WP, Philips AD (1999). Involvement of the enteroaggregative *Escherichia coli* plasmid-encoded toxin in causing human intestinal damage.. Infect Immun.

[16] Sheikh J, Czeczulin JR, Harrington S, Hicks S, Henderson IR (2002). A novel dispersin protein in enteroaggregative *Escherichia coli*.. J Clin Invest.

[17] Wanke CA, Mayer H, Weber R, Zbinden R, Watson DA (1998). Enteroaggregative *Escherichia coli* as a potential cause of diarrheal disease in adults infected with human immunodeficiency virus.. J Infect Dis.

[18] Eslava C, Navarro-Garcia F, Czeczulin JR, Henderson IR, Cravioto A (1998). Pet, an autotransporter enterotoxin from enteroaggregative *Escherichia coli*.. Infect Immun.

[19] Henderson IR, Czeczulin J, Eslava C, Noriega F, Nataro JP (1999). Characterization of pic, a secreted protease of Shigella flexneri and enteroaggregative *Escherichia coli*.. Infect Immun.

[20] Dudley EG, Thomson NR, Parkhill J, Morin NP, Nataro JP (2006). Proteomic and microarray characterization of the AggR regulon identifies a *pheU* pathogenicity island in enteroaggregative *Escherichia coli*.. Mol Microbiol.

[21] Kroenker R (2008). quantreg: Quantile Regression.. R package version 4.24.

[22] Rasko DA, Rosovitz MJ, Myers GS, Mongodin EF, Fricke WF (2008). The pangenome structure of *Escherichia coli*: comparative genomic analysis of *E. coli* commensal and pathogenic isolates.. J Bacteriol.

[23] Touchon M, Hoede C, Tenaillon O, Barbe V, Baeriswyl S (2009). Organised genome dynamics in the *Escherichia coli* species results in highly diverse adaptive paths.. PLoS Genet.

[24] Chen SL, Hung CS, Xu J, Reigstad CS, Magrini V (2006). Identification of genes subject to positive selection in uropathogenic strains of *Escherichia coli*: A comparative genomics approach.. Proc Natl Acad Sci U S A.

[25] Jaureguy F, Landreau L, Passet V, Diancourt L, Frapy E (2008). Phylogenetic and genomic diversity of human bacteremic *Escherichia coli* strains.. BMC Genomics.

[26] Hobman JL, Penn CW, Pallen MJ (2007). Laboratory strains of *Escherichia coli*: model citizens or deceitful delinquents growing old disgracefully?. Mol Microbiol.

[27] Yamamoto T, Echeverria P, Yokota T (1992). Drug resistance and adherence to human intestines of enteroaggregative *Escherichia coli*.. J Infect Dis.

[28] Mendez Arancibia E, Pitart C, Ruiz J, Marco F, Gascon J (2009). Evolution of antimicrobial resistance in enteroaggregative *Escherichia coli* and enterotoxigenic *Escherichia coli* causing traveller's diarrhoea.. J Antimicrob Chemother.

[29] Vila J, Vargas M, Casals C, Urassa H, Mshinda H (1999). Antimicrobial resistance of diarrheagenic *Escherichia coli* isolated from children under the age of 5 years from Ifakara, Tanzania.. Antimicrob Agents Chemother.

[30] Sang WK, Oundo JO, Mwituria JK, Waiyaki PG, Yoh M (1997). Multidrug-resistant enteroaggregative *Escherichia coli* associated with persistent diarrhea in Kenyan children.. Emerg Infect Dis.

[31] Vila J, Vargas M, Ruiz J, Espasa M, Pujol M (2001). Susceptibility patterns of enteroaggregative *Escherichia coli* associated with traveller's diarrhoea: emergence of quinolone resistance.. J Med Microbiol.

[32] Liebert CA, Hall RM, Summers AO (1999). Transposon Tn21, flagship of the floating genome.. Microbiol Mol Biol Rev.

[33] Dey S, Rosen BP (1995). Dual mode of energy coupling by the oxyanion-translocating ArsB protein.. J Bacteriol.

[34] Piddock LJ (2006). Multidrug-resistance efflux pumps - not just for resistance.. Nat Rev Microbiol.

[35] Andrews SC, Robinson AK, Rodriguez-Quinones F (2003). Bacterial iron homeostasis.. FEMS Microbiol Rev.

[36] Hu J, Kan B, Liu ZH, Yu SY (2005). Enteroaggregative Escherichia coli isolated from Chinese diarrhea patients with high-pathogenicity island of Yersinia is involved in synthesis of siderophore yersiniabactin.. World J Gastroenterol.

[37] Grosse C, Scherer J, Koch D, Otto M, Taudte N (2006). A new ferrous iron-uptake transporter, EfeU (YcdN), from *Escherichia coli*.. Mol Microbiol.

[38] Valko M, Morris H, Cronin MT (2005). Metals, toxicity and oxidative stress.. Curr Med Chem.

[39] Kohanski MA, Dwyer DJ, Hayete B, Lawrence CA, Collins JJ (2007). A common mechanism of cellular death induced by bactericidal antibiotics.. Cell.

[40] Christensen M, Borza T, Dandanell G, Gilles AM, Barzu O (2003). Regulation of expression of the 2-deoxy-D-ribose utilization regulon, deoQKPX, from Salmonella enterica serovar typhimurium.. J Bacteriol.

[41] Ray WK, Larson TJ (2004). Application of AgaR repressor and dominant repressor variants for verification of a gene cluster involved in N-acetylgalactosamine metabolism in *Escherichia coli* K-12.. Mol Microbiol.

[42] Brinkkotter A, Kloss H, Alpert C, Lengeler JW (2000). Pathways for the utilization of N-acetyl-galactosamine and galactosamine in *Escherichia coli*.. Mol Microbiol.

[43] Harrington SM, Sheikh J, Henderson IR, Ruiz-Perez F, Cohen PS (2009). The Pic protease of enteroaggregative *Escherichia coli* promotes intestinal colonization and growth in the presence of mucin.. Infect Immun.

[44] Lehmacher A, Bockemuhl J (2007). L-Sorbose utilization by virulent *Escherichia coli* and Shigella: different metabolic adaptation of pathotypes.. Int J Med Microbiol.

[45] Woodward MJ, Charles HP (1982). Genes for l-sorbose utilization in *Escherichia coli*.. J Gen Microbiol.

[46] Moritz RL, Welch RA (2006). The *Escherichia coli* argW-dsdCXA genetic island is highly variable, and *E. coli* K1 strains commonly possess two copies of dsdCXA.. J Clin Microbiol.

[47] Monterrubio R, Baldoma L, Obradors N, Aguilar J, Badia J (2000). A common regulator for the operons encoding the enzymes involved in D-galactarate, D-glucarate, and D-glycerate utilization in *Escherichia coli*.. J Bacteriol.

[48] Hubbard BK, Koch M, Palmer DR, Babbitt PC, Gerlt JA (1998). Evolution of enzymatic activities in the enolase superfamily: characterization of the (D)-glucarate/galactarate catabolic pathway in *Escherichia coli*.. Biochemistry.

[49] Kim C, Song S, Park C (1997). The D-allose operon of *Escherichia coli* K-12.. J Bacteriol.

[50] Poulsen TS, Chang YY, Hove-Jensen B (1999). D-Allose catabolism of *Escherichia coli*: involvement of *alsI* and regulation of *als* regulon expression by allose and ribose.. J Bacteriol.

[51] Hasona A, Kim Y, Healy FG, Ingram LO, Shanmugam KT (2004). Pyruvate formate lyase and acetate kinase are essential for anaerobic growth of *Escherichia coli* on xylose.. J Bacteriol.

[52] Nikaido H (2003). Molecular basis of bacterial outer membrane permeability revisited.. Microbiol Mol Biol Rev.

[53] Delcour AH (2009). Outer membrane permeability and antibiotic resistance.. Biochim Biophys Acta.

[54] Nikaido H, Rosenberg EY (1983). Porin channels in *Escherichia coli*: studies with liposomes reconstituted from purified proteins.. J Bacteriol.

[55] Thanassi DG, Cheng LW, Nikaido H (1997). Active efflux of bile salts by *Escherichia coli*.. J Bacteriol.

[56] Harder KJ, Nikaido H, Matsuhashi M (1981). Mutants of *Escherichia coli* that are resistant to certain beta-lactam compounds lack the ompF porin.. Antimicrob Agents Chemother.

[57] Gil-Cruz C, Bobat S, Marshall JL, Kingsley RA, Ross EA (2009). The porin OmpD from nontyphoidal Salmonella is a key target for a protective B1b cell antibody response.. Proc Natl Acad Sci U S A.

[58] Henderson IR, Navarro-Garcia F, Desvaux M, Fernandez RC, Ala'Aldeen D (2004). Type V protein secretion pathway: the autotransporter story.. Microbiol Mol Biol Rev.

[59] Czeczulin JR, Whittam TS, Henderson IR, Navarro-Garcia F, Nataro JP (1999). Phylogenetic analysis of enteroaggregative and diffusely adherent *Escherichia coli*.. Infect Immun.

[60] Berks BC, Palmer T, Sargent F (2005). Protein targeting by the bacterial twin-arginine translocation (Tat) pathway.. Curr Opin Microbiol.

[61] Humphries A, Deridder S, Baumler AJ (2005). *Salmonella enterica* serotype Typhimurium fimbrial proteins serve as antigens during infection of mice.. Infect Immun.

[62] Jouve M, Garcia MI, Courcoux P, Labigne A, Gounon P (1997). Adhesion to and invasion of HeLa cells by pathogenic *Escherichia coli* carrying the afa-3 gene cluster are mediated by the AfaE and AfaD proteins, respectively.. Infect Immun.

[63] Rodriguez E, Gaggero C, Lavina M (1999). The structural gene for microcin H47 encodes a peptide precursor with antibiotic activity.. Antimicrob Agents Chemother.

[64] Yang J, Baldi DL, Tauschek M, Strugnell RA, Robins-Browne RM (2007). Transcriptional regulation of the *yghJ-pppA-yghG-gspCDEFGHIJKLM* cluster, encoding the type II secretion pathway in enterotoxigenic *Escherichia coli*.. J Bacteriol.

[65] Tauschek M, Gorrell RJ, Strugnell RA, Robins-Browne RM (2002). Identification of a protein secretory pathway for the secretion of heat-labile enterotoxin by an enterotoxigenic strain of *Escherichia coli*.. Proc Natl Acad Sci U S A.

[66] Parsot C, Taxman E, Mekalanos JJ (1991). ToxR regulates the production of lipoproteins and the expression of serum resistance in *Vibrio cholerae*.. Proc Natl Acad Sci U S A.

[67] Pugsley AP, Francetic O, Hardie K, Possot OM, Sauvonnet N (1997). Pullulanase: model protein substrate for the general secretory pathway of gram-negative bacteria.. Folia Microbiol (Praha).

[68] Pallen MJ, Gophna U (2007). Bacterial flagella and Type III secretion: case studies in the evolution of complexity.. Genome Dyn.

[69] Ren CP, Beatson SA, Parkhill J, Pallen MJ (2005). The Flag-2 locus, an ancestral gene cluster, is potentially associated with a novel flagellar system from *Escherichia coli*.. J Bacteriol.

[70] Ren CP, Chaudhuri RR, Fivian A, Bailey CM, Antonio M (2004). The ETT2 gene cluster, encoding a second type III secretion system from *Escherichia coli*, is present in the majority of strains but has undergone widespread mutational attrition.. J Bacteriol.

[71] Sheikh J, Dudley EG, Sui B, Tamboura B, Suleman A (2006). EilA, a HilA-like regulator in enteroaggregative *Escherichia coli*.. Mol Microbiol.

[72] Pallen MJ (2003). Glucoamylase-like domains in the alpha- and beta-subunits of phosphorylase kinase.. Protein Sci.

[73] Bodelon G, Marin E, Fernandez LA (2009). Role of periplasmic chaperones and BamA (YaeT/Omp85) in folding and secretion of intimin from enteropathogenic *Escherichia coli* strains.. J Bacteriol.

[74] Desvaux M, Parham NJ, Henderson IR (2004). Type V protein secretion: simplicity gone awry?. Curr Issues Mol Biol.

[75] Henderson IR, Nataro JP, Kaper JB, Meyer TF, Farrand SK (2000). Renaming protein secretion in the gram-negative bacteria.. Trends Microbiol.

[76] Parham NJ, Srinivasan U, Desvaux M, Foxman B, Marrs CF (2004). PicU, a second serine protease autotransporter of uropathogenic *Escherichia coli*.. FEMS Microbiol Lett.

[77] Wells TJ, Tree JJ, Ulett GC, Schembri MA (2007). Autotransporter proteins: novel targets at the bacterial cell surface.. FEMS Microbiol Lett.

[78] van der Woude MW, Henderson IR (2008). Regulation and function of Ag43 (flu).. Annu Rev Microbiol.

[79] Stegmeier JF, Gluck A, Sukumaran S, Mantele W, Andersen C (2007). Characterisation of YtfM, a second member of the Omp85 family in *Escherichia coli*.. Biol Chem.

[80] Knowles TJ, Scott-Tucker A, Overduin M, Henderson IR (2009). Membrane protein architects: the role of the BAM complex in outer membrane protein assembly.. Nat Rev Microbiol.

[81] Knowles TJ, Jeeves M, Bobat S, Dancea F, McClelland D (2008). Fold and function of polypeptide transport-associated domains responsible for delivering unfolded proteins to membranes.. Mol Microbiol.

[82] Yen MR, Peabody CR, Partovi SM, Zhai Y, Tseng YH (2002). Protein-translocating outer membrane porins of Gram-negative bacteria.. Biochim Biophys Acta.

[83] Akerley BJ, Rubin EJ, Novick VL, Amaya K, Judson N (2002). A genome-scale analysis for identification of genes required for growth or survival of *Haemophilus influenzae*.. Proc Natl Acad Sci U S A.

[84] Yao Y, Xie Y, Kim KS (2006). Genomic comparison of *Escherichia coli* K1 strains isolated from the cerebrospinal fluid of patients with meningitis.. Infect Immun.

[85] Burall LS, Harro JM, Li X, Lockatell CV, Himpsl SD (2004). *Proteus mirabilis* genes that contribute to pathogenesis of urinary tract infection: identification of 25 signature-tagged mutants attenuated at least 100-fold.. Infect Immun.

[86] Valle J, Mabbett AN, Ulett GC, Toledo-Arana A, Wecker K (2008). UpaG, a new member of the trimeric autotransporter family of adhesins in uropathogenic *Escherichia coli*.. J Bacteriol.

[87] Pukatzki S, McAuley SB, Miyata ST (2009). The type VI secretion system: translocation of effectors and effector-domains.. Current Opinion in Microbiology.

[88] Dudley EG, Thomson NR, Parkhill J, Morin NP, Nataro JP (2006). Proteomic and microarray characterization of the AggR regulon identifies a *pheU* pathogenicity island in enteroaggregative *Escherichia coli*.. Mol Microbiol.

[89] Touchon M, Hoede C, Tenaillon O, Barbe V, Baeriswyl S (2009). Organised genome dynamics in the *Escherichia coli* species results in highly diverse adaptive paths.. PLoS Genet.

[90] Brzuszkiewicz E, Brüggemann H, Liesegang H, Emmerth M, Ölschläger T (2006). How to become a uropathogen: Comparative genomic analysis of extraintestinal pathogenic *Escherichia coli* strains.. Proc Natl Acad Sci USA.

[91] Chen SL, Hung C-S, Xu J, Reigstad CS, Magrini V (2006). Identification of genes subject to positive selection in uropathogenic strains of *Escherichia coli*: A comparative genomics approach.. Proc Natl Acad Sci U S A.

[92] Johnson TJ, Kariyawasam S, Wannemuehler Y, Mangiamele P, Johnson SJ (2007). The genome sequence of avian pathogenic *Escherichia coli* strain O1:K1:H7 shares strong similarities with human extraintestinal pathogenic *E. coli* genomes.. J Bacteriol.

[93] Mougous J, Cuff M, Raunser S, Shen A, Zhou M (2006). A virulence locus of *Pseudomonas aeruginosa* encodes a protein secretion apparatus.. Science.

[94] Navarro-Garcia F, Canizalez-Roman A, Luna J, Sears C, Nataro JP (2001). Plasmid-encoded toxin of enteroaggregative *Escherichia coli* is internalized by epithelial cells.. Infect Immun.

[95] Villaseca JM, Navarro-Garcia F, Mendoza-Hernandez G, Nataro JP, Cravioto A (2000). Pet toxin from enteroaggregative *Escherichia coli* produces cellular damage associated with fodrin disruption.. Infect Immun.

[96] Savarino SJ, Fasano A, Watson J, Martin BM, Levine MM (1993). Enteroaggregative *Escherichia coli* heat-stable enterotoxin 1 represents another subfamily of *E. coli* heat-stable toxin.. Proc Natl Acad Sci U S A.

[97] Menard LP, Lussier JG, Lepine F, Paiva de Sousa C, Dubreuil JD (2004). Expression, purification, and biochemical characterization of enteroaggregative *Escherichia coli* heat-stable enterotoxin 1.. Protein Expr Purif.

[98] Mueller M, Grauschopf U, Maier T, Glockshuber R, Ban N (2009). The structure of a cytolytic alpha-helical toxin pore reveals its assembly mechanism.. Nature.

[99] Lithgow JK, Haider F, Roberts IS, Green J (2007). Alternate SlyA and H-NS nucleoprotein complexes control hlyE expression in *Escherichia coli* K-12.. Mol Microbiol.

[100] von Rhein C, Hunfeld KP, Ludwig A (2006). Serologic evidence for effective production of cytolysin A in Salmonella enterica serovars typhi and paratyphi A during human infection.. Infect Immun.

[101] von Rhein C, Bauer S, Simon V, Ludwig A (2008). Occurrence and characteristics of the cytolysin A gene in Shigella strains and other members of the family Enterobacteriaceae.. FEMS Microbiol Lett.

[102] Whitfield C, Roberts IS (1999). Structure, assembly and regulation of expression of capsules in *Escherichia coli*.. Mol Microbiol.

[103] Smith AN, Boulnois GJ, Roberts IS (1990). Molecular analysis of the *Escherichia coli* K5 *kps* locus: identification and characterization of an inner-membrane capsular polysaccharide transport system.. Mol Microbiol.

[104] Goldman SR, Tu Y, Goldberg MB (2008). Differential regulation by magnesium of the two MsbB paralogs of Shigella flexneri.. J Bacteriol.

[105] Fujiyama R, Nishi J, Imuta N, Tokuda K, Manago K (2008). The *shf* gene of a *Shigella flexneri* homologue on the virulent plasmid pAA2 of enteroaggregative *Escherichia coli* 042 is required for firm biofilm formation.. Curr Microbiol.

[106] Tobe T, Beatson SA, Taniguchi H, Abe H, Bailey CM (2006). An extensive repertoire of type III secretion effectors in *Escherichia coli* O157 and the role of lambdoid phages in their dissemination.. Proc Natl Acad Sci U S A.

[107] Chaudhuri RR, Loman NJ, Snyder LA, Bailey CM, Stekel DJ (2008). xBASE2: a comprehensive resource for comparative bacterial genomics.. Nucleic Acids Res.

[108] Tettelin H, Masignani V, Cieslewicz MJ, Donati C, Medini D (2005). Genome analysis of multiple pathogenic isolates of *Streptococcus agalactiae*: implications for the microbial “pan-genome”.. Proc Natl Acad Sci U S A.

[109] Thompson JD, Higgins DG, Gibson TJ (1994). CLUSTAL W: improving the sensitivity of progressive multiple sequence alignment through sequence weighting, position-specific gap penalties and weight matrix choice.. Nucleic Acids Res.

[110] Goldman N, Yang Z (1994). A codon-based model of nucleotide substitution for protein-coding DNA sequences.. Mol Biol Evol.

[111] Yang Z (2007). PAML 4: phylogenetic analysis by maximum likelihood.. Mol Biol Evol.

[112] Gascuel O (1997). BIONJ: an improved version of the NJ algorithm based on a simple model of sequence data.. Mol Biol Evol.

[113] de Luna MG, Scott-Tucker A, Desvaux M, Ferguson P, Morin NP (2008). The *Escherichia coli* biofilm-promoting protein Antigen 43 does not contribute to intestinal colonization.. FEMS Microbiol Lett.

[114] Andrews J (2001). Determination of minimum inhibitory concentrations.. J Antimicrob Chemother.

[115] Webber MA, Randall LP, Cooles S, Woodward MJ, Piddock LJ (2008). Triclosan resistance in *Salmonella enterica* serovar Typhimurium.. J Antimicrob Chemother.

[116] Bochner BR, Gadzinski P, Panomitros E (2001). Phenotype microarrays for high-throughput phenotypic testing and assay of gene function.. Genome Res.

[117] Zhou L, Lei XH, Bochner BR, Wanner BL (2003). Phenotype microarray analysis of *Escherichia coli* K-12 mutants with deletions of all two-component systems.. J Bacteriol.

