# Supplementary material for: Complete Genome Sequence and Comparative Metabolic Profiling of the Prototypical Enteroaggregative Escherichia coli Strain 042
Source: PLoS One. 2010 Jan 20;5(1):e8801. doi: 10.1371/journal.pone.0008801 (PMC2808357; doi:10.1371/journal.pone.0008801)
Supplement: Table S2 — List of genes conserved in all sequenced E. coli genomes. (1.99 MB DOC) [file pone.0008801.s002.doc]

**Table S2.** List of genes conserved in all sequenced *E. coli* genomes.

| **Gene^a^** | **GI** | **Product** |
| --- | --- | --- |
| thrA^b^ | 1786183 | fused aspartokinase I and homoserine dehydrogenase I |
| thrB | 1786184 | homoserine kinase |
| thrC | 1786185 | threonine synthase |
| yaaX^c^ | 1786186 | predicted protein |
| yaaA | 1786187 | conserved protein |
| talB | 1786189 | transaldolase B |
| mog | 1786190 | predicted molybdochelatase |
| yaaH | 1786191 | conserved inner membrane protein associated with acetate transport |
| yaaW | 1786193 | conserved protein |
| dnaK | 1786196 | chaperone Hsp70, co-chaperone with DnaJ |
| dnaJ | 1786197 | chaperone Hsp40, co-chaperone with DnaK |
| mokC | 1786200 | regulatory protein for HokC, overlaps CDS of hokC |
| hokC | 48994874 | toxic membrane protein, small |
| nhaA | 1786201 | sodium-proton antiporter |
| nhaR | 1786202 | DNA-binding transcriptional activator |
| ribF | 1786208 | bifunctional riboflavin kinase/FAD synthetase |
| lspA | 1786210 | prolipoprotein signal peptidase (signal peptidase II) |
| fkpB | 1786211 | FKBP-type peptidyl-prolyl cis-trans isomerase (rotamase) |
| ispH | 1786212 | 1-hydroxy-2-methyl-2-(E)-butenyl 4-diphosphate reductase, 4Fe-4S protein |
| rihC | 1786213 | ribonucleoside hydrolase 3 |
| dapB | 1786214 | dihydrodipicolinate reductase |
| carA | 1786215 | carbamoyl phosphate synthetase small subunit, glutamine amidotransferase |
| carB | 1786216 | carbamoyl-phosphate synthase large subunit |
| fixC | 1786227 | predicted oxidoreductase with FAD/NAD(P)-binding domain |
| fixX | 1786228 | predicted 4Fe-4S ferredoxin-type protein |
| yaaU | 1786229 | predicted transporter |
| kefF | 1786231 | flavoprotein subunit for the KefC potassium efflux system |
| kefC | 1786232 | potassium:proton antiporter |
| folA | 1786233 | dihydrofolate reductase |
| apaH | 1786234 | diadenosine tetraphosphatase |
| apaG | 1786235 | protein associated with Co2+ and Mg2+ efflux |
| rsmA | 1786236 | S-adenosylmethionine-6-N',N'-adenosyl (rRNA) dimethyltransferase |
| pdxA | 1786237 | 4-hydroxy-L-threonine phosphate dehydrogenase, NAD-dependent |
| surA | 1786238 | peptidyl-prolyl cis-trans isomerase (PPIase) |
| djlA | 1786241 | DnaJ-like protein, membrane anchored |
| rluA | 1786244 | pseudouridine synthase for 23S rRNA (position 746) and tRNAphe(position 32) |
| hepA | 1786245 | RNA polymerase-associated helicase protein (ATPase and RNA polymerase recycling factor) |
| araD | 1786247 | L-ribulose-5-phosphate 4-epimerase |
| araA | 1786248 | L-arabinose isomerase |
| araB | 1786249 | L-ribulokinase |
| araC | 1786251 | DNA-binding transcriptional dual regulator |
| yabI | 1786252 | conserved inner membrane protein |
| thiQ | 1786253 | thiamin transporter subunit |
| thiP | 1786254 | fused thiamin transporter subunits of ABC superfamily: membrane components |
| thiB | 1786255 | thiamin transporter subunit |
| sgrR | 1786256 | DNA-binding transcriptional regulator |
| leuD | 1786258 | 3-isopropylmalate isomerase subunit |
| leuC | 1786259 | 3-isopropylmalate isomerase subunit, dehydratase component |
| leuB | 87081683 | 3-isopropylmalate dehydrogenase |
| leuA | 1786261 | 2-isopropylmalate synthase |
| leuL | 1786263 | leu operon leader peptide |
| ilvI | 87081685 | acetolactate synthase III, large subunit |
| ilvH | 1786266 | acetolactate synthase III, thiamin-dependent, small subunit |
| fruR | 1786268 | DNA-binding transcriptional dual regulator |
| mraZ | 1786269 | conserved protein |
| mraW | 1786270 | S-adenosyl-dependent methyltransferase activity on membrane-located substrates |
| ftsL | 1786271 | membrane bound cell division protein at septum containing leucine zipper motif |
| murE | 1786273 | UDP-N-acetylmuramoyl-L-alanyl-D-glutamate:meso- diaminopimelate ligase |
| murF | 1786274 | UDP-N-acetylmuramoyl-tripeptide:D-alanyl-D- alanine ligase |
| mraY | 1786275 | phospho-N-acetylmuramoyl-pentapeptide transferase |
| murD | 1786276 | UDP-N-acetylmuramoyl-L-alanine:D-glutamate ligase |
| ftsW | 1786277 | integral membrane protein involved in stabilizing FstZ ring during cell division |
| murG | 1786278 | N-acetylglucosaminyl transferase |
| murC | 1786279 | UDP-N-acetylmuramate:L-alanine ligase |
| ddlB | 1786280 | D-alanine:D-alanine ligase |
| ftsQ | 1786281 | membrane anchored protein involved in growth of wall at septum |
| secM | 87081686 | regulator of secA translation |
| mutT | 1786288 | nucleoside triphosphate pyrophosphohydrolase, marked preference for dGTP |
| yacG | 1786290 | DNA gyrase inhibitor |
| yacF | 1786291 | conserved protein |
| coaE | 1786292 | dephospho-CoA kinase |
| guaC | 1786293 | GMP reductase |
| hofB | 1786296 | conserved protein with nucleoside triphosphate hydrolase domain |
| nadC | 1786299 | quinolinate phosphoribosyltransferase |
| ampD | 1786300 | N-acetyl-anhydromuranmyl-L-alanine amidase |
| aroP | 1786302 | aromatic amino acid transporter |
| pdhR | 1786303 | DNA-binding transcriptional dual regulator |
| aceF | 1786305 | pyruvate dehydrogenase, dihydrolipoyltransacetylase component E2 |
| lpd | 1786307 | lipoamide dehydrogenase, E3 component is part of three enzyme complexes |
| yacH | 1786308 | predicted protein |
| acnB | 2367097 | bifunctional aconitate hydratase 2/2-methylisocitrate dehydratase |
| speD | 1786311 | S-adenosylmethionine decarboxylase |
| speE | 1786312 | spermidine synthase (putrescine aminopropyltransferase) |
| yacC | 87081688 | predicted protein |
| cueO | 1786314 | multicopper oxidase (laccase) |
| hpt | 87081689 | hypoxanthine phosphoribosyltransferase |
| yadG | 1786319 | predicted transporter subunit: ATP-binding component of ABC superfamily |
| yadH | 1786320 | predicted transporter subunit: membrane component of ABC superfamily |
| yadI | 1786321 | predicted PTS Enzyme IIA |
| yadE | 1786322 | predicted polysaccharide deacetylase lipoprotein |
| panD | 1786323 | aspartate 1-decarboxylase |
| yadD | 1786324 | predicted transposase |
| panC | 1786325 | pantothenate synthetase |
| panB | 1786326 | 3-methyl-2-oxobutanoate hydroxymethyltransferase |
| folK | 1786335 | 2-amino-4-hydroxy-6-hydroxymethyldihyropteridine pyrophosphokinase |
| pcnB | 87081691 | poly(A) polymerase I |
| yadB | 145693092 | glutamyl-Q tRNA(Asp) synthetase |
| dksA | 1786338 | DNA-binding transcriptional regulator of rRNA transcription, DnaK suppressor protein |
| sfsA | 1786340 | predicted DNA-binding transcriptional regulator |
| ligT | 87081692 | 2'-5' RNA ligase |
| hrpB | 87081693 | predicted ATP-dependent helicase |
| mrcB | 1786343 | fused glycosyl transferase and transpeptidase |
| fhuA | 1786344 | ferrichrome outer membrane transporter |
| fhuC | 1786345 | iron-hydroxamate transporter subunit |
| fhuD | 1786346 | iron-hydroxamate transporter subunit |
| fhuB | 1786347 | fused iron-hydroxamate transporter subunits of ABC superfamily: membrane components |
| hemL | 1786349 | glutamate-1-semialdehyde aminotransferase (aminomutase) |
| clcA | 1786350 | chloride channel, voltage-gated |
| erpA | 1786351 | conserved protein |
| yadS | 1786352 | conserved inner membrane protein |
| btuF | 1786353 | vitamin B12 transporter subunit: periplasmic-binding component of ABC superfamily |
| mtn | 1786354 | 5'-methylthioadenosine/S-adenosylhomocysteine nucleosidase |
| degP | 1786356 | serine endoprotease (protease Do), membrane-associated |
| cdaR | 87081694 | DNA-binding transcriptional activator |
| yaeH | 1786359 | conserved protein |
| dapD | 1786362 | 2,3,4,5-tetrahydropyridine-2-carboxylate N-succinyltransferase |
| glnD | 1786363 | uridylyltransferase |
| map | 1786364 | methionine aminopeptidase |
| rpsB | 1786365 | 30S ribosomal subunit protein S2 |
| tsf | 1786366 | protein chain elongation factor EF-Ts |
| pyrH | 1786367 | uridylate kinase |
| frr | 1786368 | ribosome recycling factor |
| dxr | 1786369 | 1-deoxy-D-xylulose 5-phosphate reductoisomerase |
| ispU | 1786371 | undecaprenyl pyrophosphate synthase |
| cdsA | 87081696 | CDP-diglyceride synthase |
| skp | 1786375 | periplasmic chaperone |
| lpxD | 1786376 | UDP-3-O-(3-hydroxymyristoyl)-glucosamine N-acyltransferase |
| fabZ | 1786377 | (3R)-hydroxymyristol acyl carrier protein dehydratase |
| lpxA | 1786378 | UDP-N-acetylglucosamine acetyltransferase |
| lpxB | 1786379 | tetraacyldisaccharide-1-P synthase |
| rnhB | 1786380 | ribonuclease HII, degrades RNA of DNA-RNA hybrids |
| dnaE | 1786381 | DNA polymerase III alpha subunit |
| accA | 1786382 | acetyl-CoA carboxylase, carboxytransferase, alpha subunit |
| ldcC | 1786384 | lysine decarboxylase 2, constitutive |
| yaeR | 87081697 | predicted lyase |
| tilS | 1786386 | tRNA(Ile)-lysidine synthetase |
| rof | 87081698 | modulator of Rho-dependent transcription termination |
| yaeP | 48994876 | conserved protein |
| yaeQ | 1786388 | conserved protein |
| yaeJ | 1786389 | conserved protein |
| nlpE | 1786390 | lipoprotein involved with copper homeostasis and adhesion |
| yaeF | 87081699 | predicted lipoprotein |
| proS | 1786392 | prolyl-tRNA synthetase |
| yaeB | 1786393 | conserved protein |
| rcsF | 1786394 | predicted outer membrane protein, signal |
| metQ | 1786396 | DL-methionine transporter subunit |
| metI | 1786397 | DL-methionine transporter subunit |
| metN | 1786398 | DL-methionine transporter subunit |
| dkgB | 1786400 | 2,5-diketo-D-gluconate reductase B |
| yafC | 1786401 | predicted DNA-binding transcriptional regulator |
| yafD | 1786403 | conserved protein |
| yafE | 1786404 | predicted S-adenosyl-L-methionine-dependent methyltransferase |
| mltD | 1786405 | predicted membrane-bound lytic murein transglycosylase D |
| gloB | 1786406 | predicted hydroxyacylglutathione hydrolase |
| yafS | 87081700 | predicted S-adenosyl-L-methionine-dependent methyltransferase |
| rnhA | 1786408 | ribonuclease HI, degrades RNA of DNA-RNA hybrids |
| dnaQ | 1786409 | DNA polymerase III epsilon subunit |
| yafV | 1786412 | predicted C-N hydrolase family amidase, NAD(P)-binding |
| ivy | 1786413 | inhibitor of vertebrate C-lysozyme |
| lpcA | 1786416 | D-sedoheptulose 7-phosphate isomerase |
| yafJ | 1786417 | predicted amidotransfease |
| yafK | 1786418 | conserved protein |
| pepD | 1786432 | aminoacyl-histidine dipeptidase (peptidase D) |
| gpt | 1786433 | guanine-hypoxanthine phosphoribosyltransferase |
| frsA | 1786434 | hydrolase, binds to enzyme IIA(Glc) |
| crl | 1786435 | DNA-binding transcriptional regulator |
| phoE | 1786436 | outer membrane phosphoporin protein E |
| proB | 1786437 | gamma-glutamate kinase |
| proA | 1786438 | gamma-glutamylphosphate reductase |
| argF | 1786469 | CP4-6 prophage; ornithine carbamoyltransferase 2, chain F |
| tauA | 87081727 | taurine transporter subunit |
| tauB | 1786563 | taurine transporter subunit |
| tauC | 1786564 | taurine transporter subunit |
| tauD | 1786565 | taurine dioxygenase, 2-oxoglutarate-dependent |
| hemB | 87081728 | porphobilinogen synthase |
| ampH | 1786574 | beta-lactamase/D-alanine carboxypeptidase |
| sbmA | 1786575 | predicted transporter |
| yaiW | 1786576 | predicted DNA-binding transcriptional regulator |
| yaiY | 1786577 | predicted inner membrane protein |
| yaiZ | 87081730 | predicted inner membrane protein |
| ddlA | 1786579 | D-alanine-D-alanine ligase A |
| iraP | 1786581 | predicted protein |
| psiF | 87081731 | conserved protein |
| proC | 1786585 | pyrroline-5-carboxylate reductase, NAD(P)-binding |
| yaiI | 87081732 | conserved protein |
| aroL | 1786587 | shikimate kinase II |
| yaiA | 1786588 | predicted protein |
| aroM | 1786589 | conserved protein |
| yaiE | 1786590 | conserved protein |
| rdgC | 1786592 | DNA-binding protein, non-specific |
| mak | 87081733 | manno(fructo)kinase |
| sbcC | 1786597 | exonuclease, dsDNA, ATP-dependent |
| phoB | 1786599 | DNA-binding response regulator in two-component regulatory system with PhoR (or CreC) |
| phoR | 1786600 | sensory histidine kinase in two-component regulatory system with PhoB |
| brnQ | 1786601 | predicted branched chain amino acid transporter (LIV-II) |
| proY | 1786602 | predicted cryptic proline transporter |
| acpH | 1786605 | conserved protein |
| queA | 1786606 | S-adenosylmethionine:tRNA ribosyltransferase-isomerase |
| tgt | 1786607 | tRNA-guanine transglycosylase |
| yajC | 1786608 | SecYEG protein translocase auxillary subunit |
| secD | 1786609 | SecYEG protein translocase auxillary subunit |
| secF | 1786610 | SecYEG protein translocase auxillary subunit |
| yajD | 1786611 | conserved protein |
| tsx | 1786612 | nucleoside channel, receptor of phage T6 and colicin K |
| nrdR | 1786615 | conserved protein |
| ribD | 1786616 | fused diaminohydroxyphosphoribosylaminopyrimidine deaminase and 5-amino-6-(5-phosphoribosylamino) uracil reductase |
| ribE | 1786617 | riboflavin synthase beta chain |
| nusB | 1786618 | transcription antitermination protein |
| thiL | 1786619 | thiamin-monophosphate kinase |
| pgpA | 1786620 | phosphatidylglycerophosphatase A |
| yajO | 87081735 | 2-carboxybenzaldehyde reductase, function unknown |
| dxs | 1786622 | 1-deoxyxylulose-5-phosphate synthase, thiamine-requiring, FAD-requiring |
| ispA | 1786623 | geranyltranstransferase |
| xseB | 1786624 | exonuclease VII small subunit |
| thiI | 1786625 | sulfurtransferase required for thiamine and 4-thiouridine biosynthesis |
| yajL | 87081736 | conserved protein |
| panE | 1786627 | 2-dehydropantoate reductase, NADPH-specific |
| yajQ | 87081737 | predicted nucleotide binding protein |
| lon | 1786643 | DNA-binding ATP-dependent protease La |
| hupB | 1786644 | HU, DNA-binding transcriptional regulator, beta subunit |
| ppiD | 1786645 | peptidyl-prolyl cis-trans isomerase (rotamase D) |
| tesC | 1786647 | thioesterase III |
| queC | 1786648 | predicted aluminum resistance protein |
| ybaE | 1786650 | predicted transporter subunit: periplasmic-binding component of ABC superfamily |
| ybaO | 87081742 | predicted DNA-binding transcriptional regulator |
| mdlA | 1786653 | fused predicted multidrug transporter subunits of ABC superfamily: ATP-binding components |
| mdlB | 1786654 | fused predicted multidrug transporter subunits of ABC superfamily: ATP-binding components |
| glnK | 1786655 | nitrogen assimilation regulatory protein for GlnL, GlnE, and AmtB |
| amtB | 1786656 | ammonium transporter |
| tesB | 1786657 | acyl-CoA thioesterase II |
| ybaY | 1786658 | predicted outer membrane lipoprotein |
| ybaZ | 1786659 | predicted methyltransferase |
| ybaA | 1786661 | conserved protein |
| maa | 1786664 | maltose O-acetyltransferase |
| hha | 1786665 | modulator of gene expression, with H-NS |
| tomB | 1786666 | predicted protein |
| acrB | 1786667 | multidrug efflux system protein |
| acrA | 1786668 | multidrug efflux system |
| acrR | 1786669 | DNA-binding transcriptional repressor |
| kefA | 1786670 | fused conserved protein |
| ybaM | 1786672 | predicted protein |
| priC | 1786673 | primosomal replication protein N'' |
| ybaN | 1786674 | conserved inner membrane protein |
| apt | 1786675 | adenine phosphoribosyltransferase |
| ybaB | 1786677 | conserved protein |
| recR | 1786678 | gap repair protein |
| htpG | 1786679 | molecular chaperone HSP90 family |
| adk | 1786680 | adenylate kinase |
| hemH | 1786681 | ferrochelatase |
| aes | 1786682 | acetyl esterase |
| gsk | 1786684 | inosine/guanosine kinase |
| cueR | 1786695 | DNA-binding transcriptional activator of copper-responsive regulon genes |
| ybbJ | 145693099 | conserved inner membrane protein |
| qmcA | 1786697 | predicted protease, membrane anchored |
| ybbL | 1786698 | predicted transporter subunit: ATP-binding component of ABC superfamily |
| purK | 1786733 | N5-carboxyaminoimidazole ribonucleotide synthase |
| purE | 1786734 | N5-carboxyaminoimidazole ribonucleotide mutase |
| lpxH | 1786735 | UDP-2,3-diacylglucosamine pyrophosphatase |
| ppiB | 1786736 | peptidyl-prolyl cis-trans isomerase B (rotamase B) |
| cysS | 1786737 | cysteinyl-tRNA synthetase |
| ybcJ | 87081752 | predicted RNA-binding protein |
| folD | 1786741 | bifunctional 5,10-methylene-tetrahydrofolate dehydrogenase/ 5,10-methylene-tetrahydrofolate cyclohydrolase |
| essD | 1786767 | DLP12 prophage; predicted phage lysis protein |
| pheP | 1786789 | phenylalanine transporter |
| ybdG | 1786791 | predicted mechanosensitive channel |
| nfsB | 1786792 | dihydropteridine reductase, NAD(P)H-dependent, oxygen-insensitive |
| ybdF | 1786793 | conserved protein |
| ybdJ | 1786794 | predicted inner membrane protein |
| ybdK | 1786795 | gamma-glutamyl:cysteine ligase |
| hokE | 48994879 | toxic polypeptide, small |
| entD | 1786797 | phosphopantetheinyltransferase component of enterobactin synthase multienzyme complex |
| fepA | 1786798 | iron-enterobactin outer membrane transporter |
| fes | 1786799 | enterobactin/ferric enterobactin esterase |
| ybdZ | 87081760 | conserved protein |
| entF | 1786801 | enterobactin synthase multienzyme complex component, ATP-dependent |
| fepE | 1786802 | regulator of length of O-antigen component of lipopolysaccharide chains |
| fepC | 1786803 | iron-enterobactin transporter subunit |
| fepG | 1786804 | iron-enterobactin transporter subunit |
| fepD | 1786805 | iron-enterobactin transporter subunit |
| entS | 1786806 | predicted transporter |
| fepB | 1786807 | iron-enterobactin transporter subunit |
| entC | 1786809 | isochorismate synthase 1 |
| entE | 1786810 | 2,3-dihydroxybenzoate-AMP ligase component of enterobactin synthase multienzyme complex |
| entB | 1786811 | isochorismatase |
| entA | 1786812 | 2,3-dihydro-2,3-dihydroxybenzoate dehydrogenase |
| entH | 1786813 | conserved protein |
| cstA | 1786814 | carbon starvation protein |
| ybdD | 87081761 | conserved protein |
| ybdH | 1786815 | predicted oxidoreductase |
| ahpC | 1786822 | alkyl hydroperoxide reductase, C22 subunit |
| ahpF | 87081763 | alkyl hydroperoxide reductase, F52a subunit, FAD/NAD(P)-binding |
| uspG | 1786824 | universal stress protein UP12 |
| pagP | 1786840 | palmitoyl transferase for Lipid A |
| cspE | 1786841 | DNA-binding transcriptional repressor |
| crcB | 1786842 | predicted inner membrane protein associated with chromosome condensation |
| tatE | 1786845 | TatABCE protein translocation system subunit |
| lipA | 1786846 | lipoate synthase |
| lipB | 87081767 | lipoyl-protein ligase |
| ybeD | 1786850 | conserved protein |
| dacA | 1786851 | D-alanyl-D-alanine carboxypeptidase (penicillin-binding protein 5) |
| ybeB | 87081768 | predicted protein |
| holA | 1786859 | DNA polymerase III, delta subunit |
| lptE | 1786860 | minor lipoprotein |
| leuS | 1786861 | leucyl-tRNA synthetase |
| ybeL | 1786863 | conserved protein |
| gltK | 1786873 | glutamate and aspartate transporter subunit |
| gltJ | 1786874 | glutamate and aspartate transporter subunit |
| gltI | 1786876 | glutamate and aspartate transporter subunit |
| lnt | 1786878 | apolipoprotein N-acyltransferase |
| ybeX | 1786879 | predicteed ion transport |
| ybeY | 1786880 | conserved protein |
| ybeZ | 145693103 | predicted protein with nucleoside triphosphate hydrolase domain |
| miaB | 1786882 | isopentenyl-adenosine A37 tRNA methylthiolase |
| asnB | 1786889 | asparagine synthetase B |
| nagD | 1786890 | UMP phosphatase |
| nagC | 1786891 | DNA-binding transcriptional dual regulator, repressor of N-acetylglucosamine |
| nagA | 1786892 | N-acetylglucosamine-6-phosphate deacetylase |
| nagB | 1786893 | glucosamine-6-phosphate deaminase |
| nagE | 1786894 | fused N-acetyl glucosamine specific PTS enzyme: IIC, IIB , and IIA components |
| ybfN | 1786898 | predicted lipoprotein |
| fur | 1786899 | DNA-binding transcriptional dual regulator of siderophore biosynthesis and transport |
| pgm | 1786904 | phosphoglucomutase |
| speF | 1786909 | ornithine decarboxylase isozyme, inducible |
| kdpE | 1786911 | DNA-binding response regulator in two-component regulatory system with KdpD |
| kdpD | 1786912 | fused sensory histidine kinase in two-component regulatory system with KdpE: signal sensing protein |
| kdpC | 1786913 | potassium translocating ATPase, subunit C |
| kdpB | 1786914 | potassium translocating ATPase, subunit B |
| kdpA | 1786915 | potassium translocating ATPase, subunit A |
| kdpF | 87081773 | potassium ion accessory transporter subunit |
| ybfA | 1786916 | predicted protein |
| ybgA | 1786925 | conserved protein |
| phr | 1786926 | deoxyribodipyrimidine photolyase, FAD-binding |
| ybgH | 1786927 | predicted transporter |
| ybgI | 1786928 | conserved metal-binding protein |
| ybgJ | 1786929 | predicted enzyme subunit |
| ybgK | 1786930 | predicted enzyme subunit |
| ybgL | 1786931 | predicted lactam utilization protein |
| nei | 1786932 | endonuclease VIII/ 5-formyluracil/5-hydroxymethyluracil DNA glycosylase |
| abrB | 87081776 | predicted regulator |
| gltA | 1786939 | citrate synthase |
| sdhC | 1786940 | succinate dehydrogenase, membrane subunit, binds cytochrome b556 |
| sdhD | 1786941 | succinate dehydrogenase, membrane subunit, binds cytochrome b556 |
| sdhA | 1786942 | succinate dehydrogenase, flavoprotein subunit |
| sdhB | 1786943 | succinate dehydrogenase, FeS subunit |
| sucA | 1786945 | 2-oxoglutarate decarboxylase, thiamin-requiring |
| sucB | 1786946 | dihydrolipoyltranssuccinase |
| sucC | 1786948 | succinyl-CoA synthetase, beta subunit |
| sucD | 1786949 | succinyl-CoA synthetase, NAD(P)-binding, alpha subunit |
| cydA | 87081779 | cytochrome d terminal oxidase, subunit I |
| ybgE | 1786956 | conserved inner membrane protein |
| ybgC | 1786957 | predicted acyl-CoA thioesterase |
| nadA | 1786964 | quinolinate synthase, subunit A |
| pnuC | 1786965 | predicted nicotinamide mononucleotide transporter |
| ybgS | 1786968 | conserved protein |
| aroG | 1786969 | 3-deoxy-D-arabino-heptulosonate-7-phosphate synthase, phenylalanine repressible |
| gpmA | 1786970 | phosphoglyceromutase 1 |
| galM | 1786971 | galactose-1-epimerase (mutarotase) |
| galK | 1786972 | galactokinase |
| galT | 1786973 | galactose-1-phosphate uridylyltransferase |
| galE | 1786974 | UDP-galactose-4-epimerase |
| modF | 1786975 | fused molybdate transporter subunits of ABC superfamily: ATP-binding components |
| modE | 1786976 | DNA-binding transcriptional dual regulator |
| ybhT | 1786977 | predicted protein |
| modA | 1786979 | molybdate transporter subunit |
| modB | 1786980 | molybdate transporter subunit |
| modC | 1786981 | molybdate transporter subunit |
| pgl | 1786983 | 6-phosphogluconolactonase |
| ybhB | 1786990 | predicted kinase inhibitor |
| bioA | 1786991 | 7,8-diaminopelargonic acid synthase, PLP-dependent |
| bioB | 1786992 | biotin synthase |
| bioF | 1786993 | 8-amino-7-oxononanoate synthase |
| bioC | 1786994 | predicted methltransferase, enzyme of biotin synthesis |
| bioD | 1786995 | dethiobiotin synthetase |
| uvrB | 1786996 | excinulease of nucleotide excision repair, DNA damage recognition component |
| ybhK | 1786997 | predicted transferase with NAD(P)-binding Rossmann-fold domain |
| moaA | 1786999 | molybdopterin biosynthesis protein A |
| moaB | 1787000 | molybdopterin biosynthesis protein B |
| moaC | 1787001 | molybdopterin biosynthesis, protein C |
| moaD | 1787002 | molybdopterin synthase, small subunit |
| moaE | 1787003 | molybdopterin synthase, large subunit |
| ybhO | 1787007 | cardiolipin synthase 2 |
| ybhP | 1787008 | predicted DNase |
| ybhQ | 1787009 | predicted inner membrane protein |
| ybhR | 1787010 | predicted transporter subunit: membrane component of ABC superfamily |
| ybhS | 1787011 | predicted transporter subunit: membrane component of ABC superfamily |
| ybhG | 1787013 | predicted membrane fusion protein (MFP) component of efflux pump, membrane anchor |
| ybiH | 87081783 | predicted DNA-binding transcriptional regulator |
| rhlE | 1787016 | RNA helicase |
| dinG | 1787018 | ATP-dependent DNA helicase |
| ybiB | 1787019 | predicted transferase/phosphorylase |
| ybiC | 1787020 | predicted dehydrogenase |
| ybiJ | 1787021 | predicted protein |
| ybiI | 1787022 | conserved protein |
| ybiX | 87081784 | conserved protein |
| fiu | 1787024 | predicted iron outer membrane transporter |
| mcbA | 87081785 | predicted protein |
| rlmF | 87081786 | 23S rRNA mA1618 methyltransferase, SAM-dependent |
| glnQ | 1787029 | glutamine transporter subunit |
| glnP | 1787030 | glutamine transporter subunit |
| glnH | 1787031 | glutamine transporter subunit |
| dps | 1787032 | Fe-binding and storage protein |
| ompX | 1787034 | outer membrane protein |
| ybiP | 1787035 | predicted hydrolase, inner membrane |
| yliL | 1787037 | predicted protein |
| mntR | 1787038 | DNA-binding transcriptional regulator of mntH |
| ybiR | 1787039 | predicted transporter |
| ybiS | 1787040 | L,D-transpeptidase linking Lpp to murein |
| ybiT | 1787041 | fused predicted transporter subunits of ABC superfamily: ATP-binding components |
| ybiV | 1787043 | predicted hydrolase |
| ybiW | 1787044 | predicted pyruvate formate lyase |
| ybiY | 1787045 | predicted pyruvate formate lyase activating enzyme |
| fsaA | 87081788 | fructose-6-phosphate aldolase 1 |
| moeB | 1787048 | molybdopterin synthase sulfurylase |
| moeA | 1787049 | molybdopterin biosynthesis protein |
| iaaA | 1787050 | L-asparaginase |
| gsiA | 145693107 | fused predicted peptide transport subunits of ABC superfamily: ATP-binding components |
| gsiB | 1787052 | predicted peptide transporter subunit: periplasmic-binding component of ABC superfamily |
| gsiC | 1787053 | predicted peptide transporter subunit: membrane component of ABC superfamily |
| gsiD | 1787054 | predicted peptide transporter subunit: membrane component of ABC superfamily |
| yliJ | 87081789 | predicted glutathione S-transferase |
| dacC | 1787062 | D-alanyl-D-alanine carboxypeptidase (penicillin-binding protein 6a) |
| deoR | 1787063 | DNA-binding transcriptional repressor |
| ybjG | 1787064 | undecaprenyl pyrophosphate phosphatase |
| cmr | 1787065 | multidrug efflux system protein |
| ybjJ | 1787068 | predicted transporter |
| ybjK | 1787069 | predicted DNA-binding transcriptional regulator |
| ybjL | 1787071 | predicted transporter |
| ybjM | 1787072 | predicted inner membrane protein |
| grxA | 1787073 | glutaredoxin 1, redox coenzyme for ribonucleotide reductase (RNR1a) |
| ybjC | 1787074 | predicted inner membrane protein |
| nfsA | 1787075 | nitroreductase A, NADPH-dependent, FMN-dependent |
| rimK | 1787076 | ribosomal protein S6 modification protein |
| ybjN | 1787077 | predicted oxidoreductase |
| potF | 1787078 | putrescine transporter subunit: periplasmic-binding component of ABC superfamily |
| potG | 87081791 | putrescine transporter subunit: ATP-binding component of ABC superfamily |
| potH | 1787080 | putrescine transporter subunit: membrane component of ABC superfamily |
| potI | 1787081 | putrescine transporter subunit: membrane component of ABC superfamily |
| ybjO | 1787082 | predicted inner membrane protein |
| rumB | 1787083 | 23S rRNA m(5)U747 methyltransferase |
| artJ | 1787085 | arginine transporter subunit |
| artM | 1787086 | arginine transporter subunit |
| artQ | 1787087 | arginine transporter subunit |
| artI | 1787088 | arginine transporter subunit |
| artP | 1787089 | arginine transporter subunit |
| ybjP | 1787090 | predicted lipoprotein |
| ybjQ | 1787091 | conserved protein |
| amiD | 1787092 | predicted amidase and lipoprotein |
| ybjS | 87081792 | predicted NAD(P)H-binding oxidoreductase with NAD(P)-binding Rossmann-fold domain |
| ybjT | 87081793 | conserved protein with NAD(P)-binding Rossmann-fold domain |
| ltaE | 1787095 | L-allo-threonine aldolase, PLP-dependent |
| poxB | 1787096 | pyruvate dehydrogenase (pyruvate oxidase), thiamin-dependent, FAD-binding |
| hcr | 1787098 | HCP oxidoreductase, NADH-dependent |
| hcp | 87081794 | hybrid-cluster [4Fe-2S-2O] protein in anaerobic terminal reductases |
| ybjE | 87081795 | predicted transporter |
| ybjD | 1787102 | conserved protein with nucleoside triphosphate hydrolase domain |
| ybjX | 1787103 | conserved protein |
| macA | 87081796 | macrolide transporter subunit, membrane fusion protein (MFP) component |
| macB | 1787105 | fused macrolide transporter subunits of ABC superfamily: ATP-binding component/membrane component |
| cspD | 1787107 | cold shock protein homolog |
| clpS | 1787108 | regulatory protein for ClpA substrate specificity |
| clpA | 1787109 | ATPase and specificity subunit of ClpA-ClpP ATP-dependent serine protease, chaperone activity |
| aat | 1787111 | leucyl/phenylalanyl-tRNA-protein transferase |
| cydC | 1787112 | fused cysteine transporter subunits of ABC superfamily: membrane component/ATP-binding component |
| cydD | 1787113 | fused cysteine transporter subunits of ABC superfamily: membrane component/ATP-binding component |
| trxB | 1787114 | thioredoxin reductase, FAD/NAD(P)-binding |
| lolA | 145693108 | chaperone for lipoproteins |
| rarA | 1787119 | recombination protein |
| serS | 1787120 | seryl-tRNA synthetase, also charges selenocysteinyl-tRNA with serine |
| dmsA | 87081797 | dimethyl sulfoxide reductase, anaerobic, subunit A |
| dmsB | 1787122 | dimethyl sulfoxide reductase, anaerobic, subunit B |
| ycaC | 1787124 | predicted hydrolase |
| ycaD | 1787126 | predicted transporter |
| pflA | 1787130 | pyruvate formate lyase activating enzyme 1 |
| pflB | 1787131 | pyruvate formate lyase I |
| focA | 1787132 | formate transporter |
| ycaO | 87081799 | conserved protein |
| ycaP | 1787135 | conserved inner membrane protein |
| serC | 1787136 | 3-phosphoserine/phosphohydroxythreonine aminotransferase |
| aroA | 1787137 | 5-enolpyruvylshikimate-3-phosphate synthetase |
| ycaL | 87081800 | predicted peptidase with chaperone function |
| cmk | 1787139 | cytidylate kinase |
| rpsA | 1787140 | 30S ribosomal subunit protein S1 |
| ihfB | 1787141 | integration host factor (IHF), DNA-binding protein, beta subunit |
| ycaI | 87081801 | conserved inner membrane protein |
| msbA | 1787143 | fused lipid transporter subunits of ABC superfamily: membrane component/ATP-binding component |
| lpxK | 1787144 | lipid A 4'kinase |
| ycaQ | 1787145 | conserved protein |
| ycaR | 1787146 | conserved protein |
| kdsB | 1787147 | 3-deoxy-manno-octulosonate cytidylyltransferase |
| ycbJ | 1787149 | conserved protein |
| ycbC | 1787150 | conserved inner membrane protein |
| smtA | 1787151 | predicted S-adenosyl-L-methionine-dependent methyltransferase |
| mukF | 1787152 | Involved in chromosome partioning, Ca2+ binding protein |
| mukE | 1787153 | protein involved in chromosome partitioning |
| mukB | 1787154 | fused chromosome partitioning protein: predicted nucleotide hydrolase/conserved protein/conserved protein |
| ycbB | 1787155 | murein L,D-transpeptidase |
| ycbK | 1787157 | conserved protein |
| ycbL | 1787158 | predicted metal-binding enzyme |
| ompF | 1787160 | outer membrane porin 1a (Ia;b;F) |
| asnS | 1787161 | asparaginyl tRNA synthetase |
| pncB | 1787162 | nicotinate phosphoribosyltransferase |
| pepN | 1787163 | aminopeptidase N |
| ssuB | 1787164 | alkanesulfonate transporter subunit |
| ssuC | 87081802 | alkanesulfonate transporter subunit |
| ssuD | 1787166 | alkanesulfonate monooxygenase, FMNH(2)-dependent |
| ssuA | 87081803 | alkanesulfonate transporter subunit |
| ssuE | 1787168 | NAD(P)H-dependent FMN reductase |
| pyrD | 1787177 | dihydro-orotate oxidase, FMN-linked |
| ymbA | 145693109 | conserved protein |
| yccS | 87081808 | predicted inner membrane protein |
| yccF | 1787195 | conserved inner membrane protein |
| mgsA | 87081809 | methylglyoxal synthase |
| yccT | 1787198 | conserved protein |
| yccU | 87081810 | predicted CoA-binding protein with NAD(P)-binding Rossmann-fold domain |
| hspQ | 145693110 | DNA-binding protein, hemimethylated |
| rlmI | 145693111 | 23S rRNA m(5)C1962 methyltransferase, SAM-dependent |
| yccX | 1787203 | predicted acylphosphatase |
| yccK | 87081811 | predicted sulfite reductase subunit |
| hyaA | 1787206 | hydrogenase 1, small subunit |
| hyaC | 1787208 | hydrogenase 1, b-type cytochrome subunit |
| hyaD | 1787209 | protein involved in processing of HyaA and HyaB proteins |
| hyaE | 1787210 | protein involved in processing of HyaA and HyaB proteins |
| hyaF | 1787211 | protein involved in nickel incorporation into hydrogenase-1 proteins |
| appC | 1787212 | cytochrome bd-II oxidase, subunit I |
| appB | 1787213 | cytochrome bd-II oxidase, subunit II |
| yccB | 145693112 | hypothetical protein |
| appA | 1787215 | phosphoanhydride phosphorylase |
| gfcE | 1787218 | predicted exopolysaccharide export protein |
| gfcD | 1787219 | conserved protein |
| cspH | 2367112 | stress protein, member of the CspA-family |
| cspG | 2367114 | DNA-binding transcriptional regulator |
| torT | 1787228 | periplasmic sensory protein associated with the TorRS two-component regulatory system |
| torR | 1787229 | DNA-binding response regulator in two-component regulatory system with TorS |
| torC | 1787230 | trimethylamine N-oxide (TMAO) reductase I, cytochrome c-type subunit |
| torA | 1787231 | trimethylamine N-oxide (TMAO) reductase I, catalytic subunit |
| cbpM | 1787234 | modulator of CbpA co-chaperone |
| cbpA | 1787235 | curved DNA-binding protein, DnaJ homologue that functions as a co-chaperone of DnaK |
| rutR | 1787249 | predicted DNA-binding transcriptional regulator |
| ghrA | 87081824 | 2-ketoacid reductase |
| ycdX | 1787271 | predicted zinc-binding hydrolase |
| ycdY | 1787272 | conserved protein |
| ycdZ | 87081825 | predicted inner membrane protein |
| ymdA | 1787281 | predicted protein |
| ymdB | 1787283 | conserved protein |
| ymdC | 87081826 | predicted hydrolase |
| opgC | 1787285 | membrane protein required for modification of periplasmic glucan |
| opgG | 1787286 | glucan biosynthesis protein, periplasmic |
| lpxL | 1787292 | lauryl-acyl carrier protein (ACP)-dependent acyltransferase |
| yceI | 1787295 | predicted protein |
| yceJ | 1787296 | predicted cytochrome b561 |
| solA | 1787298 | N-methyltryptophan oxidase, FAD-binding |
| bssS | 1787299 | predicted protein |
| dinI | 1787300 | DNA damage-inducible protein I |
| pyrC | 1787301 | dihydro-orotase |
| yceB | 1787302 | predicted lipoprotein |
| grxB | 1787303 | glutaredoxin 2 (Grx2) |
| mdtH | 87081828 | predicted drug efflux system |
| rimJ | 1787305 | ribosomal-protein-S5-alanine N-acetyltransferase |
| yceH | 1787306 | conserved protein |
| yceM | 1787307 | predicted oxidoreductase with NAD(P)-binding Rossmann-fold domain |
| murJ | 1787309 | predicted inner membrane protein |
| flgN | 1787310 | export chaperone for FlgK and FlgL |
| flgM | 1787311 | anti-sigma factor for FliA (sigma 28) |
| flgA | 1787312 | assembly protein for flagellar basal-body periplasmic P ring |
| flgB | 1787313 | flagellar component of cell-proximal portion of basal-body rod |
| flgC | 1787314 | flagellar component of cell-proximal portion of basal-body rod |
| flgD | 1787315 | flagellar hook assembly protein |
| flgE | 1787316 | flagellar hook protein |
| flgI | 1787320 | predicted flagellar basal body protein |
| flgJ | 1787321 | muramidase |
| flgK | 1787323 | flagellar hook-filament junction protein 1 |
| flgL | 1787324 | flagellar hook-filament junction protein |
| rne | 1787325 | fused ribonucleaseE: endoribonuclease/RNA-binding protein/RNA degradosome binding protein |
| ptsG | 1787343 | fused glucose-specific PTS enzymes: IIB component/IIC component |
| fhuE | 1787344 | ferric-rhodotorulic acid outer membrane transporter |
| hinT | 1787346 | purine nucleoside phosphoramidase |
| ycfL | 1787347 | predicted protein |
| ycfM | 1787348 | predicted outer membrane lipoprotein |
| thiK | 1787349 | thiamin kinase |
| nagZ | 1787350 | beta N-acetyl-glucosaminidase |
| ycfP | 87081832 | conserved protein |
| ndh | 1787352 | respiratory NADH dehydrogenase 2/cupric reductase |
| ycfJ | 1787353 | predicted protein |
| ycfQ | 87081833 | predicted DNA-binding transcriptional regulator |
| bhsA | 1787355 | predicted protein |
| ycfS | 1787356 | L,D-transpeptidase linking Lpp to murein |
| mfd | 1787357 | transcription-repair coupling factor |
| ycfT | 1787359 | predicted inner membrane protein |
| lolC | 1787360 | outer membrane-specific lipoprotein transporter subunit |
| lolD | 87081834 | outer membrane-specific lipoprotein transporter subunit |
| lolE | 1787362 | outer membrane-specific lipoprotein transporter subunit |
| nagK | 1787363 | N-acetyl-D-glucosamine kinase |
| cobB | 1787364 | deacetylase of acetyl-CoA synthetase, NAD-dependent |
| potD | 1787367 | polyamine transporter subunit |
| potC | 1787368 | polyamine transporter subunit |
| potB | 1787369 | polyamine transporter subunit |
| potA | 1787370 | polyamine transporter subunit |
| pepT | 1787372 | peptidase T |
| ycfD | 87081836 | conserved protein |
| phoQ | 1787374 | sensory histidine kinase in two-component regulatory system with PhoP |
| phoP | 1787375 | DNA-binding response regulator in two-component regulatory system with PhoQ |
| purB | 1787376 | adenylosuccinate lyase |
| hflD | 1787377 | predicted lysogenization regulator |
| mnmA | 87081837 | tRNA (5-methylaminomethyl-2-thiouridylate)-methyltransferase |
| nudJ | 1787379 | bifunctional thiamin pyrimidine pyrophosphate hydrolase/ thiamin pyrophosphate hydrolase |
| rluE | 87081838 | 16S RNA U516 pseudouridine synthase |
| icd | 1787381 | e14 prophage; isocitrate dehydrogenase, specific for NADP+ |
| ymgJ | 145693116 | hypothetical protein |
| minE | 1787422 | cell division topological specificity factor |
| minD | 1787423 | membrane ATPase of the MinC-MinD-MinE system |
| minC | 1787424 | cell division inhibitor |
| ycgN | 145693117 | conserved protein |
| umuD | 1787431 | DNA polymerase V, subunit D |
| dsbB | 1787433 | oxidoreductase that catalyzes reoxidation of DsbA protein disulfide isomerase I |
| nhaB | 1787435 | sodium:proton antiporter |
| ycgB | 1787437 | conserved protein |
| dadX | 1787439 | alanine racemase 2, PLP-binding |
| cvrA | 87081854 | predicted cation/proton antiporter |
| ldcA | 1787441 | L,D-carboxypeptidase A |
| emtA | 87081855 | lytic murein endotransglycosylase E |
| ycgR | 1787443 | protein involved in flagellar function |
| ymgE | 1787445 | predicted inner membrane protein |
| ychF | 1787454 | predicted GTP-binding protein |
| pth | 1787455 | peptidyl-tRNA hydrolase |
| ychH | 1787456 | predicted inner membrane protein |
| ychM | 87081859 | predicted transporter |
| prs | 1787458 | phosphoribosylpyrophosphate synthase |
| hemA | 1787461 | glutamyl tRNA reductase |
| prfA | 1787462 | peptide chain release factor RF-1 |
| prmC | 1787463 | N5-glutamine methyltransferase, modifies release factors RF-1 and RF-2 |
| ychQ | 1787464 | predicted transcriptional regulator |
| ychA | 1787465 | predicted transcriptional regulator |
| kdsA | 1787466 | 3-deoxy-D-manno-octulosonate 8-phosphate synthase |
| ldrA | 48994880 | toxic polypeptide, small |
| ldrB | 48994881 | toxic polypeptide, small |
| ldrC | 48994882 | toxic polypeptide, small |
| chaA | 1787468 | calcium/sodium:proton antiporter |
| chaB | 1787469 | predicted cation regulator |
| chaC | 145693119 | regulatory protein for cation transport |
| ychN | 1787471 | conserved protein |
| ychO | 145693120 | predicted invasin |
| narL | 1787473 | DNA-binding response regulator in two-component regulatory system with NarX (or NarQ) |
| narX | 1787474 | sensory histidine kinase in two-component regulatory system with NarL |
| narK | 1787475 | nitrate/nitrite transporter |
| narG | 1787477 | nitrate reductase 1, alpha subunit |
| narH | 1787478 | nitrate reductase 1, beta (Fe-S) subunit |
| narJ | 1787479 | molybdenum-cofactor-assembly chaperone subunit (delta subunit) of nitrate reductase 1 |
| narI | 1787480 | nitrate reductase 1, gamma (cytochrome b(NR)) subunit |
| purU | 1787483 | formyltetrahydrofolate hydrolase |
| ychJ | 1787484 | conserved protein |
| rssA | 1787485 | conserved protein |
| rssB | 1787487 | response regulator of RpoS |
| galU | 1787488 | glucose-1-phosphate uridylyltransferase |
| hns | 1787489 | global DNA-binding transcriptional dual regulator H-NS |
| tdk | 1787490 | thymidine kinase/deoxyuridine kinase |
| adhE | 1787493 | fused acetaldehyde-CoA dehydrogenase/iron-dependent alcohol dehydrogenase/pyruvate-formate lyase deactivase |
| oppA | 1787495 | oligopeptide transporter subunit |
| oppB | 1787497 | oligopeptide transporter subunit |
| oppC | 1787498 | oligopeptide transporter subunit |
| oppD | 48994883 | oligopeptide transporter subunit |
| oppF | 1787500 | oligopeptide transporter subunit |
| yciU | 87081860 | predicted protein |
| cls | 1787502 | cardiolipin synthase 1 |
| yciY | 145693122 | hypothetical protein |
| kch | 1787503 | voltage-gated potassium channel |
| yciI | 87081861 | predicted enzyme |
| tonB | 1787505 | membrane spanning protein in TonB-ExbB-ExbD complex |
| yciA | 1787506 | predicted hydrolase |
| yciB | 1787507 | predicted inner membrane protein |
| yciC | 1787508 | predicted inner membrane protein |
| ompW | 1787510 | outer membrane protein W |
| trpA | 1787514 | tryptophan synthase, alpha subunit |
| trpB | 1787515 | tryptophan synthase, beta subunit |
| trpC | 87081863 | fused indole-3-glycerolphosphate synthetase/N-(5-phosphoribosyl)anthranilate isomerase |
| trpD | 1787517 | fused glutamine amidotransferase (component II) of anthranilate synthase/anthranilate phosphoribosyl transferase |
| trpE | 1787518 | component I of anthranilate synthase |
| yciV | 1787520 | conserved protein |
| yciO | 87081864 | conserved protein |
| rluB | 1787524 | 23S rRNA pseudouridylate synthase |
| btuR | 1787525 | cob(I)alamin adenolsyltransferase/cobinamide ATP-dependent adenolsyltransferase |
| yciK | 1787526 | predicted oxoacyl-(acyl carrier protein) reductase, EmrKY-TolC system |
| sohB | 1787527 | predicted inner membrane peptidase |
| yciN | 1787528 | predicted protein |
| topA | 1787529 | DNA topoisomerase I, omega subunit |
| cysB | 1787530 | DNA-binding transcriptional dual regulator, O-acetyl-L-serine-binding |
| yciX | 145693123 | hypothetical protein |
| ribA | 1787533 | GTP cyclohydrolase II |
| pgpB | 1787534 | phosphatidylglycerophosphatase B |
| yciS | 1787535 | conserved inner membrane protein |
| pyrF | 1787537 | orotidine-5'-phosphate decarboxylase |
| yciH | 87081867 | conserved protein |
| yciT | 1787540 | predicted DNA-binding transcriptional regulator |
| yciZ | 145693124 | hypothetical protein |
| gmr | 1787541 | modulator of Rnase II stability |
| rnb | 1787542 | ribonuclease II |
| yciW | 87081868 | predicted oxidoreductase |
| fabI | 1787545 | enoyl-[acyl-carrier-protein] reductase, NADH-dependent |
| sapF | 1787547 | predicted antimicrobial peptide transporter subunit |
| sapD | 1787548 | predicted antimicrobial peptide transporter subunit |
| sapC | 1787549 | predicted antimicrobial peptide transporter subunit |
| sapB | 1787550 | predicted antimicrobial peptide transporter subunit |
| sapA | 1787551 | predicted antimicrobial peptide transporter subunit |
| ymjA | 1787552 | predicted protein |
| puuP | 87081869 | putrescine importer |
| pspF | 87081872 | DNA-binding transcriptional activator |
| pspA | 2367118 | regulatory protein for phage-shock-protein operon |
| pspB | 1787563 | DNA-binding transcriptional regulator of psp operon |
| pspC | 1787564 | DNA-binding transcriptional activator |
| pspD | 1787565 | peripheral inner membrane phage-shock protein |
| pspE | 1787567 | thiosulfate:cyanide sulfurtransferase (rhodanese) |
| ycjX | 1787581 | conserved protein with nucleoside triphosphate hydrolase domain |
| ycjF | 1787582 | conserved inner membrane protein |
| tyrR | 1787583 | DNA-binding transcriptional dual regulator, tyrosine-binding |
| tpx | 1787584 | lipid hydroperoxide peroxidase |
| ycjG | 87081876 | L-Ala-D/L-Glu epimerase |
| mpaA | 1787586 | murein peptide amidase A |
| mppA | 87081878 | murein tripeptide (L-ala-gamma-D-glutamyl-meso-DAP) transporter subunit |
| ynaI | 1787591 | conserved inner membrane protein |
| uspE | 1787594 | stress-induced protein |
| fnr | 1787595 | DNA-binding transcriptional dual regulator, global regulator of anaerobic growth |
| ydaL | 1787602 | conserved protein |
| ydaN | 1787604 | predicted Zn(II) transporter |
| ttcA | 1787606 | predicted C32 tRNA thiolase |
| uspF | 87081893 | stress-induced protein, ATP-binding protein |
| ompN | 1787641 | outer membrane pore protein N, non-specific |
| ydbK | 1787642 | fused predicted pyruvate-flavodoxin oxidoreductase: conserved protein/conserved protein/FeS binding protein |
| ydbJ | 87081894 | predicted protein |
| ldhA | 1787645 | fermentative D-lactate dehydrogenase, NAD-dependent |
| ydbH | 1787646 | predicted protein |
| ynbE | 1787647 | predicted lipoprotein |
| azoR | 1787680 | NADH-azoreductase, FMN-dependent |
| hrpA | 145693127 | ATP-dependent helicase |
| ydcX | 87081912 | predicted inner membrane protein |
| narV | 1787738 | nitrate reductase 2 (NRZ), gamma subunit |
| narY | 1787740 | nitrate reductase 2 (NRZ), beta subunit |
| narZ | 1787741 | nitrate reductase 2 (NRZ), alpha subunit |
| narU | 1787743 | nitrate/nitrite transporter |
| yddG | 145693132 | predicted methyl viologen efflux pump |
| fdnG | 3868719 | formate dehydrogenase-N, alpha subunit, nitrate-inducible |
| fdnH | 1787749 | formate dehydrogenase-N, Fe-S (beta) subunit, nitrate-inducible |
| fdnI | 1787750 | formate dehydrogenase-N, cytochrome B556 (gamma) subunit, nitrate-inducible |
| yddM | 145693133 | predicted DNA-binding transcriptional regulator |
| adhP | 87081918 | ethanol-active dehydrogenase/acetaldehyde-active reductase |
| osmC | 1787757 | osmotically inducible, stress-inducible membrane protein |
| yddW | 1787767 | predicted liprotein |
| gadC | 1787768 | predicted glutamate:gamma-aminobutyric acid antiporter |
| gadB | 1787769 | glutamate decarboxylase B, PLP-dependent |
| ydeO | 1787776 | predicted DNA-binding transcriptional activator |
| yneN | 1787777 | Two component system connector membrane protein, EvgSA to PhoQP |
| ydeP | 1787778 | predicted oxidoreductase |
| ydeA | 1787808 | predicted arabinose transporter |
| marC | 1787809 | conserved protein |
| marR | 87081927 | DNA-binding transcriptional repressor of multiple antibiotic resistance |
| marA | 87081928 | DNA-binding transcriptional dual activator of multiple antibiotic resistance |
| marB | 1787812 | predicted protein |
| dcp | 1787819 | dipeptidyl carboxypeptidase II |
| ydfG | 1787820 | L-allo-threonine dehydrogenase, NAD(P)-binding |
| ydfZ | 1787822 | conserved protein |
| ydfI | 1787823 | predicted mannonate dehydrogenase |
| cspI | 1787834 | Qin prophage; cold shock protein |
| essQ | 87081934 | Qin prophage; predicted S lysis protein |
| cspB | 1787839 | Qin prophage; cold shock protein |
| cspF | 1787840 | Qin prophage; cold shock protein |
| hokD | 1787845 | Qin prophage; small toxic polypeptide |
| ynfE | 1787870 | oxidoreductase subunit |
| ynfF | 171474008 | oxidoreductase subunit |
| ynfG | 1787872 | oxidoreductase, Fe-S subunit |
| dmsD | 87081942 | twin-argninine leader-binding protein for DmsA and TorA |
| clcB | 87081943 | predicted voltage-gated chloride channel |
| ynfK | 87081944 | predicted dethiobiotin synthetase |
| dgsA | 1787878 | DNA-binding transcriptional repressor |
| ynfL | 1787879 | predicted DNA-binding transcriptional regulator |
| ynfM | 1787880 | predicted transporter |
| asr | 87081945 | acid shock-inducible periplasmic protein |
| ydgU | 145693139 | hypothetical protein |
| ydgD | 1787882 | predicted peptidase |
| mdtI | 1787883 | multidrug efflux system transporter |
| tqsA | 1787885 | predicted inner membrane protein |
| pntB | 1787886 | pyridine nucleotide transhydrogenase, beta subunit |
| pntA | 1787887 | pyridine nucleotide transhydrogenase, alpha subunit |
| ydgH | 1787889 | predicted protein |
| ydgI | 1787890 | predicted arginine/ornithine antiporter transporter |
| folM | 1787891 | dihydrofolate reductase isozyme |
| ydgC | 1787892 | conserved inner membrane protein associated with alginate biosynthesis |
| rstA | 145693140 | DNA-binding response regulator in two-component regulatory system with RstB |
| tus | 1787895 | inhibitor of replication at Ter, DNA-binding protein |
| fumC | 1787896 | fumarate hydratase (fumarase C),aerobic Class II |
| fumA | 1787897 | fumarate hydratase (fumarase A), aerobic Class I |
| manA | 1787899 | mannose-6-phosphate isomerase |
| ydgA | 1787900 | conserved protein |
| uidC | 87081946 | predicted outer membrane porin protein |
| uidA | 1787903 | beta-D-glucuronidase |
| uidR | 1787904 | DNA-binding transcriptional repressor |
| hdhA | 1787905 | 7-alpha-hydroxysteroid dehydrogenase, NAD-dependent |
| malI | 1787906 | DNA-binding transcriptional repressor |
| malX | 1787908 | fused maltose and glucose-specific PTS enzymes: IIB component, IIC component |
| malY | 1787909 | bifunctional beta-cystathionase, PLP-dependent/ regulator of maltose regulon |
| add | 1787910 | adenosine deaminase |
| ydgJ | 87081947 | predicted oxidoreductase |
| blr | 48994886 | beta-lactam resistance membrane protein |
| cnu | 1787912 | predicted regulator |
| ydgK | 87081948 | conserved inner membrane protein |
| rsxA | 1787914 | predicted inner membrane subunit |
| rsxB | 1787915 | predicted iron-sulfur protein |
| rsxC | 1787916 | fused predicted 4Fe-4S ferredoxin-type protein/conserved protein |
| rsxD | 1787917 | predicted inner membrane oxidoreductase |
| rsxG | 1787918 | predicted oxidoreductase |
| rsxE | 1787919 | predicted inner membrane NADH-quinone reductase |
| nth | 1787920 | DNA glycosylase and apyrimidinic (AP) lyase (endonuclease III) |
| tppB | 1787922 | predicted transporter |
| gst | 1787923 | glutathionine S-transferase |
| pdxY | 1787924 | pyridoxal kinase 2/pyridoxine kinase |
| tyrS | 1787925 | tyrosyl-tRNA synthetase |
| pdxH | 1787926 | pyridoxine 5'-phosphate oxidase |
| anmK | 1787928 | conserved protein |
| slyB | 48994887 | outer membrane lipoprotein |
| slyA | 87081950 | DNA-binding transcriptional activator |
| ydhI | 1787931 | predicted inner membrane protein |
| sodC | 1787934 | superoxide dismutase, Cu, Zn |
| ydhF | 48994888 | predicted oxidoreductase |
| ydhL | 87081952 | conserved protein |
| nemR | 145693141 | predicted DNA-binding transcriptional regulator |
| nemA | 1787939 | N-ethylmaleimide reductase, FMN-linked |
| gloA | 1787940 | glyoxalase I, Ni-dependent |
| rnt | 1787941 | ribonuclease T (RNase T) |
| grxD | 1787943 | conserved protein |
| ydhO | 1787944 | predicted lipoprotein |
| sodB | 1787946 | superoxide dismutase, Fe |
| ydhP | 1787947 | predicted transporter |
| ynhF | 145693142 | hypothetical protein |
| purR | 1787948 | DNA-binding transcriptional repressor, hypoxanthine-binding |
| ydhB | 1787949 | predicted DNA-binding transcriptional regulator |
| ydhC | 48994889 | predicted transporter |
| cfa | 1787951 | cyclopropane fatty acyl phospholipid synthase (unsaturated-phospholipid methyltransferase) |
| ribC | 1787952 | riboflavin synthase, alpha subunit |
| mdtK | 48994890 | multidrug efflux system transporter |
| ydhQ | 1787954 | conserved protein |
| ydhT | 1787958 | conserved protein |
| ydhX | 145693143 | predicted 4Fe-4S ferridoxin-type protein |
| ydhW | 1787961 | predicted protein |
| ydhZ | 1787964 | predicted protein |
| ynhG | 1787968 | murein L,D-transpeptidase |
| sufE | 1787969 | sulfur acceptor protein |
| sufS | 1787970 | selenocysteine lyase, PLP-dependent |
| sufD | 1787971 | component of SufBCD complex |
| sufC | 1787972 | component of SufBCD complex, ATP-binding component of ABC superfamily |
| ydiH | 1787975 | predicted protein |
| ydiI | 1787976 | conserved protein |
| ydiJ | 1787977 | predicted FAD-linked oxidoreductase |
| ydiK | 1787979 | predicted inner membrane protein |
| ydiL | 87081956 | conserved protein |
| ydiB | 1787983 | quinate/shikimate 5-dehydrogenase, NAD(P)-binding |
| aroD | 1787984 | 3-dehydroquinate dehydratase |
| ydiS | 1787991 | predicted oxidoreductase with FAD/NAD(P)-binding domain |
| ydiT | 1787992 | predicted 4Fe-4S ferredoxin-type protein |
| pps | 1787994 | phosphoenolpyruvate synthase |
| ydiA | 1787995 | conserved protein |
| aroH | 1787996 | 3-deoxy-D-arabino-heptulosonate-7-phosphate synthase, tryptophan repressible |
| ydiE | 1787998 | conserved protein |
| ydiU | 1787999 | conserved protein |
| ydiV | 1788000 | conserved protein |
| nlpC | 1788001 | predicted lipoprotein |
| btuD | 1788002 | vitamin B12 transporter subunit : ATP-binding component of ABC superfamily |
| btuE | 1788003 | predicted glutathione peroxidase |
| btuC | 1788004 | vitamin B12 transporter subunit: membrane component of ABC superfamily |
| ihfA | 1788005 | integration host factor (IHF), DNA-binding protein, alpha subunit |
| pheT | 1788006 | phenylalanine tRNA synthetase, beta subunit |
| pheS | 1788007 | phenylalanine tRNA synthetase, alpha subunit |
| rpmI | 1788010 | 50S ribosomal subunit protein L35 |
| pfkB | 1788017 | 6-phosphofructokinase II |
| ydiZ | 1788018 | predicted protein |
| yniA | 1788019 | predicted phosphotransferase/kinase |
| yniB | 1788020 | predicted inner membrane protein |
| yniC | 1788021 | predicted hydrolase |
| ydjM | 145693147 | predicted inner membrane protein regulated by LexA |
| ydjN | 1788024 | predicted transporter |
| osmE | 1788035 | DNA-binding transcriptional activator |
| nadE | 1788036 | NAD synthetase, NH3/glutamine-dependent |
| cho | 1788037 | endonuclease of nucleotide excision repair |
| spy | 1788039 | envelope stress induced periplasmic protein |
| astE | 1788040 | succinylglutamate desuccinylase |
| astB | 1788041 | succinylarginine dihydrolase |
| astD | 1788042 | succinylglutamic semialdehyde dehydrogenase |
| astA | 1788043 | arginine succinyltransferase |
| astC | 1788044 | succinylornithine transaminase, PLP-dependent |
| xthA | 1788046 | exonuclease III |
| ydjY | 87081965 | predicted protein |
| ydjZ | 1788049 | conserved inner membrane protein |
| ynjA | 1788050 | conserved protein |
| ynjC | 145693149 | fused transporter subunits of ABC superfamily: membrane components |
| ynjD | 1788053 | predicted transporter subunit: ATP-binding component of ABC superfamily |
| ynjE | 87081967 | predicted thiosulfate sulfur transferase |
| nudG | 1788056 | pyrimidine (deoxy)nucleoside triphosphate pyrophosphohydrolase |
| ynjH | 1788057 | predicted protein |
| gdhA | 1788059 | glutamate dehydrogenase, NADP-specific |
| topB | 1788061 | DNA topoisomerase III |
| selD | 1788062 | selenophosphate synthase |
| ydjA | 1788063 | predicted oxidoreductase |
| ansA | 1788065 | cytoplasmic L-asparaginase I |
| pncA | 87081970 | nicotinamidase/pyrazinamidase |
| yeaC | 87081972 | conserved protein |
| msrB | 1788077 | methionine sulfoxide reductase B |
| yeaD | 87081973 | conserved protein |
| yeaE | 1788081 | predicted oxidoreductase |
| mipA | 1788082 | scaffolding protein for murein synthesizing machinery |
| yeaQ | 1788096 | conserved inner membrane protein |
| rnd | 1788105 | ribonuclease D |
| fadD | 1788107 | acyl-CoA synthetase (long-chain-fatty-acid--CoA ligase) |
| yeaY | 1788108 | predicted lipoprotein |
| yeaZ | 1788109 | predicted peptidase |
| yoaA | 1788110 | conserved protein with nucleoside triphosphate hydrolase domain |
| yoaB | 87081979 | conserved protein |
| yoaH | 1788113 | conserved protein |
| pabB | 1788114 | aminodeoxychorismate synthase, subunit I |
| nudL | 1788115 | predicted NUDIX hydrolase |
| sdaA | 1788116 | L-serine deaminase I |
| yoaE | 1788119 | fused predicted membrane protein/conserved protein |
| manY | 1788121 | mannose-specific enzyme IIC component of PTS |
| yobD | 1788123 | conserved inner membrane protein |
| cspC | 1788126 | stress protein, member of the CspA-family |
| yebR | 171474009 | free methionine-(R)-sulfoxide reductase |
| yebT | 87081984 | conserved protein |
| rsmF | 87081985 | 16S rRNA m(5)C1407 methyltransferase, SAM-dependent |
| yebW | 87081987 | predicted protein |
| yebY | 1788144 | predicted protein |
| yobA | 1788146 | conserved protein |
| holE | 1788147 | DNA polymerase III, theta subunit |
| yobB | 1788148 | conserved protein |
| exoX | 1788149 | DNA exonuclease X |
| yebE | 1788151 | conserved protein |
| yebF | 145693150 | predicted protein |
| yebG | 1788153 | conserved protein regulated by LexA |
| purT | 1788155 | phosphoribosylglycinamide formyltransferase 2 |
| eda | 1788156 | multifunctional 2-keto-3-deoxygluconate 6-phosphate aldolase and 2-keto-4-hydroxyglutarate aldolase and oxaloacetate decarboxylase |
| edd | 1788157 | 6-phosphogluconate dehydratase |
| zwf | 1788158 | glucose-6-phosphate dehydrogenase |
| yebK | 1788159 | predicted DNA-binding transcriptional regulator |
| pykA | 1788160 | pyruvate kinase II |
| lpxM | 1788161 | myristoyl-acyl carrier protein (ACP)-dependent acyltransferase |
| yebA | 87081989 | predicted peptidase |
| znuA | 87081990 | zinc transporter subunit: periplasmic-binding component of ABC superfamily |
| znuC | 1788165 | zinc transporter subunit: ATP-binding component of ABC superfamily |
| znuB | 1788166 | zinc transporter subunit: membrane component of ABC superfamily |
| ruvA | 1788168 | component of RuvABC resolvasome, regulatory subunit |
| ruvC | 1788170 | component of RuvABC resolvasome, endonuclease |
| yebC | 1788171 | conserved protein |
| nudB | 1788172 | dATP pyrophosphohydrolase |
| aspS | 1788173 | aspartyl-tRNA synthetase |
| yecN | 87081993 | predicted inner membrane protein |
| cmoA | 1788177 | tRNA cmo(5)U34 methyltransferase, SAM-dependent |
| cmoB | 1788178 | tRNA mo(5)U34 methyltransferase, SAM-dependent |
| torZ | 87081994 | trimethylamine N-oxide reductase system III, catalytic subunit |
| cutC | 87081995 | copper homeostasis protein |
| yecM | 87081996 | predicted metal-binding enzyme |
| argS | 1788184 | arginyl-tRNA synthetase |
| flhC | 1788201 | DNA-binding transcriptional dual regulator with FlhD |
| araF | 1788211 | L-arabinose transporter subunit |
| ftnB | 1788212 | predicted ferritin-like protein |
| yecJ | 87081998 | predicted protein |
| yecH | 1788217 | predicted protein |
| yecA | 1788219 | conserved metal-binding protein |
| fliA | 1788231 | RNA polymerase, sigma 28 (sigma F) factor |
| fliS | 1788234 | flagellar protein potentiates polymerization |
| fliT | 1788235 | predicted chaperone |
| amyA | 1788236 | cytoplasmic alpha-amylase |
| yedD | 1788237 | predicted protein |
| yedE | 1788238 | predicted inner membrane protein |
| yedF | 1788239 | conserved protein |
| yedP | 1788265 | conserved protein |
| yedQ | 87082007 | predicted diguanylate cyclase |
| yodC | 1788267 | predicted protein |
| yedI | 1788268 | conserved inner membrane protein |
| yedA | 1788269 | predicted inner membrane protein |
| vsr | 1788270 | DNA mismatch endonuclease of very short patch repair |
| yedW | 87082012 | predicted DNA-binding response regulator in two-component system with YedV |
| cobT | 1788300 | nicotinate-nucleotide dimethylbenzimidazole-P phophoribosyl transferase |
| cobS | 1788301 | cobalamin 5'-phosphate synthase |
| cobU | 1788302 | bifunctional cobinamide kinase/ cobinamide phosphate guanylyltransferase |
| sbcB | 1788321 | exonuclease I |
| yeeY | 87082024 | predicted DNA-binding transcriptional regulator |
| yeeZ | 1788327 | predicted epimerase, with NAD(P)-binding Rossmann-fold domain |
| hisG | 1788330 | ATP phosphoribosyltransferase |
| hisD | 1788331 | bifunctional histidinal dehydrogenase/ histidinol dehydrogenase |
| hisC | 1788332 | histidinol-phosphate aminotransferase |
| hisB | 87082027 | fused histidinol-phosphatase/imidazoleglycerol-phosphate dehydratase |
| hisH | 1788334 | imidazole glycerol phosphate synthase, glutamine amidotransferase subunit with HisF |
| hisA | 87082028 | N-(5'-phospho-L-ribosyl-formimino)-5-amino-1- (5'-phosphoribosyl)-4-imidazolecarboxamide isomerase |
| hisF | 1788336 | imidazole glycerol phosphate synthase, catalytic subunit with HisH |
| hisI | 2367128 | fused phosphoribosyl-AMP cyclohydrolase/phosphoribosyl-ATP pyrophosphatase |
| ugd | 1788340 | UDP-glucose 6-dehydrogenase |
| gnd | 1788341 | gluconate-6-phosphate dehydrogenase, decarboxylating |
| rfbA | 1788351 | glucose-1-phosphate thymidylyltransferase |
| rfbB | 1788353 | dTDP-glucose 4,6 dehydratase, NAD(P)-binding |
| wza | 1788376 | lipoprotein required for capsular polysaccharide translocation through the outer membrane |
| yegH | 87082033 | fused predicted membrane protein/predicted membrane protein |
| asmA | 1788378 | predicted assembly protein |
| dcd | 1788379 | 2'-deoxycytidine 5'-triphosphate deaminase |
| udk | 87082034 | uridine/cytidine kinase |
| yegE | 1788381 | predicted diguanylate cyclase, GGDEF domain signalling protein |
| alkA | 1788383 | 3-methyl-adenine DNA glycosylase II |
| yegD | 87082035 | predicted chaperone |
| yegI | 1788385 | conserved protein |
| yegK | 1788387 | predicted protein |
| yegL | 1788388 | conserved protein |
| baeS | 1788393 | sensory histidine kinase in two-component regulatory system with BaeR |
| baeR | 1788394 | DNA-binding response regulator in two-component regulatory system with BaeS |
| yegP | 87082037 | predicted protein |
| yegQ | 1788397 | predicted peptidase |
| yegR | 87082038 | predicted protein |
| yegU | 1788416 | predicted hydrolase |
| yegV | 1788417 | predicted kinase |
| yegW | 1788418 | predicted DNA-binding transcriptional regulator |
| yegX | 87082043 | predicted hydrolase |
| thiD | 1788420 | bifunctional hydroxy-methylpyrimidine kinase/ hydroxy-phosphomethylpyrimidine kinase |
| thiM | 1788421 | hydoxyethylthiazole kinase |
| mrp | 87082045 | antiporter inner membrane protein |
| metG | 1788432 | methionyl-tRNA synthetase |
| yehT | 87082052 | predicted response regulator in two-component system withYehU |
| yehU | 1788446 | predicted sensory kinase in two-component system with YehT |
| yohO | 87082053 | predicted protein |
| yehW | 1788449 | predicted transporter subunit: membrane component of ABC superfamily |
| yehX | 1788450 | predicted transporter subunit: ATP-binding component of ABC superfamily |
| yehY | 1788451 | predicted transporter subunit: membrane component of ABC superfamily |
| osmF | 1788452 | predicted transporter subunit: periplasmic-binding component of ABC superfamily |
| bglX | 1788453 | beta-D-glucoside glucohydrolase, periplasmic |
| dld | 1788454 | D-lactate dehydrogenase, FAD-binding, NADH independent |
| pbpG | 87082054 | D-alanyl-D-alanine endopeptidase |
| yohC | 87082055 | predicted inner membrane protein |
| yohD | 87082056 | conserved inner membrane protein |
| yohF | 1788459 | predicted oxidoreductase with NAD(P)-binding Rossmann-fold domain |
| yohJ | 1788463 | conserved inner membrane protein |
| yohK | 1788464 | predicted inner membrane protein |
| cdd | 1788465 | cytidine/deoxycytidine deaminase |
| sanA | 1788466 | predicted protein |
| yeiS | 1788467 | predicted inner membrane protein |
| yeiT | 1788468 | Dihydropyrimidine dehydrogenase, NADH-dependent, subunit A |
| mglC | 1788471 | methyl-galactoside transporter subunit |
| mglB | 1788473 | methyl-galactoside transporter subunit |
| galS | 1788474 | DNA-binding transcriptional repressor |
| yeiB | 1788475 | conserved inner membrane protein |
| folE | 1788476 | GTP cyclohydrolase I |
| yeiG | 1788477 | predicted esterase |
| cirA | 1788478 | ferric iron-catecholate outer membrane transporter |
| lysP | 1788480 | lysine transporter |
| yeiE | 1788481 | predicted DNA-binding transcriptional regulator |
| yeiH | 1788482 | conserved inner membrane protein |
| nfo | 1788483 | endonuclease IV with intrinsic 3'-5' exonuclease activity |
| yeiI | 1788484 | predicted kinase |
| nupX | 1788485 | predicted nucleoside transporter |
| psuT | 1788488 | predicted nucleoside transporter |
| fruA | 1788492 | fused fructose-specific PTS enzymes: IIBcomponent/IIC components |
| fruK | 1788493 | fructose-1-phosphate kinase |
| fruB | 1788494 | fused fructose-specific PTS enzymes: IIA component/HPr component |
| yeiW | 87082060 | conserved protein |
| yeiP | 87082061 | predicted elongtion factor |
| yeiQ | 1788497 | predicted dehydrogenase, NAD-dependent |
| yeiR | 1788499 | predicted enzyme |
| lpxT | 87082062 | undecaprenyl pyrophosphate phosphatase |
| spr | 1788501 | predicted peptidase, outer membrane lipoprotein |
| rtn | 1788502 | conserved protein |
| yejA | 87082063 | predicted oligopeptide transporter subunit |
| yejB | 1788504 | predicted oligopeptide transporter subunit |
| yejE | 1788505 | predicted oligopeptide transporter subunit |
| yejF | 1788506 | fused predicted oligopeptide transporter subunits of ABC superfamilly: ATP-binding components |
| yejG | 1788507 | predicted protein |
| bcr | 1788509 | bicyclomycin/multidrug efflux system |
| rsuA | 1788510 | 16S rRNA pseudouridylate 516 synthase |
| yejH | 1788511 | predicted ATP-dependet helicase |
| rplY | 1788512 | 50S ribosomal subunit protein L25 |
| yejK | 1788513 | nucleotide associated protein |
| yejL | 1788514 | conserved protein |
| yejM | 1788515 | predicted hydrolase, inner membrane |
| narP | 1788521 | DNA-binding response regulator in two-component regulatory system with NarQ or NarX |
| ccmH | 1788522 | heme lyase, CcmH subunit |
| ccmG | 1788523 | periplasmic thioredoxin of cytochrome c-type biogenesis |
| ccmF | 1788524 | heme lyase, CcmF subunit |
| ccmE | 1788525 | periplasmic heme chaperone |
| ccmD | 1788526 | cytochrome c biogenesis protein |
| ccmC | 1788527 | heme exporter subunit |
| ccmB | 1788528 | heme exporter subunit |
| ccmA | 87082064 | heme exporter subunit |
| napC | 1788530 | nitrate reductase, cytochrome c-type, periplasmic |
| napB | 87082065 | nitrate reductase, small, cytochrome C550 subunit, periplasmic |
| napH | 1788532 | ferredoxin-type protein essential for electron transfer from ubiquinol to periplasmic nitrate reductase (NapAB) |
| napG | 1788533 | ferredoxin-type protein essential for electron transfer from ubiquinol to periplasmic nitrate reductase (NapAB) |
| napA | 1788534 | nitrate reductase, periplasmic, large subunit |
| napD | 1788535 | assembly protein for periplasmic nitrate reductase |
| napF | 1788536 | ferredoxin-type protein, predicted role in electron transfer to periplasmic nitrate reductase (NapA) |
| yojO | 145693156 | hypothetical protein |
| mqo | 1788539 | malate dehydrogenase, FAD/NAD(P)-binding domain |
| yojI | 1788540 | fused predicted multidrug transport subunits of ABC superfamily: membrane component/ATP-binding component |
| alkB | 1788541 | oxidative demethylase of N1-methyladenine or N3-methylcytosine DNA lesions |
| ada | 1788542 | fused DNA-binding transcriptional dual regulator/O6-methylguanine-DNA methyltransferase |
| apbE | 1788543 | predicted thiamine biosynthesis lipoprotein |
| ompC | 1788544 | outer membrane porin protein C |
| rcsD | 1788545 | phosphotransfer intermediate protein in two-component regulatory system with RcsBC |
| rcsB | 1788546 | DNA-binding response regulator in two-component regulatory system with RcsC and YojN |
| rcsC | 145693157 | hybrid sensory kinase in two-component regulatory system with RcsB and YojN |
| yfaP | 1788556 | conserved protein |
| yfaQ | 1788557 | predicted protein |
| gyrA | 1788562 | DNA gyrase (type II topoisomerase), subunit A |
| ubiG | 1788564 | bifunctional 3-demethylubiquinone-9 3-methyltransferase/ 2-octaprenyl-6-hydroxy phenol methylase |
| ypaB | 145693158 | hypothetical protein |
| nrdA | 2367133 | ribonucleoside diphosphate reductase 1, alpha subunit |
| nrdB | 1788567 | ribonucleoside diphosphate reductase 1, beta subunit, ferritin-like |
| yfaE | 1788568 | predicted 2Fe-2S cluster-containing protein |
| inaA | 1788569 | conserved protein |
| glpQ | 1788572 | periplasmic glycerophosphodiester phosphodiesterase |
| glpT | 1788573 | sn-glycerol-3-phosphate transporter |
| glpA | 1788574 | sn-glycerol-3-phosphate dehydrogenase (anaerobic), large subunit, FAD/NAD(P)-binding |
| glpB | 1788575 | sn-glycerol-3-phosphate dehydrogenase (anaerobic), membrane anchor subunit |
| glpC | 1788576 | sn-glycerol-3-phosphate dehydrogenase (anaerobic), small subunit |
| yfaD | 1788577 | conserved protein |
| ypaA | 87082070 | predicted protein |
| arnC | 1788588 | undecaprenyl phosphate-L-Ara4FN transferase |
| arnA | 1788589 | fused UDP-L-Ara4N formyltransferase/UDP-GlcA C-4'-decarboxylase |
| arnD | 1788590 | conserved protein |
| arnT | 1788591 | 4-amino-4-deoxy-L-arabinose transferase |
| arnE | 87082074 | conserved protein |
| arnF | 87082075 | predicted inner membrane protein |
| pmrD | 87082076 | polymyxin resistance protein B |
| menE | 1788595 | o-succinylbenzoate-CoA ligase |
| menC | 1788596 | o-succinylbenzoyl-CoA synthase |
| menB | 1788597 | dihydroxynaphthoic acid synthetase |
| yfbB | 1788598 | predicted peptidase |
| menD | 1788599 | bifunctional 2-oxoglutarate decarboxylase/ SHCHC synthase |
| menF | 87082077 | isochorismate synthase 2 |
| elaB | 1788601 | conserved protein |
| elaA | 1788602 | predicted acyltransferase with acyl-CoA N-acyltransferase domain |
| rbn | 87082078 | binuclear zinc phosphodiesterase |
| nuoN | 145693160 | NADH:ubiquinone oxidoreductase, membrane subunit N |
| nuoM | 1788613 | NADH:ubiquinone oxidoreductase, membrane subunit M |
| nuoL | 1788614 | NADH:ubiquinone oxidoreductase, membrane subunit L |
| nuoK | 1788615 | NADH:ubiquinone oxidoreductase, membrane subunit K |
| nuoJ | 1788616 | NADH:ubiquinone oxidoreductase, membrane subunit J |
| nuoI | 1788617 | NADH:ubiquinone oxidoreductase, chain I |
| nuoH | 1788618 | NADH:ubiquinone oxidoreductase, membrane subunit H |
| nuoG | 145693161 | NADH:ubiquinone oxidoreductase, chain G |
| nuoF | 1788620 | NADH:ubiquinone oxidoreductase, chain F |
| nuoE | 1788621 | NADH:ubiquinone oxidoreductase, chain E |
| nuoC | 145693162 | NADH:ubiquinone oxidoreductase, chain C,D |
| nuoB | 1788624 | NADH:ubiquinone oxidoreductase, chain B |
| nuoA | 1788625 | NADH:ubiquinone oxidoreductase, membrane subunit A |
| lrhA | 1788626 | DNA-binding transcriptional repressor of flagellar, motility and chemotaxis genes |
| yfbQ | 1788627 | predicted aminotransferase |
| yfbR | 1788628 | deoxyribonucleoside 5'-monophosphatase |
| yfbS | 1788629 | predicted transporter |
| yfbT | 87082080 | predicted hydrolase or phosphatase |
| yfbU | 87082081 | conserved protein |
| yfbV | 1788632 | conserved inner membrane protein |
| ackA | 1788633 | acetate kinase A and propionate kinase 2 |
| pta | 1788635 | phosphate acetyltransferase |
| yfcC | 87082082 | predicted inner membrane protein |
| yfcD | 1788637 | predicted NUDIX hydrolase |
| yfcE | 1788638 | predicted phosphatase |
| yfcF | 1788639 | predicted enzyme |
| yfcG | 1788640 | predicted glutathione S-transferase |
| yfcH | 1788642 | conserved protein with NAD(P)-binding Rossmann-fold domain |
| hisP | 1788644 | histidine/lysine/arginine/ornithine transporter subunit |
| hisM | 1788645 | histidine/lysine/arginine/ornithine transporter subunit |
| hisQ | 1788646 | histidine/lysine/arginine/ornithine transporter subunit |
| hisJ | 1788648 | histidine/lysine/arginine/ornithine transporter subunit |
| argT | 1788649 | lysine/arginine/ornithine transporter subunit |
| ubiX | 1788650 | 3-octaprenyl-4-hydroxybenzoate carboxy-lyase |
| purF | 1788651 | amidophosphoribosyltransferase |
| cvpA | 1788652 | membrane protein required for colicin V production |
| dedD | 87082083 | conserved protein |
| folC | 1788654 | bifunctional folylpolyglutamate synthase/ dihydrofolate synthase |
| accD | 1788655 | acetyl-CoA carboxylase, beta (carboxyltranferase) subunit |
| dedA | 1788656 | conserved inner membrane protein |
| truA | 1788657 | pseudouridylate synthase I |
| usg | 1788658 | predicted semialdehyde dehydrogenase |
| pdxB | 1788660 | erythronate-4-phosphate dehydrogenase |
| flk | 1788661 | predicted flagella assembly protein |
| fabB | 1788663 | 3-oxoacyl-[acyl-carrier-protein] synthase I |
| mnmC | 87082084 | fused 5-methylaminomethyl-2-thiouridine-forming enzyme methyltransferase/FAD-dependent demodification enzyme |
| yfcL | 1788665 | predicted protein |
| yfcM | 1788666 | conserved protein |
| yfcA | 1788667 | conserved inner membrane protein |
| mepA | 1788668 | murein DD-endopeptidase |
| aroC | 1788669 | chorismate synthase |
| prmB | 87082085 | N5-glutamine methyltransferase |
| yfcN | 1788671 | conserved protein |
| yfcS | 1788677 | predicted periplasmic pilus chaperone |
| yfcV | 1788680 | predicted fimbrial-like adhesin protein |
| sixA | 1788681 | phosphohistidine phosphatase |
| fadJ | 1788682 | fused enoyl-CoA hydratase and epimerase and isomerase/3-hydroxyacyl-CoA dehydrogenase |
| fadI | 1788683 | beta-ketoacyl-CoA thiolase, anaerobic, subunit |
| yfcZ | 87082087 | conserved protein |
| fadL | 145693163 | long-chain fatty acid outer membrane transporter |
| vacJ | 1788688 | predicted lipoprotein |
| yfdC | 1788689 | predicted inner membrane protein |
| emrY | 1788710 | predicted multidrug efflux system |
| oxc | 1788716 | predicted oxalyl-CoA decarboxylase |
| frc | 1788717 | formyl-CoA transferase, NAD(P)-binding |
| yfdX | 1788719 | predicted protein |
| ypdI | 2367134 | predicted lipoprotein involved in colanic acid biosynthesis |
| yfdY | 1788720 | predicted inner membrane protein |
| lpxP | 87082094 | palmitoleoyl-acyl carrier protein (ACP)-dependent acyltransferase |
| yfdZ | 1788722 | prediected aminotransferase, PLP-dependent |
| ypdB | 1788724 | predicted response regulator in two-component system withYpdA |
| ypdC | 1788725 | predicted DNA-binding protein |
| fryA | 1788726 | fused predicted PTS enzymes: Hpr component/enzyme I component/enzyme IIA component |
| ypdE | 1788727 | predicted peptidase |
| ypdF | 1788728 | predicted peptidase |
| fryC | 1788729 | predicted enzyme IIC component of PTS |
| fryB | 1788730 | predicted enzyme IIB component of PTS |
| glk | 1788732 | glucokinase |
| mntH | 1788736 | manganese/divalent cation transporter |
| nupC | 1788737 | nucleoside (except guanosine) transporter |
| yfeA | 87082096 | predicted diguanylate cyclase |
| yfeC | 87082097 | predicted DNA-binding transcriptional regulator |
| yfeD | 145693165 | predicted DNA-binding transcriptional regulator |
| gltX | 1788743 | glutamyl-tRNA synthetase |
| ptsI | 1788756 | PEP-protein phosphotransferase of PTS system (enzyme I) |
| crr | 1788757 | glucose-specific enzyme IIA component of PTS |
| pdxK | 1788758 | pyridoxal-pyridoxamine kinase/hydroxymethylpyrimidine kinase |
| yfeK | 1788759 | predicted protein |
| cysM | 2367138 | cysteine synthase B (O-acetylserine sulfhydrolase B) |
| cysA | 1788761 | sulfate/thiosulfate transporter subunit |
| cysW | 87082099 | sulfate/thiosulfate transporter subunit |
| cysU | 1788764 | sulfate/thiosulfate transporter subunit |
| cysP | 1788765 | thiosulfate transporter subunit |
| ucpA | 87082100 | predicted oxidoredutase, sulfate metabolism protein |
| murQ | 1788768 | predicted PTS component |
| murP | 1788769 | fused predicted PTS enzymes: IIB component/IIC component |
| yfeX | 87082102 | conserved protein |
| yfeY | 1788772 | predicted protein |
| yfeZ | 87082103 | predicted inner membrane protein |
| ypeA | 87082104 | predicted acyltransferase with acyl-CoA N-acyltransferase domain |
| amiA | 1788776 | N-acetylmuramoyl-l-alanine amidase I |
| hemF | 1788777 | coproporphyrinogen III oxidase |
| eutR | 1788778 | predicted DNA-binding transcriptional regulator |
| eutK | 87082105 | predicted carboxysome structural protein with predicted role in ethanolamine utilization |
| eutL | 1788780 | predicted carboxysome structural protein with predicted role in ethanolamine utilization |
| maeB | 1788806 | fused malic enzyme predicted oxidoreductase/predicted phosphotransacetylase |
| talA | 1788807 | transaldolase A |
| tktB | 1788808 | transketolase 2, thiamin-binding |
| ypfG | 1788809 | predicted protein |
| nudK | 1788810 | predicted NUDIX hydrolase |
| aegA | 1788811 | fused predicted oxidoreductase: FeS binding subunit/NAD/FAD-binding subunit |
| narQ | 1788812 | sensory histidine kinase in two-component regulatory system with NarP (NarL) |
| acrD | 1788814 | aminoglycoside/multidrug efflux system |
| ypfM | 145693170 | hypothetical protein |
| yffB | 1788815 | conserved protein |
| dapE | 1788816 | N-succinyl-diaminopimelate deacylase |
| ypfN | 87082110 | predicted protein |
| ypfH | 87082111 | predicted hydrolase |
| tmcA | 1788818 | Elongator methionine tRNA (ac4C34) acetyltransferase |
| ypfJ | 1788819 | conserved protein |
| purC | 1788820 | phosphoribosylaminoimidazole-succinocarboxamide synthetase |
| bamC | 87082112 | lipoprotein |
| dapA | 1788823 | dihydrodipicolinate synthase |
| gcvR | 87082113 | DNA-binding transcriptional repressor, regulatory protein accessory to GcvA |
| bcp | 1788825 | thiol peroxidase, thioredoxin-dependent |
| hyfG | 1788832 | hydrogenase 4, subunit |
| hyfI | 1788834 | hydrogenase 4, Fe-S subunit |
| yfgO | 1788838 | predicted inner membrane protein |
| yfgC | 1788840 | predicted peptidase |
| yfgD | 1788841 | predicted oxidoreductase |
| hda | 1788842 | ATPase regulatory factor involved in DnaA inactivation |
| uraA | 1788843 | uracil transporter |
| upp | 87082118 | uracil phosphoribosyltransferase |
| purM | 1788845 | phosphoribosylaminoimidazole synthetase |
| purN | 1788846 | phosphoribosylglycinamide formyltransferase 1 |
| ppk | 1788847 | polyphosphate kinase, component of RNA degradosome |
| ppx | 1788848 | exopolyphosphatase |
| guaA | 1788854 | GMP synthetase (glutamine aminotransferase) |
| guaB | 1788855 | IMP dehydrogenase |
| xseA | 1788856 | exonuclease VII, large subunit |
| yfgJ | 87082119 | predicted protein |
| der | 87082120 | predicted GTP-binding protein |
| bamB | 1788859 | protein assembly complex, lipoprotein component |
| yfgM | 1788860 | conserved protein |
| hisS | 1788861 | histidyl tRNA synthetase |
| ispG | 1788863 | 1-hydroxy-2-methyl-2-(E)-butenyl 4-diphosphate synthase |
| rodZ | 1788864 | conserved protein |
| rlmN | 1788865 | 23S rRNA m(2)A2503 methyltransferase, SAM-dependen |
| ndk | 1788866 | multifunctional nucleoside diphosphate kinase and apyrimidinic endonuclease and 3'-phosphodiesterase |
| pbpC | 1788867 | fused transglycosylase/transpeptidase |
| sseB | 87082122 | rhodanase-like enzyme, sulfur transfer from thiosulfate |
| pepB | 87082123 | aminopeptidase B |
| iscX | 1788873 | conserved protein |
| fdx | 1788874 | [2Fe-2S] ferredoxin |
| hscA | 1788875 | DnaK-like molecular chaperone specific for IscU |
| hscB | 1788876 | DnaJ-like molecular chaperone specific for IscU |
| iscA | 1788877 | FeS cluster assembly protein |
| iscS | 48994898 | cysteine desulfurase (tRNA sulfurtransferase), PLP-dependent |
| iscR | 1788880 | DNA-binding transcriptional repressor |
| trmJ | 1788881 | tRNA mC32/mU32 methyltransferase, SAM-dependent |
| suhB | 1788882 | inositol monophosphatase |
| yfhR | 1788884 | predicted peptidase |
| csiE | 87082124 | stationary phase inducible protein |
| hcaT | 1788886 | predicted 3-phenylpropionic transporter |
| yphG | 87082126 | conserved protein |
| yphH | 87082127 | predicted DNA-binding transcriptional regulator |
| glyA | 1788902 | serine hydroxymethyltransferase |
| hmp | 1788903 | fused nitric oxide dioxygenase/dihydropteridine reductase 2 |
| glnB | 1788904 | regulatory protein P-II for glutamine synthetase |
| qseF | 1788905 | predicted DNA-binding response regulator in two-component system |
| qseG | 1788906 | conserved protein |
| qseE | 87082128 | predicted sensory kinase in two-component system |
| purL | 48994899 | phosphoribosylformyl-glycineamide synthetase |
| mltF | 171474010 | predicted periplasmic binding protein/transglycosylase |
| tadA | 145693172 | tRNA-specific adenosine deaminase |
| yfhB | 87082129 | conserved protein |
| yfhH | 87082130 | predicted DNA-binding transcriptional regulator |
| yfhL | 1788914 | predicted 4Fe-4S cluster-containing protein |
| acpS | 1788916 | holo-[acyl-carrier-protein] synthase 1 |
| pdxJ | 1788917 | pyridoxine 5'-phosphate synthase |
| recO | 2367140 | gap repair protein |
| era | 1788919 | membrane-associated, 16S rRNA-binding GTPase |
| rnc | 1788920 | RNase III |
| lepB | 1788921 | leader peptidase (signal peptidase I) |
| lepA | 1788922 | GTP-binding membrane protein |
| rseC | 1788923 | RseC protein involved in reduction of the SoxR iron-sulfur cluster |
| rseB | 1788924 | anti-sigma factor |
| rseA | 1788925 | anti-sigma factor |
| rpoE | 1788926 | RNA polymerase, sigma 24 (sigma E) factor |
| yfiC | 87082131 | predicted S-adenosyl-L-methionine-dependent methyltransferase |
| srmB | 1788930 | ATP-dependent RNA helicase |
| yfiE | 87082132 | predicted DNA-binding transcriptional regulator |
| yfiD | 1788933 | pyruvate formate lyase subunit |
| ung | 1788934 | uracil-DNA-glycosylase |
| yfiF | 1788935 | predicted methyltransferase |
| trxC | 1788936 | thioredoxin 2 |
| yfiP | 87082133 | conserved protein |
| yfiQ | 1788938 | fused predicted acyl-CoA synthetase: NAD(P)-binding subunit/ATP-binding subunit |
| pssA | 87082134 | phosphatidylserine synthase (CDP-diacylglycerol-serine O-phosphatidyltransferase) |
| yfiM | 145693173 | predicted protein |
| kgtP | 1788942 | alpha-ketoglutarate transporter |
| clpB | 1788943 | protein disaggregation chaperone |
| yfiH | 1788945 | conserved protein |
| rluD | 1788946 | 23S rRNA pseudouridine synthase |
| bamD | 1788947 | predicted lipoprotein |
| raiA | 1788949 | cold shock protein associated with 30S ribosomal subunit |
| pheA | 1788951 | fused chorismate mutase P/prephenate dehydratase |
| tyrA | 1788952 | fused chorismate mutase T/prephenate dehydrogenase |
| aroF | 1788953 | 3-deoxy-D-arabino-heptulosonate-7-phosphate synthase, tyrosine-repressible |
| yfiL | 87082135 | predicted protein |
| yfiB | 1788957 | predicted outer membrane lipoprotein |
| rplS | 1788958 | 50S ribosomal subunit protein L19 |
| trmD | 1788959 | tRNA m(1)G37 methyltransferase, SAM-dependent |
| rimM | 87082136 | 16S rRNA processing protein |
| rpsP | 1788961 | 30S ribosomal subunit protein S16 |
| yfjD | 145693175 | predicted inner membrane protein |
| nadK | 1788968 | NAD kinase |
| recN | 48994901 | recombination and repair protein |
| smpA | 87082138 | small membrane lipoprotein |
| yfjF | 87082139 | predicted protein |
| yfjG | 1788972 | conserved protein |
| smpB | 1788973 | trans-translation protein |
| gabT | 1789016 | 4-aminobutyrate aminotransferase, PLP-dependent |
| gabP | 1789017 | gamma-aminobutyrate transporter |
| csiR | 87082149 | DNA-binding transcriptional dual regulator |
| ygaU | 1789019 | predicted protein |
| yqaE | 1789020 | predicted membrane protein |
| ygaV | 1789021 | predicted DNA-binding transcriptional regulator |
| ygaP | 1789022 | predicted inner membrane protein with hydrolase activity |
| stpA | 1789023 | DNA binding protein, nucleoid-associated |
| ygaW | 1789025 | predicted inner membrane protein |
| ygaC | 1789026 | predicted protein |
| ygaM | 1789027 | predicted protein |
| nrdH | 1789028 | glutaredoxin-like protein |
| nrdI | 1789029 | protein that stimulates ribonucleotide reduction |
| nrdE | 1789030 | ribonucleoside-diphosphate reductase 2, alpha subunit |
| nrdF | 1789031 | ribonucleoside-diphosphate reductase 2, beta subunit, ferritin-like |
| proV | 1789032 | glycine betaine transporter subunit |
| proW | 1789033 | glycine betaine transporter subunit |
| proX | 1789034 | glycine betaine transporter subunit |
| ygaZ | 1789038 | predicted transporter |
| ygaH | 1789039 | predicted inner membrane protein |
| mprA | 1789040 | DNA-binding transcriptional repressor of microcin B17 synthesis and multidrug efflux |
| emrA | 1789041 | multidrug efflux system |
| emrB | 1789042 | multidrug efflux system protein |
| luxS | 1789043 | S-ribosylhomocysteinase |
| gshA | 1789044 | gamma-glutamate-cysteine ligase |
| yqaA | 1789045 | conserved inner membrane protein |
| yqaB | 1789046 | predicted hydrolase |
| csrA | 1789047 | pleiotropic regulatory protein for carbon source metabolism |
| alaS | 1789048 | alanyl-tRNA synthetase |
| recX | 1789050 | regulatory protein for RecA |
| recA | 1789051 | DNA strand exchange and recombination protein with protease and nuclease activity |
| ygaD | 1789052 | conserved protein |
| mltB | 1789053 | membrane-bound lytic murein transglycosylase B |
| gutQ | 87082151 | predicted phosphosugar-binding protein |
| norR | 87082152 | DNA-binding transcriptional activator |
| norV | 1789064 | flavorubredoxin oxidoreductase |
| norW | 1789065 | NADH:flavorubredoxin oxidoreductase |
| hypF | 2367152 | carbamoyl phosphate phosphatase and maturation protein for [NiFe] hydrogenases |
| hycG | 1789074 | hydrogenase 3 and formate hydrogenase complex, HycG subunit |
| hycE | 1789076 | hydrogenase 3, large subunit |
| hycD | 1789077 | hydrogenase 3, membrane subunit |
| hypA | 1789081 | protein involved in nickel insertion into hydrogenases 3 |
| hypB | 1789082 | GTP hydrolase involved in nickel liganding into hydrogenases |
| hypC | 1789083 | protein required for maturation of hydrogenases 1 and 3 |
| hypD | 1789084 | protein required for maturation of hydrogenases |
| hypE | 1789085 | carbamoyl phosphate phosphatase, hydrogenase 3 maturation protein |
| fhlA | 1789087 | DNA-binding transcriptional activator |
| ygbA | 1789088 | predicted protein |
| mutS | 1789089 | methyl-directed mismatch repair protein |
| rpoS | 1789098 | RNA polymerase, sigma S (sigma 38) factor |
| nlpD | 1789099 | predicted outer membrane lipoprotein |
| pcm | 1789100 | L-isoaspartate protein carboxylmethyltransferase type II |
| surE | 1789101 | broad specificity 5'(3')-nucleotidase and polyphosphatase |
| truD | 1789102 | tRNA(Glu) U13 pseudouridine synthase |
| ispF | 1789103 | 2C-methyl-D-erythritol 2,4-cyclodiphosphate synthase |
| ispD | 1789104 | 4-diphosphocytidyl-2C-methyl-D-erythritol synthase |
| ftsB | 1789105 | cell division protein |
| ygbE | 1789106 | conserved inner membrane protein |
| cysC | 1789107 | adenosine 5'-phosphosulfate kinase |
| cysN | 1789108 | sulfate adenylyltransferase, subunit 1 |
| cysD | 1789109 | sulfate adenylyltransferase, subunit 2 |
| iap | 1789111 | aminopeptidase in alkaline phosphatase isozyme conversion |
| cysH | 1789121 | 3'-phosphoadenosine 5'-phosphosulfate reductase |
| cysI | 1789122 | sulfite reductase, beta subunit, NAD(P)-binding, heme-binding |
| cysJ | 1789123 | sulfite reductase, alpha subunit, flavoprotein |
| queD | 1789124 | 6-pyruvoyl tetrahydrobiopterin synthase (PTPS) |
| ygcF | 1789139 | conserved protein |
| pyrG | 1789142 | CTP synthetase |
| relA | 1789147 | (p)ppGpp synthetase I/GTP pyrophosphokinase |
| rumA | 1789148 | 23S rRNA m(5)U1939 methyltransferase, SAM-dependent |
| gudD | 1789150 | (D)-glucarate dehydratase 1 |
| gudX | 1789151 | predicted glucarate dehydratase |
| gudP | 1789152 | predicted D-glucarate transporter |
| yqcA | 1789154 | predicted flavoprotein |
| truC | 1789155 | tRNA U65 pseudouridine synthase |
| yqcC | 1789156 | conserved protein |
| syd | 1789157 | predicted protein |
| queF | 1789158 | 7-cyano-7-deazaguanine reductase (NADPH-dependent) |
| ygdH | 1789159 | conserved protein |
| sdaC | 1789160 | predicted serine transporter |
| sdaB | 1789161 | L-serine deaminase II |
| ygdG | 1789162 | Ssb-binding protein, misidentified as ExoIX |
| fucO | 1789163 | L-1,2-propanediol oxidoreductase |
| fucA | 1789164 | L-fuculose-1-phosphate aldolase |
| fucI | 1789167 | L-fucose isomerase |
| fucK | 1789168 | L-fuculokinase |
| fucU | 1789169 | L-fucose mutarotase |
| fucR | 1789170 | DNA-binding transcriptional activator |
| ygdE | 1789171 | predicted methyltransferase |
| ygdD | 1789172 | conserved inner membrane protein |
| ygdI | 87082162 | predicted protein |
| csdA | 1789175 | cysteine sulfinate desulfinase |
| csdE | 1789176 | predicted Fe-S metabolism protein |
| ygdL | 1789177 | conserved protein |
| mltA | 1789179 | membrane-bound lytic murein transglycosylase A |
| amiC | 87082163 | N-acetylmuramoyl-L-alanine amidase |
| argA | 1789181 | fused acetylglutamate kinase homolog (inactive)/amino acid N-acetyltransferase |
| recD | 1789182 | exonuclease V (RecBCD complex), alpha chain |
| recB | 1789183 | exonuclease V (RecBCD complex), beta subunit |
| ptrA | 2367164 | protease III |
| recC | 1789186 | exonuclease V (RecBCD complex), gamma chain |
| ppdC | 1789187 | predicted protein |
| ygdB | 87082164 | predicted protein |
| ppdA | 1789190 | conserved protein |
| thyA | 1789191 | thymidylate synthetase |
| lgt | 1789192 | phosphatidylglycerol-prolipoprotein diacylglyceryl transferase |
| ptsP | 1789193 | fused PTS enzyme: PEP-protein phosphotransferase (enzyme I)/GAF domain containing protein |
| rppH | 1789194 | nucleotide hydrolase |
| mutH | 1789196 | methyl-directed mismatch repair protein |
| ygdQ | 1789197 | predicted inner membrane protein |
| ygdR | 1789198 | predicted protein |
| tas | 1789199 | predicted oxidoreductase, NADP(H)-dependent aldo-keto reductase |
| lplT | 1789200 | predicted inner membrane protein |
| aas | 1789201 | fused 2-acylglycerophospho-ethanolamine acyl transferase/acyl-acyl carrier protein synthetase |
| galR | 1789202 | DNA-binding transcriptional repressor |
| lysA | 1789203 | diaminopimelate decarboxylase, PLP-binding |
| lysR | 1789204 | DNA-binding transcriptional dual regulator |
| ygeA | 1789205 | predicted racemase |
| araE | 1789207 | arabinose transporter |
| idi | 1789255 | isopentenyl diphosphate isomerase |
| lysS | 1789256 | lysine tRNA synthetase, constitutive |
| prfB | 2367172 | peptide chain release factor RF-2 |
| recJ | 1789259 | ssDNA exonuclease, 5' --> 3'-specific |
| dsbC | 1789260 | protein disulfide isomerase II |
| xerD | 1789261 | site-specific tyrosine recombinase |
| fldB | 1789262 | flavodoxin 2 |
| ygfX | 1789263 | predicted protein |
| ygfY | 1789264 | conserved protein |
| ygfZ | 1789265 | predicted folate-dependent regulatory protein |
| yqfA | 1789266 | predicted oxidoreductase, inner membrane subunit |
| yqfB | 1789267 | conserved protein |
| bglA | 2367174 | 6-phospho-beta-glucosidase A |
| gcvP | 1789269 | glycine decarboxylase, PLP-dependent, subunit (protein P) of glycine cleavage complex |
| gcvH | 1789271 | glycine cleavage complex lipoylprotein |
| gcvT | 1789272 | aminomethyltransferase, tetrahydrofolate-dependent, subunit (T protein) of glycine cleavage complex |
| visC | 1789273 | predicted oxidoreductase with FAD/NAD(P)-binding domain |
| ubiH | 1789274 | 2-octaprenyl-6-methoxyphenol hydroxylase, FAD/NAD(P)-binding |
| pepP | 1789275 | proline aminopeptidase P II |
| ygfB | 87082182 | predicted protein |
| ygfA | 1789278 | predicted ligase |
| serA | 1789279 | D-3-phosphoglycerate dehydrogenase |
| rpiA | 1789280 | ribose 5-phosphate isomerase, constitutive |
| argP | 1789283 | DNA-binding transcriptional activator, replication initiation inhibitor |
| yggE | 1789289 | conserved protein |
| argO | 1789290 | arginine transporter |
| mscS | 1789291 | mechanosensitive channel |
| fbaA | 1789293 | fructose-bisphosphate aldolase, class II |
| pgk | 1789294 | phosphoglycerate kinase |
| epd | 1789295 | D-erythrose 4-phosphate dehydrogenase |
| yggC | 1789296 | conserved protein with nucleoside triphosphate hydrolase domain |
| tktA | 48994911 | transketolase 1, thiamin-binding |
| yggG | 87082185 | predicted peptidase |
| speB | 1789306 | agmatinase |
| speA | 1789307 | biosynthetic arginine decarboxylase, PLP-binding |
| yqgB | 145693178 | predicted protein |
| yqgC | 1789309 | predicted protein |
| yqgD | 1789310 | predicted inner membrane protein |
| metK | 1789311 | methionine adenosyltransferase 1 |
| galP | 1789312 | D-galactose transporter |
| yggI | 1789313 | conserved protein |
| endA | 1789314 | DNA-specific endonuclease I |
| rsmE | 87082186 | 16S rRNA m(3)U1498 methyltransferase, SAM-dependent |
| gshB | 1789316 | glutathione synthetase |
| yqgE | 87082187 | predicted protein |
| yqgF | 1789318 | predicted Holliday junction resolvase |
| yggR | 87082188 | predicted transporter |
| yggS | 1789321 | predicted enzyme |
| yggT | 1789322 | predicted inner membrane protein |
| yggU | 87082189 | conserved protein |
| rdgB | 1789324 | dITP/XTP pyrophosphatase |
| yggW | 1789325 | predicted oxidoreductase |
| ansB | 1789327 | periplasmic L-asparaginase II |
| yggN | 1789328 | predicted protein |
| yggL | 87082190 | predicted protein |
| trmI | 1789330 | tRNA m(7)G46 methyltransferase, SAM-dependent |
| mutY | 1789331 | adenine DNA glycosylase |
| yggX | 1789332 | protein that protects iron-sulfur proteins against oxidative damage |
| mltC | 87082191 | membrane-bound lytic murein transglycosylase C |
| nupG | 87082192 | nucleoside transporter |
| speC | 87082193 | ornithine decarboxylase, constitutive |
| glcA | 1789347 | glycolate transporter |
| pitB | 1789360 | phosphate transporter |
| gsp | 1789361 | fused glutathionylspermidine amidase/glutathionylspermidine synthetase |
| yghU | 87082195 | predicted S-transferase |
| hybG | 1789364 | hydrogenase 2 accessory protein |
| hybF | 1789365 | protein involved with the maturation of hydrogenases 1 and 2 |
| hybE | 1789366 | hydrogenase 2-specific chaperone |
| hybD | 1789367 | predicted maturation element for hydrogenase 2 |
| hybC | 1789368 | hydrogenase 2, large subunit |
| hybB | 2367183 | predicted hydrogenase 2 cytochrome b type component |
| hybA | 1789370 | hydrogenase 2 4Fe-4S ferredoxin-type component |
| hybO | 1789371 | hydrogenase 2, small subunit |
| yghW | 1789372 | predicted protein |
| yqhA | 1789376 | conserved inner membrane protein |
| yghA | 1789378 | predicted glutathionylspermidine synthase, with NAD(P)-binding Rossmann-fold domain |
| exbD | 1789380 | membrane spanning protein in TonB-ExbB-ExbD complex |
| exbB | 1789381 | membrane spanning protein in TonB-ExbB-ExbD complex |
| metC | 1789383 | cystathionine beta-lyase, PLP-dependent |
| yghB | 1789384 | synthetic lethal with yqjA at high temperature |
| yqhC | 87082197 | predicted DNA-binding transcriptional regulator |
| yqhD | 1789386 | alcohol dehydrogenase, NAD(P)-dependent |
| dkgA | 87082198 | 2,5-diketo-D-gluconate reductase A |
| ftsP | 1789394 | repressor protein for FtsI |
| plsC | 1789395 | 1-acyl-sn-glycerol-3-phosphate acyltransferase |
| parC | 1789396 | DNA topoisomerase IV, subunit A |
| ygiV | 48994918 | predicted transcriptional regulator |
| ygiW | 1789401 | conserved protein |
| qseB | 1789402 | DNA-binding response regulator in two-component regulatory system with QseC |
| qseC | 1789403 | sensory histidine kinase in two-component regulatory system with QseB |
| mdaB | 1789406 | NADPH quinone reductase |
| ygiN | 1789407 | quinol monooxygenase |
| parE | 1789408 | DNA topoisomerase IV, subunit B |
| yqiA | 1789409 | predicted esterase |
| cpdA | 1789410 | cyclic 3',5'-adenosine monophosphate phosphodiesterase |
| yqiB | 1789411 | predicted dehydrogenase |
| nudF | 1789412 | ADP-ribose pyrophosphatase |
| tolC | 87082199 | transport channel |
| ygiB | 145693179 | conserved outer membrane protein |
| ygiC | 1789416 | predicted enzyme |
| ygiD | 1789417 | predicted dioxygenase |
| ribB | 1789420 | 3,4-dihydroxy-2-butanone-4-phosphate synthase |
| yqiC | 87082201 | conserved protein |
| rfaE | 1789432 | fused heptose 7-phosphate kinase/heptose 1-phosphate adenyltransferase |
| glnE | 1789433 | fused deadenylyltransferase/adenylyltransferase for glutamine synthetase |
| ygiF | 1789434 | predicted adenylate cyclase |
| ygiM | 1789435 | predicted signal transduction protein (SH3 domain) |
| cca | 1789436 | fused tRNA nucleotidyl transferase/2'3'-cyclic phosphodiesterase/2'nucleotidase and phosphatase |
| bacA | 1789437 | undecaprenyl pyrophosphate phosphatase |
| folB | 87082204 | bifunctional dihydroneopterin aldolase/dihydroneopterin triphosphate 2'-epimerase |
| plsY | 1789439 | conserved inner membrane protein |
| ttdA | 1789442 | L-tartrate dehydratase, alpha subunit |
| ttdB | 1789443 | L-tartrate dehydratase, beta subunit |
| ttdT | 1789444 | predicted tartrate:succinate antiporter |
| ygjD | 1789445 | predicted peptidase |
| dnaG | 1789447 | DNA primase |
| rpoD | 1789448 | RNA polymerase, sigma 70 (sigma D) factor |
| mug | 1789449 | G/U mismatch-specific DNA glycosylase |
| yqjH | 1789450 | predicted siderophore interacting protein |
| yqjI | 1789452 | predicted transcriptional regulator |
| aer | 1789453 | fused signal transducer for aerotaxis sensory component/methyl accepting chemotaxis component |
| patA | 145693181 | putrescine:2-oxoglutaric acid aminotransferase, PLP-dependent |
| ygjH | 1789455 | conserved protein |
| ebgR | 1789456 | DNA-binding transcriptional repressor |
| ebgA | 48994920 | cryptic beta-D-galactosidase, alpha subunit |
| ygjK | 1789462 | predicted glycosyl hydrolase |
| fadH | 1789463 | 2,4-dienoyl-CoA reductase, NADH and FMN-linked |
| rlmG | 87082206 | 23S rRNA mG1835 methyltransferase, SAM-dependent |
| ygjP | 87082207 | predicted metal dependent hydrolase |
| ygjQ | 1789468 | predicted thioredoxin-like |
| ygjR | 145693182 | predicted NAD(P)-binding dehydrogenase |
| alx | 48994922 | predicted inner membrane protein, part of terminus |
| sstT | 1789473 | sodium:serine/threonine symporter |
| ygjV | 1789474 | conserved inner membrane protein |
| uxaA | 1789475 | altronate hydrolase |
| uxaC | 2367192 | uronate isomerase |
| exuT | 2367193 | hexuronate transporter |
| exuR | 87082208 | DNA-binding transcriptional repressor |
| yqjA | 1789481 | synthetic lethal with yghB at high temperature |
| yqjB | 1789482 | conserved protein |
| yqjC | 87082209 | conserved protein |
| yqjD | 1789485 | conserved protein |
| yqjE | 1789486 | conserved inner membrane protein |
| yqjK | 1789487 | conserved protein |
| yqjG | 1789489 | predicted S-transferase |
| yhaH | 2367196 | predicted inner membrane protein |
| yhaJ | 1789492 | predicted DNA-binding transcriptional regulator |
| yhaK | 1789493 | predicted pirin-related protein |
| yhaL | 87082211 | predicted protein |
| yhaM | 48994923 | conserved protein |
| yhaO | 145693183 | predicted transporter |
| tdcG | 48994925 | L-serine dehydratase 3 |
| tdcF | 87082213 | predicted L-PSP (mRNA) endoribonuclease |
| tdcE | 48994926 | pyruvate formate-lyase 4/2-ketobutyrate formate-lyase |
| tdcD | 145693184 | propionate kinase/acetate kinase C, anaerobic |
| tdcC | 1789504 | L-threonine/L-serine transporter |
| tdcB | 1789505 | catabolic threonine dehydratase, PLP-dependent |
| tdcA | 1789506 | DNA-binding transcriptional activator |
| tdcR | 87082214 | DNA-binding transcriptional activator |
| garK | 145693185 | glycerate kinase I |
| garR | 145693186 | tartronate semialdehyde reductase |
| garL | 1789514 | alpha-dehydro-beta-deoxy-D-glucarate aldolase |
| garP | 1789515 | predicted (D)-galactarate transporter |
| yraL | 1789535 | predicted methyltransferase |
| yraM | 1789537 | conserved protein |
| yraN | 1789538 | conserved protein |
| diaA | 1789539 | DnaA initiator-associating factor for replication initiation |
| yraP | 1789540 | predicted protein |
| yraQ | 1789541 | predicted permease |
| yraR | 87082218 | predicted nucleoside-diphosphate-sugar epimerase |
| yhbP | 1789544 | conserved protein |
| yhbQ | 1789545 | predicted endonuclease |
| yhbS | 1789546 | predicted acyltransferase with acyl-CoA N-acyltransferase domain |
| yhbT | 1789547 | predicted lipid carrier protein |
| yhbU | 1789548 | predicted peptidase (collagenase-like) |
| yhbV | 87082220 | predicted protease |
| yhbW | 1789551 | predicted enzyme |
| mtr | 1789552 | tryptophan transporter of high affinity |
| deaD | 87082221 | ATP-dependent RNA helicase |
| nlpI | 1789554 | conserved protein |
| pnp | 145693187 | polynucleotide phosphorylase/polyadenylase |
| rpsO | 1789556 | 30S ribosomal subunit protein S15 |
| truB | 2367200 | tRNA U55 pseudouridine synthase |
| rbfA | 1789558 | 30s ribosome binding factor |
| infB | 1789559 | fused protein chain initiation factor 2, IF2: membrane protein/conserved protein |
| yhbC | 145693188 | conserved protein |
| argG | 1789563 | argininosuccinate synthetase |
| secG | 1789565 | preprotein translocase membrane subunit |
| glmM | 1789566 | phosphoglucosamine mutase |
| folP | 87082224 | 7,8-dihydropteroate synthase |
| ispB | 1789578 | octaprenyl diphosphate synthase |
| sfsB | 1789579 | DNA-binding transcriptional activator of maltose metabolism |
| murA | 1789580 | UDP-N-acetylglucosamine 1-carboxyvinyltransferase |
| yrbA | 87082226 | predicted DNA-binding transcriptional regulator |
| yrbB | 87082227 | predicted protein |
| yrbC | 1789583 | predicted ABC-type organic solvent transporter |
| yrbD | 1789584 | predicted ABC-type organic solvent transporter |
| yrbE | 1789585 | predicted toluene transporter subunit: membrane component of ABC superfamily |
| yrbF | 1789586 | predicted toluene transporter subunit: ATP-binding component of ABC superfamily |
| yrbG | 1789587 | predicted calcium/sodium:proton antiporter |
| kdsD | 1789588 | D-arabinose 5-phosphate isomerase |
| kdsC | 2367202 | 3-deoxy-D-manno-octulosonate 8-phosphate phosphatase |
| lptC | 1789591 | conserved protein |
| lptA | 1789592 | periplasmic LPS-binding protein |
| lptB | 1789593 | predicted transporter subunit: ATP-binding component of ABC superfamily |
| rpoN | 1789594 | RNA polymerase, sigma 54 (sigma N) factor |
| hpf | 1789595 | predicted ribosome-associated, sigma 54 modulation protein |
| ptsN | 1789597 | sugar-specific enzyme IIA component of PTS |
| yhbJ | 1789598 | predicted protein with nucleoside triphosphate hydrolase domain |
| npr | 1789599 | phosphohistidinoprotein-hexose phosphotransferase component of N-regulated PTS system (Npr) |
| yrbL | 1789600 | predicted protein |
| mtgA | 1789601 | biosynthetic peptidoglycan transglycosylase |
| elbB | 87082228 | isoprenoid biosynthesis protein with amidotransferase-like domain |
| arcB | 48994928 | hybrid sensory histidine kinase in two-component regulatory system with ArcA |
| yhcC | 2367204 | predicted Fe-S oxidoreductase |
| gltB | 1789605 | glutamate synthase, large subunit |
| gltD | 1789606 | glutamate synthase, 4Fe-4S protein, small subunit |
| yhcH | 1789615 | conserved protein |
| nanK | 87082230 | predicted N-acetylmannosamine kinase |
| nanE | 1789617 | predicted N-acetylmannosamine-6-P epimerase |
| nanT | 87082231 | sialic acid transporter |
| nanA | 1789620 | N-acetylneuraminate lyase |
| nanR | 1789621 | DNA-binding transcriptional dual regulator |
| sspB | 1789623 | ClpXP protease specificity-enhancing factor |
| sspA | 1789624 | stringent starvation protein A |
| rpsI | 1789625 | 30S ribosomal subunit protein S9 |
| rplM | 1789626 | 50S ribosomal subunit protein L13 |
| yhcM | 1789627 | conserved protein with nucleoside triphosphate hydrolase domain |
| yhcB | 87082232 | conserved protein |
| degQ | 1789629 | serine endoprotease, periplasmic |
| degS | 1789630 | serine endoprotease, periplasmic |
| mdh | 1789632 | malate dehydrogenase, NAD(P)-binding |
| argR | 1789633 | DNA-binding transcriptional dual regulator, L-arginine-binding |
| yhcN | 87082233 | conserved protein |
| yhcO | 1789635 | predicted barnase inhibitor |
| aaeB | 1789636 | p-hydroxybenzoic acid efflux system component |
| aaeA | 1789637 | p-hydroxybenzoic acid efflux system component |
| aaeX | 87082234 | membrane protein of efflux system |
| aaeR | 1789639 | predicted DNA-binding transcriptional regulator, efflux system |
| tldD | 1789640 | predicted peptidase |
| yhdP | 48994929 | conserved membrane protein, predicted transporter |
| rng | 87082235 | ribonuclease G |
| yhdE | 1789646 | conserved protein |
| mreD | 1789647 | cell wall structural complex MreBCD transmembrane component MreD |
| mreC | 1789648 | cell wall structural complex MreBCD transmembrane component MreC |
| mreB | 87082236 | cell wall structural complex MreBCD, actin-like component MreB |
| yhdT | 1789655 | conserved inner membrane protein |
| prmA | 2367208 | methylase for 50S ribosomal subunit protein L11 |
| dusB | 1789660 | tRNA-dihydrouridine synthase B |
| fis | 1789661 | global DNA-binding transcriptional dual regulator |
| acrE | 1789665 | cytoplasmic membrane lipoprotein |
| acrF | 1789666 | multidrug efflux system protein |
| yhdY | 87082239 | predicted amino-acid transporter subunit |
| yhdZ | 1789672 | predicted amino-acid transporter subunit |
| yrdB | 1789674 | conserved protein |
| aroE | 1789675 | dehydroshikimate reductase, NAD(P)-binding |
| rimN | 2367210 | predicted ribosome maturation factor |
| yrdD | 48994931 | predicted DNA topoisomerase |
| smg | 1789679 | conserved protein |
| smf | 48994932 | conserved protein |
| def | 1789682 | peptide deformylase |
| fmt | 1789683 | 10-formyltetrahydrofolate:L-methionyl-tRNA(fMet) N-formyltransferase |
| rsmB | 2367212 | 16S rRNA m(5)C967 methyltransferase, S-adenosyl-L-methionine-dependent |
| trkA | 1789685 | NAD-binding component of TrK potassium transporter |
| rplQ | 1789689 | 50S ribosomal subunit protein L17 |
| rpoA | 1789690 | RNA polymerase, alpha subunit |
| rpsD | 1789691 | 30S ribosomal subunit protein S4 |
| rpsK | 1789692 | 30S ribosomal subunit protein S11 |
| rpsM | 1789693 | 30S ribosomal subunit protein S13 |
| rpmJ | 1789695 | 50S ribosomal subunit protein L36 |
| rplE | 1789704 | 50S ribosomal subunit protein L5 |
| rplX | 1789705 | 50S ribosomal subunit protein L24 |
| rplN | 1789706 | 50S ribosomal subunit protein L14 |
| yheV | 87082247 | predicted protein |
| kefG | 1789750 | component of potassium effux complex with KefB |
| yheS | 1789751 | fused predicted transporter subunits of ABC superfamily: ATP-binding components |
| yheT | 1789752 | predicted hydrolase |
| yheU | 1789753 | conserved protein |
| prkB | 2367214 | predicted phosphoribulokinase |
| crp | 1789756 | DNA-binding transcriptional dual regulator |
| yhfK | 87082248 | conserved inner membrane protein |
| argD | 1789759 | bifunctional acetylornithine aminotransferase/ succinyldiaminopimelate aminotransferase |
| pabA | 1789760 | aminodeoxychorismate synthase, subunit II |
| fic | 1789761 | stationary-phase protein, cell division |
| yhfG | 1789762 | predicted protein |
| ppiA | 1789763 | peptidyl-prolyl cis-trans isomerase A (rotamase A) |
| tsgA | 1789764 | predicted transporter |
| nirB | 1789765 | nitrite reductase, large subunit, NAD(P)H-binding |
| nirD | 1789766 | nitrite reductase, NAD(P)H-binding, small subunit |
| nirC | 87082249 | nitrite transporter |
| cysG | 1789768 | fused siroheme synthase 1,3-dimethyluroporphyriongen III dehydrogenase and siroheme ferrochelatase/uroporphyrinogen methyltransferase |
| trpS | 1789786 | tryptophanyl-tRNA synthetase |
| gph | 1789787 | phosphoglycolate phosphatase |
| rpe | 1789788 | D-ribulose-5-phosphate 3-epimerase |
| dam | 1789789 | DNA adenine methylase |
| damX | 1789790 | predicted protein |
| aroB | 1789791 | 3-dehydroquinate synthase |
| aroK | 87082255 | shikimate kinase I |
| hofQ | 1789793 | predicted fimbrial transporter |
| hofO | 1789795 | conserved membrane protein |
| hofN | 1789796 | predicted fimbrial assembly protein |
| hofM | 87082257 | predicted pilus assembly protein |
| mrcA | 87082258 | fused penicillin-binding protein 1a: murein transglycosylase/murein transpeptidase |
| nudE | 1789800 | ADP-ribose diphosphatase |
| yrfF | 1789801 | predicted inner membrane protein |
| yrfG | 87082259 | predicted hydrolase |
| hslR | 1789803 | ribosome-associated heat shock protein Hsp15 |
| hslO | 87082260 | heat shock protein Hsp33 |
| yhgE | 1789805 | predicted inner membrane protein |
| pck | 1789807 | phosphoenolpyruvate carboxykinase |
| envZ | 1789808 | sensory histidine kinase in two-component regulatory system with OmpR |
| ompR | 1789809 | DNA-binding response regulator in two-component regulatory system with EnvZ |
| greB | 87082261 | transcription elongation factor |
| yhgF | 87082262 | predicted transcriptional accessory protein |
| feoA | 1789812 | ferrous iron transporter, protein A |
| feoB | 1789813 | fused ferrous iron transporter, protein B: GTP-binding protein/membrane protein |
| feoC | 1789814 | predicted DNA-binding transcriptional regulator |
| bioH | 1789817 | carboxylesterase of pimeloyl-CoA synthesis |
| gntX | 87082263 | gluconate periplasmic binding protein with phosphoribosyltransferase domain, GNT I system |
| nfuA | 1789819 | predicted gluconate transport associated protein |
| gntT | 48994935 | gluconate transporter, high-affinity GNT I system |
| malQ | 1789821 | 4-alpha-glucanotransferase (amylomaltase) |
| malP | 48994936 | maltodextrin phosphorylase |
| malT | 2367223 | fused conserved protein: DNA-binding transcriptional activator/maltotriose-ATP-binding protein |
| rtcA | 48994937 | RNA 3'-terminal phosphate cyclase |
| glpR | 1789829 | DNA-binding transcriptional repressor |
| glpG | 48994938 | predicted intramembrane serine protease |
| glpE | 1789831 | thiosulfate:cyanide sulfurtransferase (rhodanese) |
| glpD | 2367226 | sn-glycerol-3-phosphate dehydrogenase, aerobic, FAD/NAD(P)-binding |
| glgP | 2367228 | glycogen phosphorylase |
| glgA | 1789836 | glycogen synthase |
| glgC | 1789837 | glucose-1-phosphate adenylyltransferase |
| glgX | 2367229 | glycogen debranching enzyme |
| glgB | 1789839 | 1,4-alpha-glucan branching enzyme |
| asd | 1789841 | aspartate-semialdehyde dehydrogenase, NAD(P)-binding |
| yhgN | 1789842 | predicted antibiotic transporter |
| gntU | 48994939 | gluconate transporter, low affinity GNT 1 system |
| gntK | 87082265 | gluconate kinase 2 |
| gntR | 48994940 | DNA-binding transcriptional repressor |
| yhhW | 1789847 | predicted protein |
| yhhX | 1789848 | predicted oxidoreductase with NAD(P)-binding Rossmann-fold domain |
| ggt | 1789856 | gamma-glutamyltranspeptidase |
| yhhA | 1789857 | conserved protein |
| ugpQ | 1789858 | glycerophosphodiester phosphodiesterase, cytosolic |
| ugpC | 87082267 | glycerol-3-phosphate transporter subunit |
| ugpE | 1789860 | glycerol-3-phosphate transporter subunit |
| livF | 87082268 | leucine/isoleucine/valine transporter subunit |
| livG | 1789864 | leucine/isoleucine/valine transporter subunit |
| livM | 1789865 | leucine/isoleucine/valine transporter subunit |
| livH | 1789866 | leucine/isoleucine/valine transporter subunit |
| livK | 1789867 | leucine transporter subunit |
| yhhK | 1789869 | conserved protein |
| livJ | 48994941 | leucine/isoleucine/valine transporter subunit |
| rpoH | 1789871 | RNA polymerase, sigma 32 (sigma H) factor |
| ftsX | 1789872 | predicted transporter subunit: membrane component of ABC superfamily |
| ftsE | 1789873 | predicted transporter subunit: ATP-binding component of ABC superfamily |
| ftsY | 1789874 | fused Signal Recognition Particle (SRP) receptor: membrane binding protein/conserved protein |
| rsmD | 1789875 | 16S rRNA m(2)G966 methyltransferase, SAM-dependent |
| yhhL | 1789876 | conserved inner membrane protein |
| yhhM | 1789877 | conserved protein |
| yhhN | 1789878 | conserved inner membrane protein |
| zntA | 1789879 | zinc, cobalt and lead efflux system |
| sirA | 1789881 | conserved protein required for cell growth |
| yhhQ | 1789882 | conserved inner membrane protein |
| dcrB | 87082269 | periplasmic protein |
| yhhS | 87082270 | predicted transporter |
| acpT | 1789886 | holo-(acyl carrier protein) synthase 2 |
| nikA | 1789887 | nickel transporter subunit |
| nikB | 1789888 | nickel transporter subunit |
| nikC | 1789889 | nickel transporter subunit |
| nikD | 1789890 | nickel transporter subunit |
| nikE | 1789891 | nickel transporter subunit |
| nikR | 1789892 | DNA-binding transcriptional repressor, Ni-binding |
| yhiN | 2367234 | predicted oxidoreductase with FAD/NAD(P)-binding domain |
| pitA | 1789907 | phosphate transporter, low-affinity |
| uspB | 1789908 | predicted universal stress (ethanol tolerance) protein B |
| uspA | 1789909 | universal stress global response regulator |
| dtpB | 1789911 | predicted transporter |
| yhiQ | 1789912 | predicted SAM-dependent methyltransferase |
| prlC | 1789913 | oligopeptidase A |
| yhiR | 1789914 | predicted DNA (exogenous) processing protein |
| gor | 1789915 | glutathione oxidoreductase |
| dinQ | 145693192 | Damage inducible, function unknown |
| arsC | 1789918 | arsenate reductase |
| slp | 87082278 | outer membrane lipoprotein |
| dctR | 1789923 | predicted DNA-binding ranscriptional regulator |
| hdeB | 87082279 | acid-resistance protein |
| hdeA | 1789926 | stress response protein acid-resistance protein |
| hdeD | 1789927 | acid-resistance membrane protein |
| gadE | 1789928 | DNA-binding transcriptional activator |
| mdtF | 1789930 | multidrug transporter, RpoS-dependent |
| gadW | 1789932 | DNA-binding transcriptional activator |
| gadX | 1789933 | DNA-binding transcriptional dual regulator |
| gadA | 1789934 | glutamate decarboxylase A, PLP-dependent |
| yhjC | 145693193 | predicted DNA-binding transcriptional regulator |
| yhjD | 1789939 | conserved inner membrane protein |
| yhjE | 1789941 | predicted transporter |
| yhjG | 2367236 | predicted outer membrane biogenesis protein |
| yhjH | 87082280 | EAL domain containing protein involved in flagellar function |
| kdgK | 87082281 | ketodeoxygluconokinase |
| yhjJ | 1789946 | predicted zinc-dependent peptidase |
| dctA | 1789947 | C4-dicarboxylic acid, orotate and citrate transporter |
| yhjK | 145693194 | predicted diguanylate cyclase |
| ldrD | 48994945 | toxic polypeptide, small |
| yhjV | 1789961 | predicted transporter |
| dppF | 1789962 | dipeptide transporter |
| dppD | 1789963 | dipeptide transporter |
| dppC | 1789964 | dipeptide transporter |
| dppB | 1789965 | dipeptide transporter |
| dppA | 1789966 | dipeptide transporter |
| yhjX | 1789969 | predicted transporter |
| yhjY | 87082287 | conserved protein |
| tag | 1789971 | 3-methyl-adenine DNA glycosylase I, constitutive |
| yiaC | 1789972 | predicted acyltransferase with acyl-CoA N-acyltransferase domain |
| bisC | 145693196 | biotin sulfoxide reductase |
| ghrB | 87082289 | 2-keto-D-gluconate reductase (glyoxalate reductase) (2-ketoaldonate reductase) |
| yiaF | 87082290 | conserved protein |
| yiaG | 1789978 | predicted transcriptional regulator |
| cspA | 1789979 | major cold shock protein |
| hokA | 48994947 | toxic polypeptide, small |
| glyS | 1789982 | glycine tRNA synthetase, beta subunit |
| glyQ | 1789983 | glycine tRNA synthetase, alpha subunit |
| ysaB | 87082291 | predicted protein |
| ysaA | 2367245 | predicted hydrogenase, 4Fe-4S ferredoxin-type component |
| yiaS | 1790008 | L-ribulose-5-phosphate 4-epimerase |
| selB | 2367247 | selenocysteinyl-tRNA-specific translation factor |
| selA | 2367248 | selenocysteine synthase |
| yibF | 1790018 | predicted glutathione S-transferase |
| lldP | 1790031 | L-lactate permease |
| lldR | 1790032 | DNA-binding transcriptional repressor |
| lldD | 1790033 | L-lactate dehydrogenase, FMN-linked |
| yibK | 1790034 | predicted rRNA methylase |
| cysE | 1790035 | serine acetyltransferase |
| gpsA | 1790037 | glycerol-3-phosphate dehydrogenase (NAD+) |
| secB | 1790038 | protein export chaperone |
| grxC | 1790039 | glutaredoxin 3 |
| yibN | 1790040 | predicted rhodanese-related sulfurtransferase |
| gpmM | 1790041 | phosphoglycero mutase III, cofactor-independent |
| envC | 87082297 | protease with a role in cell division |
| yibQ | 87082298 | predicted polysaccharide deacetylase |
| tdh | 1790045 | threonine 3-dehydrogenase, NAD(P)-binding |
| kbl | 1790046 | glycine C-acetyltransferase |
| rfaD | 1790049 | ADP-L-glycero-D-mannoheptose-6-epimerase, NAD(P)-binding |
| rfaF | 1790050 | ADP-heptose:LPS heptosyltransferase II |
| rfaP | 1790060 | kinase that phosphorylates core heptose of lipopolysaccharide |
| rfaG | 1790061 | glucosyltransferase I |
| waaA | 1790064 | 3-deoxy-D-manno-octulosonic-acid transferase (KDO transferase) |
| coaD | 1790065 | pantetheine-phosphate adenylyltransferase |
| mutM | 1790066 | formamidopyrimidine/5-formyluracil/ 5-hydroxymethyluracil DNA glycosylase |
| rpmG | 1790067 | 50S ribosomal subunit protein L33 |
| dfp | 87082301 | fused 4'-phosphopantothenoylcysteine decarboxylase/phosphopantothenoylcysteine synthetase, FMN-binding |
| dut | 1790071 | deoxyuridinetriphosphatase |
| slmA | 87082302 | division inhibitor |
| pyrE | 1790073 | orotate phosphoribosyltransferase |
| rph | 157672248 | defective ribonuclease PH |
| yicC | 1790075 | conserved protein |
| yicG | 87082304 | conserved inner membrane protein |
| gmk | 1790080 | guanylate kinase |
| rpoZ | 1790081 | RNA polymerase, omega subunit |
| spoT | 1790082 | bifunctional (p)ppGpp synthetase II/ guanosine-3',5'-bis pyrophosphate 3'-pyrophosphohydrolase |
| trmH | 1790083 | tRNA mG18-2'-O-methyltransferase, SAM-dependen |
| recG | 2367254 | ATP-dependent DNA helicase |
| gltS | 1790085 | glutamate transporter |
| xanP | 1790087 | predicted transporter |
| yicH | 1790088 | conserved protein |
| yicJ | 87082306 | predicted transporter |
| nlpA | 1790093 | cytoplasmic membrane lipoprotein-28 |
| nepI | 145693198 | predicted transporter |
| yicN | 87082308 | conserved protein |
| yicO | 87082309 | predicted xanthine/uracil permase |
| ade | 1790098 | cryptic adenine deaminase |
| uhpT | 2367259 | hexose phosphate transporter |
| uhpC | 87082310 | membrane protein regulates uhpT expression |
| uhpB | 87082311 | sensory histidine kinase in two-component regulatory sytem with UhpA |
| uhpA | 1790102 | DNA-binding response regulator in two-component regulatory system wtih UhpB |
| ilvN | 1790103 | acetolactate synthase I, small subunit |
| ilvB | 1790104 | acetolactate synthase I, large subunit |
| ivbL | 1790106 | ilvB operon leader peptide |
| emrD | 87082312 | multidrug efflux system protein |
| yidF | 1790108 | predicted DNA-binding transcriptional regulator |
| yidG | 1790109 | predicted inner membrane protein |
| yidH | 1790110 | conserved inner membrane protein |
| yidE | 87082315 | predicted transporter |
| ibpB | 87082316 | heat shock chaperone |
| ibpA | 1790122 | heat shock chaperone |
| yidQ | 87082317 | conserved outer membrane protein |
| yidR | 87082318 | conserved protein |
| yidB | 87082321 | conserved protein |
| gyrB | 48994957 | DNA gyrase, subunit B |
| recF | 1790135 | gap repair protein |
| dnaN | 1790136 | DNA polymerase III, beta subunit |
| dnaA | 2367267 | chromosomal replication initiator protein DnaA, DNA-binding transcriptional dual regulator |
| rpmH | 1790138 | 50S ribosomal subunit protein L34 |
| rnpA | 1790139 | protein C5 component of RNase P |
| yidD | 87082322 | predicted protein |
| yidC | 1790140 | cytoplasmic insertase into membrane protein, Sec system |
| mnmE | 2367268 | GTPase |
| mdtL | 1790146 | multidrug efflux system protein |
| yieE | 1790148 | predicted phosphopantetheinyl transferase |
| yieF | 1790149 | chromate reductase, Class I, flavoprotein |
| purP | 1790150 | predicted inner membrane protein |
| phoU | 1790161 | negative regulator of PhoR/PhoB two-component regulator |
| pstB | 1790162 | phosphate transporter subunit |
| pstA | 1790163 | phosphate transporter subunit |
| pstC | 1790164 | phosphate transporter subunit |
| pstS | 2367271 | phosphate transporter subunit |
| glmS | 1790167 | L-glutamine:D-fructose-6-phosphate aminotransferase |
| glmU | 1790168 | fused N-acetyl glucosamine-1-phosphate uridyltransferase/glucosamine-1-phosphate acetyl transferase |
| atpC | 1790169 | F1 sector of membrane-bound ATP synthase, epsilon subunit |
| atpD | 1790170 | F1 sector of membrane-bound ATP synthase, beta subunit |
| atpG | 1790171 | F1 sector of membrane-bound ATP synthase, gamma subunit |
| atpA | 1790172 | F1 sector of membrane-bound ATP synthase, alpha subunit |
| atpH | 1790173 | F1 sector of membrane-bound ATP synthase, delta subunit |
| atpF | 1790174 | F0 sector of membrane-bound ATP synthase, subunit b |
| atpE | 1790175 | F0 sector of membrane-bound ATP synthase, subunit c |
| atpB | 1790176 | F0 sector of membrane-bound ATP synthase, subunit a |
| rsmG | 1790179 | methyltransferase, SAM-dependent methyltransferase, glucose-inhibited cell-division protein |
| mioC | 1790181 | FMN-binding protein MioC |
| asnC | 1790182 | DNA-binding transcriptional dual regulator |
| asnA | 1790183 | asparagine synthetase A |
| viaA | 48994959 | predicted von Willibrand factor containing protein |
| ravA | 87082326 | fused predicted transcriptional regulator: sigma54 activator protein/conserved protein |
| kup | 48994960 | potassium transporter |
| rbsD | 87082327 | predicted cytoplasmic sugar-binding protein |
| rbsC | 1790191 | D-ribose transporter subunit |
| rbsB | 1790192 | D-ribose transporter subunit |
| rbsK | 1790193 | ribokinase |
| hsrA | 1790195 | predicted multidrug or homocysteine efflux system |
| yieP | 48994961 | predicted transcriptional regulator |
| hdfR | 48994962 | DNA-binding transcriptional regulator |
| yifE | 2367277 | conserved protein |
| ilvL | 1790202 | ilvG operon leader peptide |
| ilvM | 1790204 | acetolactate synthase II, small subunit |
| ilvE | 48994963 | branched-chain amino-acid aminotransferase |
| ilvD | 48994964 | dihydroxyacid dehydratase |
| ilvA | 1790207 | threonine deaminase |
| ilvY | 1790208 | DNA-binding transcriptional dual regulator |
| ilvC | 1790210 | ketol-acid reductoisomerase, NAD(P)-binding |
| ppiC | 1790211 | peptidyl-prolyl cis-trans isomerase C (rotamase C) |
| rep | 48994965 | DNA helicase and single-stranded DNA-dependent ATPase |
| gpp | 48994966 | guanosine pentaphosphatase/exopolyphosphatase |
| rhlB | 1790214 | ATP-dependent RNA helicase |
| trxA | 87082331 | thioredoxin 1 |
| rho | 1790217 | transcription termination factor |
| rfe | 1790218 | UDP-GlcNAc:undecaprenylphosphate GlcNAc-1-phosphate transferase |
| wzzE | 87082332 | Entobacterial Common Antigen (ECA) polysaccharide chain length modulation protein |
| rffE | 48994967 | UDP-N-acetyl glucosamine-2-epimerase |
| rffD | 48994968 | UDP-N-acetyl-D-mannosaminuronic acid dehydrogenase |
| rffG | 48994969 | dTDP-glucose 4,6-dehydratase |
| rffH | 1790224 | glucose-1-phosphate thymidylyltransferase |
| rffC | 145693203 | TDP-fucosamine acetyltransferase |
| rffA | 2367285 | TDP-4-oxo-6-deoxy-D-glucose transaminase |
| wzxE | 1790227 | O-antigen translocase |
| rffT | 48994971 | TDP-Fuc4NAc:lipidIIFuc4NAc transferase |
| wzyE | 2367288 | predicted Wzy protein involved in ECA polysaccharide chain elongation |
| rffM | 2367289 | UDP-N-acetyl-D-mannosaminuronic acid transferase |
| yifK | 48994972 | predicted transporter |
| hemY | 1790234 | predicted protoheme IX synthesis protein |
| hemX | 1790235 | predicted uroporphyrinogen III methylase |
| hemD | 1790236 | uroporphyrinogen III synthase |
| hemC | 48994974 | hydroxymethylbilane synthase |
| cyaA | 1790238 | adenylate cyclase |
| cyaY | 1790239 | frataxin, iron-binding and oxidizing protein |
| yifL | 87082333 | predicted lipoprotein |
| dapF | 87082334 | diaminopimelate epimerase |
| yigA | 1790243 | conserved protein |
| xerC | 1790244 | site-specific tyrosine recombinase |
| yigB | 2367295 | predicted hydrolase |
| uvrD | 2367296 | DNA-dependent ATPase I and helicase II |
| corA | 2367297 | magnesium/nickel/cobalt transporter |
| rarD | 48994976 | predicted chloramphenical resistance permease |
| yigI | 87082336 | conserved protein |
| pldA | 2367300 | outer membrane phospholipase A |
| recQ | 48994977 | ATP-dependent DNA helicase |
| rhtC | 48994978 | threonine efflux system |
| rhtB | 48994979 | neutral amino-acid efflux system |
| pldB | 48994980 | lysophospholipase L(2) |
| yigL | 48994981 | predicted hydrolase |
| yigM | 1790261 | predicted inner membrane protein |
| metR | 1790262 | DNA-binding transcriptional activator, homocysteine-binding |
| metE | 2367304 | 5-methyltetrahydropteroyltriglutamate- homocysteine S-methyltransferase |
| ysgA | 48994982 | predicted hydrolase |
| udp | 1790265 | uridine phosphorylase |
| rmuC | 1790266 | predicted recombination limiting protein |
| ubiE | 48994983 | bifunctional 2-octaprenyl-6-methoxy-1,4-benzoquinone methylase/ S-adenosylmethionine:2-DMK methyltransferase |
| yigP | 2367308 | conserved protein |
| ubiB | 2367309 | 2-octaprenylphenol hydroxylase |
| tatA | 87082337 | TatABCE protein translocation system subunit |
| tatB | 48994984 | TatABCE protein translocation system subunit |
| tatC | 2367313 | TatABCE protein translocation system subunit |
| tatD | 48994985 | DNase, magnesium-dependent |
| rfaH | 1790276 | DNA-binding transcriptional antiterminator |
| ubiD | 1790277 | 3-octaprenyl-4-hydroxybenzoate decarboxylase |
| fre | 2367314 | flavin reductase |
| fadA | 48994986 | 3-ketoacyl-CoA thiolase (thiolase I) |
| fadB | 1790281 | fused 3-hydroxybutyryl-CoA epimerase/delta(3)-cis-delta(2)-trans-enoyl-CoA isomerase/enoyl-CoA hydratase/3-hydroxyacyl-CoA dehydrogenase |
| pepQ | 1790282 | proline dipeptidase |
| yigZ | 87082338 | predicted elongation factor |
| trkH | 48994987 | potassium transporter |
| hemG | 1790285 | protoporphyrin oxidase, flavoprotein |
| mobB | 145693204 | molybdopterin-guanine dinucleotide biosynthesis protein B |
| mobA | 1790288 | molybdopterin-guanine dinucleotide synthase |
| yihD | 1790289 | conserved protein |
| rdoA | 1790290 | Thr/Ser kinase implicated in Cpx stress response |
| dsbA | 1790291 | periplasmic protein disulfide isomerase I |
| yihG | 1790293 | predicted endonuclease |
| polA | 1790294 | fused DNA polymerase I 5'->3' polymerase/3'->5' exonuclease/5'->3' exonuclease |
| yihA | 145693205 | GTP-binding protein |
| yihI | 1790297 | conserved protein |
| hemN | 87082341 | coproporphyrinogen III oxidase, SAM and NAD(P)H dependent, oxygen-independent |
| glnG | 1790299 | fused DNA-binding response regulator in two-component regulatory system with GlnL: response regulator/sigma54 interaction protein |
| glnL | 1790300 | sensory histidine kinase in two-component regulatory system with GlnG |
| glnA | 1790301 | glutamine synthetase |
| typA | 48994988 | GTP-binding protein |
| yihX | 87082345 | predicted hydrolase |
| yihY | 1790319 | predicted inner membrane protein |
| dtd | 1790320 | D-tyr-tRNA(Tyr) deacylase |
| yiiD | 1790321 | predicted acetyltransferase |
| fdhE | 1790324 | formate dehydrogenase formation protein |
| fdoI | 1790325 | formate dehydrogenase-O, cytochrome b556 subunit |
| fdoH | 1790326 | formate dehydrogenase-O, Fe-S subunit |
| fdoG | 3868720 | formate dehydrogenase-O, large subunit |
| fdhD | 1790329 | formate dehydrogenase formation protein |
| rhaM | 1790335 | L-rhamnose mutarotase |
| rhaD | 1790336 | rhamnulose-1-phosphate aldolase |
| rhaA | 48994990 | L-rhamnose isomerase |
| rhaS | 1790339 | DNA-binding transcriptional activator, L-rhamnose-binding |
| rhaT | 1790341 | L-rhamnose:proton symporter |
| sodA | 1790342 | superoxide dismutase, Mn |
| yiiM | 87082350 | conserved protein |
| cpxA | 1790346 | sensory histidine kinase in two-component regulatory system with CpxR |
| cpxR | 2367329 | DNA-binding response regulator in two-component regulatory system with CpxA |
| cpxP | 48994991 | periplasmic protein combats stress |
| fieF | 1790349 | zinc transporter |
| pfkA | 1790350 | 6-phosphofructokinase I |
| sbp | 1790351 | sulfate transporter subunit |
| tpiA | 1790353 | triosephosphate isomerase |
| yiiQ | 1790354 | conserved protein |
| yiiR | 1790355 | conserved inner membrane protein |
| yiiS | 1790357 | conserved protein |
| uspD | 1790358 | stress-induced protein |
| fpr | 1790359 | ferredoxin-NADP reductase |
| glpX | 1790360 | fructose 1,6-bisphosphatase II |
| glpK | 1790361 | glycerol kinase |
| glpF | 1790362 | glycerol facilitator |
| zapB | 1790363 | conserved protein |
| rraA | 1790364 | ribonuclease E (RNase E) inhibitor protein |
| menA | 1790365 | 1,4-dihydroxy-2-naphthoate octaprenyltransferase |
| hslU | 1790366 | molecular chaperone and ATPase component of HslUV protease |
| hslV | 1790367 | peptidase component of the HslUV protease |
| cytR | 1790369 | DNA-binding transcriptional dual regulator |
| priA | 1790370 | Primosome factor n' (replication factor Y) |
| rpmE | 1790371 | 50S ribosomal subunit protein L31 |
| yiiX | 1790372 | predicted peptidoglycan peptidase |
| metJ | 1790373 | DNA-binding transcriptional repressor, S-adenosylmethionine-binding |
| metB | 1790375 | cystathionine gamma-synthase, PLP-dependent |
| metL | 1790376 | fused aspartokinase II/homoserine dehydrogenase II |
| metF | 1790377 | 5,10-methylenetetrahydrofolate reductase |
| katG | 1790378 | catalase/hydroperoxidase HPI(I) |
| yijE | 87082351 | predicted permease |
| yijF | 1790380 | conserved protein |
| gldA | 87082352 | glycerol dehydrogenase, NAD |
| fsaB | 1790382 | fructose-6-phosphate aldolase 2 |
| ptsA | 48994992 | fused predicted PTS enzymes: Hpr component/enzyme I component/enzyme IIA component |
| frwC | 1790386 | predicted enzyme IIC component of PTS |
| frwB | 1790387 | predicted enzyme IIB component of PTS |
| pflD | 1790388 | predicted formate acetyltransferase 2 (pyruvate formate lyase II) |
| pflC | 1790389 | pyruvate formate lyase II activase |
| frwD | 1790390 | predicted enzyme IIB component of PTS |
| yijO | 1790391 | predicted DNA-binding transcriptional regulator |
| yijP | 1790392 | conserved inner membrane protein |
| ppc | 1790393 | phosphoenolpyruvate carboxylase |
| argE | 1790395 | acetylornithine deacetylase |
| argB | 145693208 | acetylglutamate kinase |
| argH | 1790398 | argininosuccinate lyase |
| oxyR | 1790399 | DNA-binding transcriptional dual regulator |
| sthA | 87082354 | pyridine nucleotide transhydrogenase, soluble |
| fabR | 145693209 | DNA-binding transcriptional repressor |
| yijD | 1790402 | conserved inner membrane protein |
| trmA | 1790403 | tRNA m(5)U54 methyltransferase, SAM-dependen |
| murI | 87082355 | glutamate racemase |
| murB | 1790407 | UDP-N-acetylenolpyruvoylglucosamine reductase, FAD-binding |
| birA | 1790408 | bifunctional biotin-[acetylCoA carboxylase] holoenzyme synthetase/ DNA-binding transcriptional repressor, bio-5'-AMP-binding |
| rplL | 1790418 | 50S ribosomal subunit protein L7/L12 |
| rpoB | 1790419 | RNA polymerase, beta subunit |
| rpoC | 2367335 | RNA polymerase, beta prime subunit |
| thiH | 1790423 | thiamin biosynthesis ThiGH complex subunit |
| thiG | 48994993 | thiamin biosynthesis ThiGH complex subunit |
| thiS | 48994994 | sulphur carrier protein |
| thiF | 87082356 | thiamin (thiazole moiety) biosynthesis protein |
| thiE | 1790426 | thiamin phosphate synthase (thiamin phosphate pyrophosphorylase) |
| thiC | 1790427 | thiamin (pyrimidine moiety) biosynthesis protein |
| rsd | 1790428 | stationary phase protein, binds sigma 70 RNA polymerase subunit |
| nudC | 48994995 | NADH pyrophosphatase |
| hemE | 2367337 | uroporphyrinogen decarboxylase |
| nfi | 87082357 | endonuclease V |
| yjaG | 1790432 | conserved protein |
| hupA | 1790433 | HU, DNA-binding transcriptional regulator, alpha subunit |
| yjaH | 1790434 | conserved protein |
| zraP | 87082358 | Zn-binding periplasmic protein |
| zraS | 1790436 | sensory histidine kinase in two-component regulatory system with ZraR |
| zraR | 1790437 | fused DNA-binding response regulator in two-component regulatory system with ZraS: response regulator/sigma54 interaction protein |
| purD | 1790438 | phosphoribosylglycinamide synthetase phosphoribosylamine-glycine ligase |
| purH | 1790439 | fused IMP cyclohydrolase/phosphoribosylaminoimidazolecarboxamide formyltransferase |
| metA | 1790443 | homoserine O-transsuccinylase |
| aceB | 1790444 | malate synthase A |
| aceA | 1790445 | isocitrate lyase |
| iclR | 87082359 | DNA-binding transcriptional repressor |
| metH | 1790450 | homocysteine-N5-methyltetrahydrofolate transmethylase, B12-dependent |
| yjbB | 1790451 | predicted transporter |
| pepE | 1790452 | (alpha)-aspartyl dipeptidase |
| rluF | 1790453 | 23S rRNA U2604 pseudouridine synthase |
| lysC | 1790455 | aspartokinase III |
| pgi | 1790457 | glucosephosphate isomerase |
| yjbE | 1790458 | predicted protein |
| yjbF | 87082360 | predicted lipoprotein |
| yjbG | 1790460 | conserved protein |
| yjbH | 1790461 | predicted porin |
| yjbT | 145693210 | hypothetical protein, no homologs |
| psiE | 1790462 | predicted phosphate starvation inducible protein |
| ubiC | 87082361 | chorismate pyruvate lyase |
| ubiA | 1790473 | p-hydroxybenzoate octaprenyltransferase |
| plsB | 87082362 | glycerol-3-phosphate O-acyltransferase |
| dgkA | 1790475 | diacylglycerol kinase |
| lexA | 1790476 | DNA-binding transcriptional repressor of SOS regulon |
| dinF | 1790477 | DNA-damage-inducible SOS response protein |
| yjbJ | 1790479 | predicted stress response protein |
| zur | 87082363 | DNA-binding transcriptional repressor, Zn(II)-binding |
| dusA | 145693211 | tRNA-dihydrouridine synthase A |
| qorA | 1790485 | quinone oxidoreductase, NADPH-dependent |
| dnaB | 1790486 | replicative DNA helicase |
| alr | 1790487 | alanine racemase 1, PLP-binding, biosynthetic |
| tyrB | 1790488 | tyrosine aminotransferase, tyrosine-repressible, PLP-dependent |
| yjbQ | 1790491 | conserved protein |
| yjbR | 2367342 | conserved protein |
| uvrA | 2367343 | ATPase and DNA damage recognition protein of nucleotide excision repair excinuclease UvrABC |
| ssb | 1790494 | single-stranded DNA-binding protein |
| yjcE | 1790501 | predicted cation/proton antiporter |
| phnD | 1790543 | phosphonate/organophosphate ester transporter subunit |
| phnC | 1790544 | phosphonate/organophosphate ester transporter subunit |
| yjdN | 1790546 | conserved protein |
| yjdM | 1790547 | conserved protein |
| yjdA | 1790548 | conserved protein with nucleoside triphosphate hydrolase domain |
| yjcZ | 87082371 | conserved protein |
| proP | 1790550 | proline/glycine betaine transporter |
| basS | 1790551 | sensory histidine kinase in two-component regulatory system with BasR |
| basR | 1790552 | DNA-binding response regulator in two-component regulatory system with BasS |
| eptA | 87082372 | predicted metal dependent hydrolase |
| adiC | 2367353 | arginine:agmatin |
| adiY | 1790557 | DNA-binding transcriptional activator |
| adiA | 221142684 | biodegradative arginine decarboxylase |
| melR | 1790559 | DNA-binding transcriptional dual regulator |
| melA | 1790560 | alpha-galactosidase, NAD(P)-binding |
| yjdF | 1790562 | conserved inner membrane protein |
| fumB | 1790564 | anaerobic class I fumarate hydratase (fumarase B) |
| dcuB | 1790565 | C4-dicarboxylate antiporter |
| dcuR | 1790566 | DNA-binding response regulator in two-component regulatory system with DcuS |
| dcuS | 1790567 | sensory histidine kinase in two-component regulatory system with DcuR, regulator of anaerobic fumarate respiration |
| yjdO | 87082373 | predicted protein |
| lysU | 1790571 | lysine tRNA synthetase, inducible |
| cadA | 1790573 | lysine decarboxylase 1 |
| yjdC | 87082374 | predicted transcriptional regulator |
| dsbD | 1790578 | fused thiol:disulfide interchange protein: activator of DsbC/conserved protein |
| cutA | 1790579 | copper binding protein, copper sensitivity |
| dcuA | 1790580 | C4-dicarboxylate antiporter |
| aspA | 87082375 | aspartate ammonia-lyase |
| fxsA | 87082376 | inner membrane protein |
| yjeH | 1790584 | predicted transporter |
| groS | 1790585 | Cpn10 chaperonin GroES, small subunit of GroESL |
| groL | 1790586 | Cpn60 chaperonin GroEL, large subunit of GroESL |
| yjeI | 87082377 | conserved protein |
| yjeK | 1790589 | predicted lysine aminomutase |
| efp | 1790590 | Elongation factor EF-P |
| ecnA | 48994998 | entericidin A membrane lipoprotein, antidote entericidin B |
| ecnB | 48994999 | entericidin B membrane lipoprotein |
| sugE | 87082378 | multidrug efflux system protein |
| blc | 1790592 | outer membrane lipoprotein (lipocalin) |
| ampC | 1790593 | beta-lactamase/D-alanine carboxypeptidase |
| frdD | 1790594 | fumarate reductase (anaerobic), membrane anchor subunit |
| frdC | 1790595 | fumarate reductase (anaerobic), membrane anchor subunit |
| frdB | 1790596 | fumarate reductase (anaerobic), Fe-S subunit |
| frdA | 1790597 | fumarate reductase (anaerobic) catalytic and NAD/flavoprotein subunit |
| poxA | 87082379 | predicted lysyl-tRNA synthetase |
| yjeP | 2367355 | predicted mechanosensitive channel |
| psd | 1790604 | phosphatidylserine decarboxylase |
| rsgA | 87082381 | ribosome small subunit-dependent GTPase A |
| yjeE | 1790610 | ATPase with strong ADP affinity |
| amiB | 1790611 | N-acetylmuramoyl-l-alanine amidase II |
| mutL | 1790612 | methyl-directed mismatch repair protein |
| miaA | 1790613 | delta(2)-isopentenylpyrophosphate tRNA-adenosine transferase |
| hfq | 1790614 | HF-I, host factor for RNA phage Q beta replication |
| hflX | 1790615 | predicted GTPase |
| hflK | 1790616 | modulator for HflB protease specific for phage lambda cII repressor |
| hflC | 1790617 | modulator for HflB protease specific for phage lambda cII repressor |
| yjeT | 1790619 | conserved inner membrane protein |
| purA | 1790620 | adenylosuccinate synthetase |
| nsrR | 1790621 | predicted DNA-binding transcriptional regulator |
| rnr | 87082383 | exoribonuclease R, RNase R |
| rlmB | 1790623 | 23S rRNA (Gm2251)-methyltransferase |
| yjfM | 1790628 | conserved protein |
| yjfC | 1790629 | predicted synthetase/amidase |
| yjfN | 87082385 | predicted protein |
| yjfO | 87082386 | conserved protein |
| yjfP | 1790634 | predicted hydrolase |
| ulaR | 1790635 | DNA-binding transcriptional dual regulator |
| ulaG | 87082387 | predicted L-ascorbate 6-phosphate lactonase |
| ulaA | 87082388 | L-ascorbate-specific enzyme IIC component of PTS |
| ulaB | 1790638 | L-ascorbate-specific enzyme IIB component of PTS |
| ulaC | 2367359 | L-ascorbate-specific enzyme IIA component of PTS |
| ulaD | 1790640 | 3-keto-L-gulonate 6-phosphate decarboxylase |
| ulaE | 1790641 | L-xylulose 5-phosphate 3-epimerase |
| ulaF | 1790642 | L-ribulose 5-phosphate 4-epimerase |
| yjfY | 1790643 | predicted protein |
| rpsF | 221142685 | 30S ribosomal subunit protein S6 |
| priB | 1790645 | primosomal protein N |
| rpsR | 1790646 | 30S ribosomal subunit protein S18 |
| rplI | 1790647 | 50S ribosomal subunit protein L9 |
| cycA | 1790653 | D-alanine/D-serine/glycine transporter |
| ytfE | 1790654 | predicted regulator of cell morphogenesis and cell wall metabolism |
| ytfF | 1790655 | predicted inner membrane protein |
| qorB | 1790656 | NAD(P)H:quinone oxidoreductase |
| ytfH | 87082391 | predicted transcriptional regulator |
| cpdB | 1790658 | 2':3'-cyclic-nucleotide 2'-phosphodiesterase |
| cysQ | 1790659 | PAPS (adenosine 3'-phosphate 5'-phosphosulfate) 3'(2'),5'-bisphosphate nucleotidase |
| ytfJ | 1790662 | predicted transcriptional regulator |
| ytfK | 87082392 | conserved protein |
| ytfL | 1790664 | predicted inner membrane protein |
| msrA | 1790665 | methionine sulfoxide reductase A |
| ytfM | 1790666 | predicted outer membrane protein and surface antigen |
| ytfN | 1790667 | conserved protein |
| ytfP | 1790668 | conserved protein |
| ppa | 1790673 | inorganic pyrophosphatase |
| ytfR | 48995001 | predicted sugar transporter subunit: ATP-binding component of ABC superfamily |
| ytfT | 145693214 | predicted sugar transporter subunit: membrane component of ABC superfamily |
| fbp | 1790679 | fructose-1,6-bisphosphatase I |
| mpl | 1790680 | UDP-N-acetylmuramate:L-alanyl-gamma-D-glutamyl- meso-diaminopimelate ligase |
| yjgA | 1790681 | conserved protein |
| pmbA | 1790682 | predicted peptidase required for the maturation and secretion of the antibiotic peptide MccB17 |
| nrdG | 1790685 | anaerobic ribonucleotide reductase activating protein |
| nrdD | 1790686 | anaerobic ribonucleoside-triphosphate reductase |
| treC | 1790687 | trehalose-6-P hydrolase |
| treB | 2367362 | fused trehalose(maltose)-specific PTS enzyme: IIB component/IIC component |
| treR | 1790689 | DNA-binding transcriptional repressor |
| mgtA | 2367363 | magnesium transporter |
| yjgF | 87082396 | ketoacid-binding protein |
| pyrI | 1790692 | aspartate carbamoyltransferase, regulatory subunit |
| pyrB | 2367364 | aspartate carbamoyltransferase, catalytic subunit |
| pyrL | 1790694 | pyrBI operon leader peptide |
| argI | 1790703 | ornithine carbamoyltransferase 1 |
| rraB | 1790704 | conserved protein |
| yjgM | 48995002 | predicted acetyltransferase |
| valS | 1790708 | valyl-tRNA synthetase |
| holC | 1790709 | DNA polymerase III, chi subunit |
| pepA | 1790710 | multifunctional aminopeptidase A: a cyteinylglycinase, transcription regulator and site-specific recombination factor |
| lptF | 1790712 | conserved inner membrane protein |
| lptG | 87082400 | conserved inner membrane protein |
| yjgR | 1790714 | predicted ATPase |
| idnT | 1790716 | L-idonate and D-gluconate transporter |
| yjgB | 87082401 | predicted alcohol dehydrogenase, Zn-dependent and NAD(P)-binding |
| yjiA | 87082430 | predicted GTPase |
| yjiX | 1790813 | conserved protein |
| yjiY | 87082431 | predicted inner membrane protein |
| tsr | 2367378 | methyl-accepting chemotaxis protein I, serine sensor receptor |
| opgB | 87082433 | phosphoglycerol transferase I |
| yjjA | 87082434 | conserved protein |
| dnaC | 1790823 | DNA biosynthesis protein |
| dnaT | 1790824 | DNA biosynthesis protein (primosomal protein I) |
| yjjB | 145693219 | conserved inner membrane protein |
| rsmC | 1790830 | 16S rRNA m(2)G1207 methyltransferase |
| yjjG | 1790833 | dUMP phosphatase |
| prfC | 1790835 | peptide chain release factor RF-3 |
| osmY | 1790836 | periplasmic protein |
| ytjA | 145693221 | predicted protein |
| yjjU | 1790837 | predicted esterase |
| yjjV | 87082439 | predicted DNase |
| yjjW | 1790839 | predicted pyruvate formate lyase activating enzyme |
| yjjI | 1790840 | conserved protein |
| deoC | 1790841 | 2-deoxyribose-5-phosphate aldolase, NAD(P)-linked |
| deoA | 1790842 | thymidine phosphorylase |
| deoB | 1790843 | phosphopentomutase |
| deoD | 1790844 | purine-nucleoside phosphorylase |
| lplA | 1790846 | lipoate-protein ligase A |
| ytjB | 1790847 | conserved protein |
| serB | 1790849 | 3-phosphoserine phosphatase |
| radA | 1790850 | predicted repair protein |
| nadR | 87082440 | bifunctional DNA-binding transcriptional repressor/ NMN adenylyltransferase |
| yjjK | 2367384 | fused predicted transporter subunits of ABC superfamily: ATP-binding components |
| slt | 87082441 | lytic murein transglycosylase, soluble |
| trpR | 1790854 | DNA-binding transcriptional repressor, tryptophan-binding |
| yjjX | 87082442 | thiamin metabolism associated protein |
| ytjC | 1790856 | phosphoglyceromutase 2, co-factor independent |
| rob | 1790857 | DNA-binding transcriptional activator |
| creA | 1790859 | conserved protein |
| creB | 1790860 | DNA-binding response regulator in two-component regulatory system with CreC |
| creC | 1790861 | sensory histidine kinase in two-component regulatory system with CreB or PhoB, regulator of the CreBC regulon |
| creD | 1790862 | inner membrane protein |
| arcA | 1790863 | DNA-binding response regulator in two-component regulatory system with ArcB or CpxA |
| yjjY | 1790864 | predicted protein |
| yjtD | 1790865 | predicted rRNA methyltransferase |

^a^ Gene names and annotation are taken from the genome of *E. coli* K-12 MG1655 (U00096).

^b^ Genes that are conserved in the sequenced non-*coli Escherichia* genomes are highlighted in blue.

^c^ Genes that are also conserved across all sequence enterobacterial genomes are highlighted in yellow.
