# Supplementary material for: Complete Genome Sequence and Comparative Metabolic Profiling of the Prototypical Enteroaggregative Escherichia coli Strain 042
Source: PLoS One. 2010 Jan 20;5(1):e8801. doi: 10.1371/journal.pone.0008801 (PMC2808357; doi:10.1371/journal.pone.0008801)
Supplement: Table S3 — Comparison of EAEC 042 and E. coli MG1655 metabolism by phenotype microarray, where EAEC 042 shows greater metabolic activity. (0.12 MB DOC) [file pone.0008801.s003.doc]

**Table S3.** Comparison of EAEC 042 and *E. coli* MG1655 metabolism by phenotype microarray, where EAEC 042 shows greater metabolic activity.

| **BioLog compound** | **Difference^b^** | **Mode of action** |
| --- | --- | --- |
|  |  |  |
| **Antimicrobials** |  |  |
| **Sulfisoxazole** | 602 | folate synthesis, PABA analog |
| **Sulfamonomethoxine** | 555 | folate antagonist |
| **Sulfachloropyridazine** | 542 | folate antagonist |
| **Sulfathiazole** | 371 | folate antagonist |
| **Sulfadiazine** | 367 | folate antagonist |
| **Sulfamethazine** | 305 | folate antagonist |
| **Sulfamethoxazole** | 251 | folate antagonist |
| **Chloramphenicol** | 397 | protein synthesis |
| **Thiamphenicol** | 302 | protein synthesis |
| **Spectinomycin** | 265 | protein synthesis |
| **Capreomycin** | 120 | protein synthesis |
| **Blasticidin S** | 115 | protein synthesis |
| **Dihydrostreptomycin** | 162 | protein synthesis, aminoglycoside |
| **Kanamycin** | 142 | protein synthesis; 30S ribosomal subunit; aminoglycoside |
| **Gentamicin** | 141 | protein synthesis; 30S ribosomal subunit; aminoglycoside |
| **Paromomycin** | 139 | protein synthesis, aminoglycoside |
| **Geneticin (G418)** | 138 | protein synthesis, aminoglycoside |
| **Tobramycin** | 131 | protein synthesis, aminoglycoside |
| **Sisomicin** | 127 | protein synthesis, aminoglycoside |
| **Hygromycin B** | 122 | protein synthesis, aminoglycoside |
| **Amikacin** | 88 | protein synthesis; 30S ribosomal subunit; aminoglycoside |
| **Demeclocyline** | 478 | protein synthesis; 30S ribosomal subunit; tetracycline |
| **Chlortetracycline** | 475 | protein synthesis; 30S ribosomal subunit; tetracycline |
| **Oxytetracycline** | 311 | protein synthesis, tetracycline |
| **Penimepicycline** | 311 | protein synthesis, tetracycline |
| **Tetracycline** | 283 | protein synthesis, tetracycline |
| **Rolitetracycline** | 259 | protein synthesis, 30S ribosomal subunit, tetracycline |
| **Doxycycline** | 221 | protein synthesis, tetracycline |
| **Antimony (III) chloride** | 349 | toxic cation |
| **Sodium m-arsenite** | 93 | toxic anion |
| **Potassium tellurite** | 89 | toxic anion |
| **Trifluorothymidine** | 224 | thymidylate synthetase, DNA polymerase |
| **Aminotriazole** | 163 | histidine antimetabolite biosynthesis, catalase |
| **Aminotriazole** | 163 | histidine biosynthesis, catalase |
| **Fusaric Acid** | 145 | chelator, lipophilic |
| **Apramycin** | 144 | antimicrobial, aminocyclitol |
| **Compound 48/80** | 116 | phospholipase C, ADP ribosylation |
| **Gallic Acid** | 98 | antimicrobial, from plants |
|  |  |  |
| **Carbon sources** |  |  |
| **2-Deoxy-D-Ribose** | 118 | C-source |
| **N-Acetyl-D-Galactosamine** | 115 | C-source |
| **L-Sorbose** | 108 | C-source |
| **L-Proline** | 86 | C-source |
| **N-Acetyl-D-Glucosamine** | 63 | C-source |
|  |  |  |
| **Nitrogen sources** |  |  |
| **N-Acetyl-D-Galactosamine** | 178 | N-source |
| **Val-Gln** | 130 | N-source |
| **Val-Asn** | 126 | N-source |
| **Ala-Val** | 117 | N-source |
| **Val-Ala** | 110 | N-source |
| **Asn-Val** | 108 | N-source |
| **Val-Gly** | 107 | N-source |
| **Val-Asp** | 105 | N-source |
| **Val-Arg** | 99 | N-source |
| **Gly-Val** | 98 | N-source |
| **Val-Ser** | 97 | N-source |
| **Pro-Val** | 95 | N-source |
| **Ala-Leu** | 93 | N-source |
| **Ala-Leu** | 92 | N-source |
| **Ile-Gln** | 90 | N-source |
| **Leu-Ala** | 88 | N-source |
| **Trp-Val** | 87 | N-source |
| **Val-Glu** | 85 | N-source |
| **Ser-Val** | 85 | N-source |
| **L-Glutamic Acid** | 83 | N-source |
| **Val-Pro** | 81 | N-source |
| **Asp-Val** | 81 | N-source |
| **Glu-Val** | 78 | N-source |
| **Adenine** | 77 | N-source |
| **Adenosine** | 77 | N-source |
| **Leu-Gly-Gly** | 75 | N-source |
| **Leu-Gly** | 72 | N-source |
| **Cytidine** | 71 | N-source |
| **Gly-Gly-Leu** | 71 | N-source |
| **Arg-Val** | 70 | N-source |
| **Leu-Pro** | 70 | N-source |
| **Leu-Leu** | 67 | N-source |
| **Ser-Ala** | 65 | N-source |
| **L-Arginine** | 63 | N-source |
| **Ser-Leu** | 59 | N-source |
| **Gly-Leu** | 58 | N-source |
| **L-Asparagine** | 57 | N-source |
| **Trp-Leu** | 57 | N-source |
| **N-Acetyl-D-Glucosamine** | 55 | N-source |
| **Arg-Lys** | 55 | N-source |
| **D-Glucosamine** | 54 | N-source |
| **Leu-Arg** | 54 | N-source |
| **Leu-Glu** | 54 | N-source |
| **Ala-Ser** | 54 | N-source |
| **Ile-Arg** | 53 | N-source |
| **Leu-Val** | 53 | N-source |
| **Ile-Ile** | 52 | N-source |
| **Asp-Leu** | 52 | N-source |
| **Leu-Phe** | 52 | N-source |
| **Ser-Asn** | 51 | N-source |
| **Gly-Phe-Phe** | 50 | N-source |
| **L-Valine** | 58 | nutrient stimulation |
|  |  |  |
| **Other** |  |  |
| **pH 9.5 + Putrescine** | 101 | pH, deaminase |
| **Phosphono Acetic Acid** | 70 | P-source |

^a^ Chemical compounds or growth/metabolic substrates tested in the phenotype microarrays, where there was a difference between EAEC 042 and *E. coli* MG1655

^b^ Differences between the strains are shown in arbitrary units. The average signal for each PM array well was calculated as the mean of the signal from EAEC 042 or *E. coli* MG1655 in two independent PM array experiments. The metabolic differences between the strains are shown as the arithmetic difference of the mean EAEC 042 signal minus the mean of the *E. coli* MG1655 signal for each test well. Wells where there was no difference are not shown, Wells in which there was a greater signal from EAEC 042 than *E. coli* MG1655 are shown as positive values.
