# Supplementary material for: Complete Genome Sequence and Comparative Metabolic Profiling of the Prototypical Enteroaggregative Escherichia coli Strain 042
Source: PLoS One. 2010 Jan 20;5(1):e8801. doi: 10.1371/journal.pone.0008801 (PMC2808357; doi:10.1371/journal.pone.0008801)
Supplement: Table S4 — Comparison of EAEC 042 and E. coli MG1655 metabolism by phenotype microarray, where E. coli MG1655 shows greater metabolic activity. (0.07 MB DOC) [file pone.0008801.s004.doc]

**Table S4.** Comparison of EAEC 042 and *E. coli* MG1655 metabolism by phenotype microarray, where *E. coli* MG1655 shows greater metabolic activity.

| **BioLog compound** | **Difference^b^** | **Mode of action** |
| --- | --- | --- |
|  |  |  |
| **Antimicrobials** |  |  |
| **Oxacillin** | -203 | wall, lactam |
| **Azlocillin** | -176 | wall, lactam |
| **Phenethicillin** | -155 | wall, lactam |
| **Cloxacillin** | -139 | wall; lactam |
| **Cefazolin** | -85 | wall; first generation cephalosporin |
| **Rifamycin SV** | -531 | RNA polymerase |
| **Rifampicin** | -266 | RNA polymerase |
| **Oxolinic acid** | -108 | DNA unwinding, gyrase (GN), topoisomerase (GP), quinolone |
| **Cinoxacin** | -99 | protein synthesis, quinolone |
| **Nalidixic acid** | -90 | DNA unwinding; gyrase (GN); topoisomerase (GP); quinolone |
| **Troleandomycin** | -271 | protein synthesis, macrolide |
| **Spiramycin** | -265 | protein synthesis, macrolide |
| **Josamycin** | -258 | protein synthesis, macrolide |
| **Erythromycin** | -201 | protein synthesis; 50S ribosomal subunit; macrolide |
| **Tylosin** | -126 | protein synthesis, 50S ribosomal subunit, macrolide |
| **Tetrazolium Violet** | -226 | respiration |
| **Thioridazine** | -174 | respiration |
| **Orphenadrine** | -218 | cholinergic antagonist |
| **Pridinol** | -166 | cholinergic antagonist |
| **Umbelliferone** | -338 | DNA intercalator |
| **Acriflavine** | -267 | DNA intercalator |
| **Chlorpromazine** | -197 | phenothiazine |
| **Proflavine** | -180 | RNA synthesis |
| **Promethazine** | -137 | cyclic nucleotide phosphodiesterase |
| **D-Serine** | -430 | inhibits 3PGA dehydrogenase (L-serine and pantothenate synthesis) |
| **Cytosine arabinoside** | -282 | nucleic acid analog, pyrimidine |
| **Amitriptyline** | -248 | membrane, transport |
| **5-Azacytidine** | -217 | DNA methyltransferase |
| **b-Chloro-L-Alanine** | -142 | aa analog, alanine, aminotransferase inhibitor |
| **Atropine** | -141 | acetylcholine receptor, antagonist |
| **D,L-Propranolol** | -136 | beta-adrenergic blocker |
| **Procaine** | -82 | ion channal inhibitor, Na+ (m) |
|  |  |  |
| **Carbon sources** |  |  |
| **D-Serine** | -200 | C-source |
| **Mucic Acid** | -131 | C-source |
| **b-D-Allose** | -113 | C-source |
| **a-Keto-Glutaric Acid** | -112 | C-source |
| **a-Keto-Butyric Acid** | -111 | C-source |
| **D-Xylose** | -106 | C-source |
| **5-Keto-D-Gluconic Acid** | -62 | C-source |
|  |  |  |
| **Nitrogen sources** |  |  |
| **Cys-Gly** | -72 | N-source |
| **D-Serine** | -69 | N-source |
|  |  |  |
| **Other** |  |  |
| **pH 9.5 + L-Homoarginine** | -150 | pH, deaminase |
|  |  |  |

^a^ Chemical compounds or growth/metabolic substrates tested in the phenotype microarrays, where there was a difference between EAEC 042 and *E. coli* MG1655

^b^ Differences between the strains are shown in arbitrary units. The average signal for each PM array well was calculated as the mean of the signal from EAEC 042 or *E. coli* MG1655 in two independent PM array experiments. The metabolic differences between the strains are shown as the arithmetic difference of the mean EAEC 042 signal minus the mean of the *E. coli* MG1655 signal for each test well. Wells where there was no difference are not shown. Wells where there was a smaller signal from EAEC 042 compared to *E. coli* MG1655 are shown as negative values.
