# Supplementary material for: Complete Genome Sequence and Comparative Metabolic Profiling of the Prototypical Enteroaggregative Escherichia coli Strain 042
Source: PLoS One. 2010 Jan 20;5(1):e8801. doi: 10.1371/journal.pone.0008801 (PMC2808357; doi:10.1371/journal.pone.0008801)
Supplement: Table S6 — List of EAEC 042 CDS conserved in the other sequenced EAEC genomes (101-1 and 55989), but absent from the genome of the commensal E. coli HS. (0.20 MB DOC) [file pone.0008801.s006.doc]

**Table S6.** List of EAEC 042 CDS conserved in the other sequenced EAEC genomes (101-1 and 55989), but absent from the genome of the commensal *E. coli* HS

| **CDS** | **Product** |
| --- | --- |
| **Ec042-0139** | fimbrial outer membrane usher protein |
| **Ec042-0313** | conserved hypothetical protein |
| **Ec042-0314** | putative xanthine dehydrogenase, molybdenum-binding subunit |
| **Ec042-0315** | putative xanthine dehydrogenase, FAD-binding subunit |
| **Ec042-0316** | putative xanthine dehydrogenase, iron-sulfur binding subunit |
| **Ec042-0317** | putative membrane protein |
| **Ec042-0317A** | conserved hypothetical protein |
| **Ec042-0318** | putative fimbrial protein |
| **Ec042-0319** | putative fimbrial protein |
| **Ec042-0320** | putative fimbrial outer membrane usher protein |
| **Ec042-0321** | putative fimbrial protein |
| **matB** | putative fimbrial protein |
| **Ec042-0372** | conserved hypothetical protein |
| **lacA** | galactoside O-acetyltransferase |
| **Ec042-0404** | autotransporter |
| **Ec042-0679** | conserved hypothetical protein |
| **Ec042-0680** | conserved hypothetical protein |
| **Ec042-0683** | conserved hypothetical protein |
| **Ec042-0684** | conserved hypothetical protein |
| **hscC** | chaperone protein |
| **Ec042-1191** | putative membrane protein |
| **Ec042-1192** | putative membrane protein |
| **Ec042-1312** | putative phage exodeoxyribonuclease |
| **Ec042-1313** | phage protein |
| **Ec042-1315** | putative phage protein |
| **Ec042-1317** | putative phage protein |
| **Ec042-1323** | putative phage regulatory protein |
| **Ec042-1324** | putative phage regulatory protein |
| **Ec042-1328** | putative phage protein |
| **Ec042-1329** | putative phage protein |
| **Ec042-1333** | phage protein |
| **Ec042-1341** | putative phage protein |
| **Ec042-1342** | phage protein |
| **Ec042-1345** | putative phage protein |
| **Ec042-1346** | phage DNA adenine-methylase |
| **Ec042-1351** | putative phage lysozyme |
| **Ec042-1352** | putative phage protein |
| **Ec042-1353** | putative phage protein |
| **Ec042-1354** | putative phage protein |
| **Ec042-1355** | putative phage protein |
| **Ec042-1356** | phage terminase small subunit |
| **Ec042-1357** | phage terminase large subunit |
| **Ec042-1365** | phage minor tail protein |
| **Ec042-1366** | phage minor tail protein |
| **Ec042-1367** | phage major tail protein |
| **Ec042-1368** | phage minor tail protein |
| **Ec042-1369** | phage minor tail protein |
| **Ec042-1370** | phage minor tail protein |
| **Ec042-1371** | phage minor tail protein |
| **Ec042-1372** | phage minor tail protein |
| **Ec042-1373** | phage tail assembly protein |
| **Ec042-1374** | phage tail assembly protein |
| **Ec042-1380** | putative phage protein |
| **Ec042-1381** | phage tail fibre assembly protein |
| **Ec042-1400A** | conserved hypothetical protein |
| **Ec042-1472** | putative phage protein |
| **Ec042-1480** | putative phage protein |
| **Ec042-1481** | putative phage protein |
| **Ec042-1484** | phage protein |
| **Ec042-1487** | phage lysozome |
| **Ec042-1488** | phage endopeptidase/lysis protein |
| **Ec042-1509** | putative phage minor tail protein |
| **Ec042-1510** | putative phage tail assembly protein |
| **Ec042-1511** | putative phage tail assembly protein |
| **Ec042-1637** | fimbrial outer membrane usher protein |
| **Ec042-1655** | putative signal transduction protein |
| **Ec042-1682** | phage tail assembly protein |
| **Ec042-1683** | putative phage minor tail protein |
| **Ec042-1684** | phage minor tail protein |
| **Ec042-1685** | phage minor tail protein |
| **Ec042-1688** | phage minor tail protein |
| **Ec042-1689** | phage minor tail protein |
| **Ec042-1690** | phage major tail protein |
| **Ec042-1691** | phage minor tail protein |
| **Ec042-1692** | phage minor tail protein |
| **Ec042-1699** | phage terminase large subunit (DNA packaging protein) |
| **Ec042-1700** | phage terminase small subunit (DNA packaging protein) |
| **Ec042-1702** | putative conserved hypothetical protein |
| **Ec042-1703** | conserved hypothetical protein |
| **Ec042-1705** | putative prophage protein |
| **Ec042-1705A** | putative prophage protein |
| **Ec042-1714** | putative prophage protein |
| **Ec042-1715** | putative phage replication protein |
| **Ec042-1716** | putative phage protein |
| **dicC** | repressor protein of division inhibition gene |
| **dicA** | repressor protein of division inhibition gene |
| **Ec042-1720** | putative prophage protein |
| **Ec042-1721** | putative prophage protein |
| **dicB** | division inhibition protein |
| **Ec042-1723** | putative prophage protein |
| **Ec042-1724** | putative exodeoxyribonuclease |
| **Ec042-1725** | putative phage excisionase |
| **int** | integrase |
| **Ec042-1863** | AraC-family transcriptional regulator |
| **Ec042-1864** | putative electron transfer flavoprotein beta subunit |
| **Ec042-1865** | putative electron transfer flavoprotein alpha subunit |
| **fadK** | short-chain-fatty-acid--CoA ligase (acyl-CoA synthetase) |
| **Ec042-1975** | conserved hypothetical protein |
| **Ec042-2120** | putative membrane protein |
| **Ec042-2121** | outer membrane protein |
| **Ec042-2136** | putative prophage tail assembly protein |
| **Ec042-2137** | putative prophage tail assembly protein |
| **Ec042-2138** | prophage minor tail protein |
| **Ec042-2174** | putative prophage endopeptidase |
| **Ec042-2183** | putative prophage protein |
| **Ec042-2184** | putative prophage protein |
| **Ec042-2189** | putative prophage protein |
| **Ec042-2192** | putative prophage protein |
| **Ec042-2193** | putative prophage protein |
| **Ec042-2196** | putative prophage protein |
| **Ec042-2200** | putative prophage protein |
| **Ec042-2201** | putative prophage protein |
| **Ec042-2202** | putative prophage protein |
| **Ec042-2245** | conserved hypothetical protein |
| **Ec042-2246** | putative antirestriction protein |
| **Ec042-2247** | putative DNA repair protein |
| **Ec042-2247A** | conserved hypothetical protein |
| **Ec042-2251** | conserved hypothetical protein |
| **wzzB** | chain length determinant protein |
| **Ec042-2276** | putative mannose-1-phosphate guanylyltransferase |
| **wcaM** | colanic acid biosynthesis protein |
| **wcaL** | putative colanic acid biosynthesis glycosyltransferase |
| **wcaK** | putative colanic acid biosynthesis pyruvyl transferase |
| **wzxC** | putative flippase (Putative export protein) |
| **wcaJ** | putative colanic biosynthesis UDP-glucose lipid carrier transferase |
| **wcaI** | putative colanic acid biosynthesis glycosyl transferase |
| **nudD** | GDP-mannose mannosyl hydrolase) |
| **fcl** | GDP-L-fucose synthetase |
| **gmd** | GDP-mannose 4,6-dehydratase |
| **wcaF** | putative colanic acid biosynthesis acetyltransferase |
| **wcaE** | putative colanic acid biosynthesis glycosyl transferase |
| **wcaD** | putative colanic acid polymerase |
| **wcaC** | putative glycosyltransferase |
| **wcaB** | putative acetyltransferase |
| **wcaA** | putative glycosyl transferase |
| **wzc** | tyrosine-protein kinase |
| **wzb** | protein-tyrosine phosphatase |
| **Ec042-2513** | putative peptidase |
| **cmtB** | mannitol-specific cryptic PTS system IIIA component |
| **Ec042-3174** | integrase |
| **Ec042-3188** | transposase (partial) |
| **insI** | transposase InsI for insertion sequence element IS30b/c/d |
| **Ec042-3213** | transposase |
| **Ec042-3221** | conserved hypothetical protein |
| **Ec042-3222** | putative antirestriction protein |
| **Ec042-3223** | putative DNA repair protein |
| **Ec042-3224** | conserved hypothetical protein |
| **Ec042-3227** | conserved hypothetical protein |
| **Ec042-3228** | conserved hypothetical protein |
| **Ec042-3229** | conserved hypothetical protein |
| **Ec042-3256** | conserved hypothetical protein |
| **ttdR** | LysR-family transcriptional regulator |
| **Ec042-3772** | conserved hypothetical protein |
| **Ec042-3773** | conserved hypothetical protein |
| **Ec042-3774** | putative membrane protein |
| **bcsC** | cellulose synthase operon protein C (TPR-repeat-containing protein) |
| **bcsZ** | endo-1,4-beta-glucanase (cellulase) |
| **bcsB** | cyclic di-GMP-binding protein (cellulose synthase regulator subunit B) |
| **bcsA** | cellulose synthase catalytic subunit [UDP-forming] |
| **Ec042-3834** | conserved hypothetical protein |
| **Ec042-3835** | conserved hypothetical protein |
| **Ec042-3836** | conserved hypothetical protein |
| **Ec042-3837** | putative membrane protein |
| **Ec042-3838** | putative membrane protein |
| **Ec042-4034** | putative membrane protein |
| **Ec042-4035** | putative sulfatase |
| **Ec042-4036** | putative sodium:solute symporter |
| **Ec042-4037** | AraC-family transcriptional regulator |
| **rbsR** | ribose operon repressor |
| **Ec042-4500** | conserved hypothetical protein |
| **Ec042-4504** | conserved hypothetical protein |
| **Ec042-4505** | putative plasmid-related protein |
| **Ec042-4506** | putative plasmid-related protein |
| **Ec042-4507** | conserved hypothetical protein |
| **Ec042-4562** | conserved hypothetical protein |
| **Ec042-4563** | conserved hypothetical protein |
| **Ec042-4571** | conserved hypothetical protein |
| **Ec042-4579A** | transposase |
| **Ec042-4581A** | transposase (partial) |
| **Ec042-4581B** | conserved hypothetical protein |
| **Ec042-4584** | transposase |
| **Ec042-4587** | putative transposase |
| **Ec042-4592** | transposase |
| **Ec042-4597** | transposase |
| **Ec042-4610** | integrase |
| **Ec042-4741** | integrase |
| **Ec042-4752** | putative transposase (partial) |
| **Ec042-4763** | transposase for insertion sequence IS100 |
| **Ec042-4764** | insertion sequence IS100, ATP-binding protein |
| **Ec042-4766** | putative membrane protein |
| **betU** | secondary glycine betaine transporter |
| **Ec042-4768** | transposase (pseudogene) |
| **virK** | virulence protein |
| **Ec042-4771** | glycosyl transferase |
| **Ec042-4772** | putative polysaccharide deacetylase |
| **Ec042-4775** | conserved hypothetical protein (partial) |
| **fecE** | iron(III) dicitrate transport system, ATP-binding protein |
| **fecD** | iron(III) dicitrate transport system, permease protein |
| **fecC** | iron(III) dicitrate transport system, permease protein |
| **fecB** | iron(III) dicitrate-binding periplasmic protein |
| **fecA** | iron(III) dicitrate TonB-dependent receptor |
| **fecR** | iron(III) dicitrate sensor protein |
| **fecI** | RNA polymerase sigma factor |
| **Ec042-4786A** | transposase |
| **Ec042-4804** | conserved hypothetical protein (partial) |
| **Ec042-4805** | conserved hypothetical protein |
| **Ec042-4807** | putative RadC-like DNA repair protein |
| **Ec042-4808** | conserved hypothetical protein |
| **Ec042-4809** | conserved hypothetical protein |
| **Ec042-4814A** | conserved hypothetical protein (partial) |
