# Supplementary material for: Complete Genome Sequence and Comparative Metabolic Profiling of the Prototypical Enteroaggregative Escherichia coli Strain 042
Source: PLoS One. 2010 Jan 20;5(1):e8801. doi: 10.1371/journal.pone.0008801 (PMC2808357; doi:10.1371/journal.pone.0008801)
Supplement: Table S7 — Type 3 secretion system effector genes in the EAEC 042 genome. (0.05 MB DOC) [file pone.0008801.s007.doc]

**Table S7: Type 3 secretion system** effector genes in the EAEC 042 genome.

| **Effector** | **EAEC 042 CDS** | **Sakai CDS** | **EDL933 CDS** | **Family** | **Homolog** | **Comments** |
| --- | --- | --- | --- | --- | --- | --- |
| EspX1 | Ec042-0025 | ECs0025 | z0025 | PPR | SopA |  |
| EspY1 | Ec042-0060 | ECs0061 | z0065 | SopD_Nterm | SopD |  |
| EspY2 | Ec042-0072 | ECs0073 | z0078 | SopD_Nterm | NleO2 |  |
| EspY3 | Ec042-0455 | ECs0472 | z0521 | SopD_Nterm; PRR | SopD |  |
| EspR1 | Ec042-1599 | ECs2073 | z2242 | LRR | IpaH |  |
| EspR2 | Ec042-1600 | ECs2074  ECs2075 | z2241/  z2240 | LRR | IpaH | intact in EAEC 042; pseudogene in EHEC |
| EspL1 | Ec042-1886 | ECs2427 | z2749 | AR | OspD |  |
| EspR3 | Ec042-2094 | ECs2672/4 | z3023/6 | LRR | IpaH | one gene in EAEC 042 where two in EHEC |
| EspL3' | Ec042-4064 | ECs4642/3 | z5200/  z5199 | AR | OspD | intact in EAEC 042; pseudogene in EHEC |
| EspY4 | Ec042-4073 | ECs4653 | z5211 | SopD_Nterm | SopD |  |
| EspX3 | Ec042-4074 | ECs4654/5 | z5212/3 | PPR | SopA | intact in EAEC 042; pseudogene in EHEC |
| EspY5 | Ec042-4075 | ECs4657 | z5214 | SopD_Nterm | SopD | intact in EAEC 042; pseudogene in EHEC |
| EspL4 | Ec042-4379 | ECs4935 | z5608 | AR | OspD |  |
| EspX4 | Ec042-4407 | ECs5021 | z5636 | PPR | SopA |  |
| EspX5 | Ec042-4436 | ECs5048 | z5665 | PPR | PipB | pseudogene in EAEC 042; intact in EHEC |
