# Supplementary figures and images for: Complete Genome Sequence and Comparative Metabolic Profiling of the Prototypical Enteroaggregative Escherichia coli Strain 042
Source: PLoS One. 2010 Jan 20;5(1):e8801. doi: 10.1371/journal.pone.0008801 (PMC2808357; doi:10.1371/journal.pone.0008801)

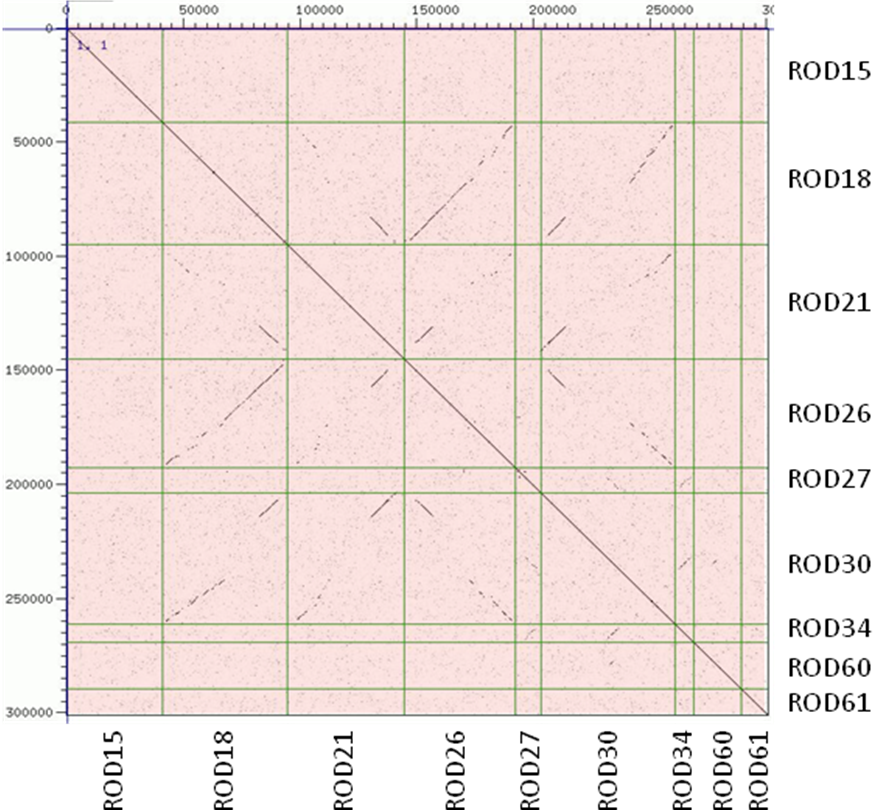

Supplement: Figure S1 — Genetic similarity of the EAEC 042 prophage. Nine prophage regions, designated 042p1–042p9, were identified in the EAEC 042 genome (see Table 2 in manuscript). The figure represents homology of the phage elements; solid lines indicate complete identity, the absence of lines within the boxes reflect little or no homology and the presence of dashed lines indicates partial identity. Four of these were lambdoid in nature (042p2, 042p3, 042p4 and 042p6) and were highly similar to each other and to the lambdoid prophages of E. coli O157:H7 [1], and E. coli O127:H6 strain E2348/69 (EPEC) [2]. Some of the related lambdoid prophages in EAEC 042 and E. coli O157:H7 are integrated in corresponding genomic locations and share some sequence identity; 042p3 and Sp10, 042p4 and Sp11/Sp12, and 042p6 and Sp14 are found relative to each other in the respective bacterial genomes. The EAEC 042 lambda-like prophages also have high homology to lambdoid prophages in several of the other sequenced pathogenic E. coli genomes including avaian pathogenic (APEC), uropathogenic (UPEC) and enterotoxigenic (ETEC). In addition, 042p4 has high homology to the K-12 cryptic prophage Qin and genome comparison shows where the deletions have occurred in the Qin genome to render it defective. 042p5 and 042p7 have some sequence similarity to O157:H7 Sakai prophage Sp7 which is of an unstudied type [1], [3]. Half of prophage 042p1, from the non-tail end to the lysis module, is related to lambda, P22 and PP8 in EPEC, but the remainder of the prophage, which encodes mainly structural proteins, is highly related to the Shigella flexneri serotype converting phage SfV [4]. 042p1 encodes bactoprenol glucosyl transferase (GtrB) and glucose translocase (GtrA), at the very end of the prophage, after the tail genes. These proteins are involved in O-antigen modification and have homology to the serotype-converting proteins of SfV (87% protein identity to GtrA and GtrB) and P22 (88% protein identity to GtrA and 77% to [file pone.0008801.s008.doc]

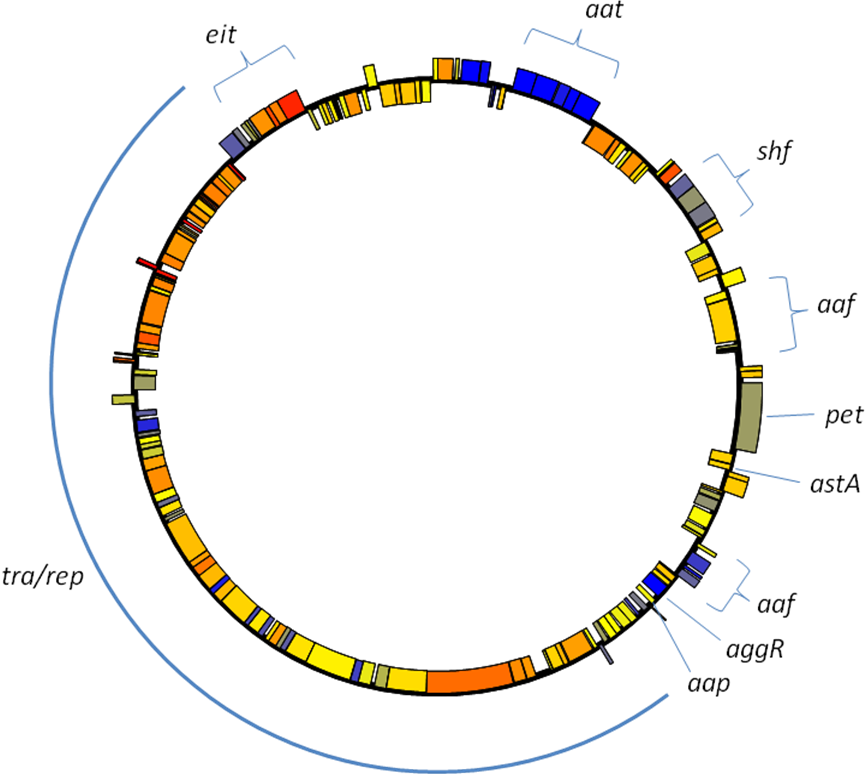

Supplement: Figure S2 — Genetic map of pAA, the large virulence plasmid of EAEC 042. (A) On the basis of nucleotide sequence homology, the plasmid pAA belongs to the IncFIIA family and carries just one identifiable replicon consisting of Ec042-pAA152 (RepA) and Ec042-pAA153 (CopB), which is in contrast to many of the F-family plasmids that have multiple replicons. It possesses auxiliary stable inheritance functions including Ec042-pAA136–137 encoding the type 2 partitioning proteins ParM and ParR, a Hok/Sok post-segregational killing system (Ec042-pAA106) and the putative pair of Ec042-pAA147–148 encoding a RelE/StbE homologue. As for most IncFII plasmids, pAA appears to encode a complete F-like conjugative transfer system (Ec042-pAA066–100), most closely related to those of pUT189 and R100, and does not seem to lack any standard component which might explain why attempts to transfer a derivative tagged with an antibiotic resistance marker (IH, unpublished) have been unsuccessful. However, analysis of the predicted gene products identified two CDS with unusual features that might be worth investigating as the basis of a transfer defect: TraP (Ec042-pAA092) shows N-terminal segments differing significantly from the nearest relatives despite high overall sequence alignment; and TrwB (Ec042-pAA069) contains an internal region towards the end of the protein with a significant amplification of a run of PQQP repeats which may have caused it to become non-functional. Functional analyses are needed to determine whether these features could be responsible for the Tra- phenotype. Other transfer-associated genes are: Ec042-pAA129 encoding a putative anti-restriction gene; Ec042-pAA113 encoding Ssb (single stranded DNA binding protein); Ec042-pAA109–110 encoding the SOS-induced response proteins PsiA/B associated with plasmid transfer; and Ec042-pAA101 encoding a lytic transglycosylase that helps hydrolyse cell wall in recipients prior to conjugative transfer. With respect to overall organisation it i [file pone.0008801.s009.doc]

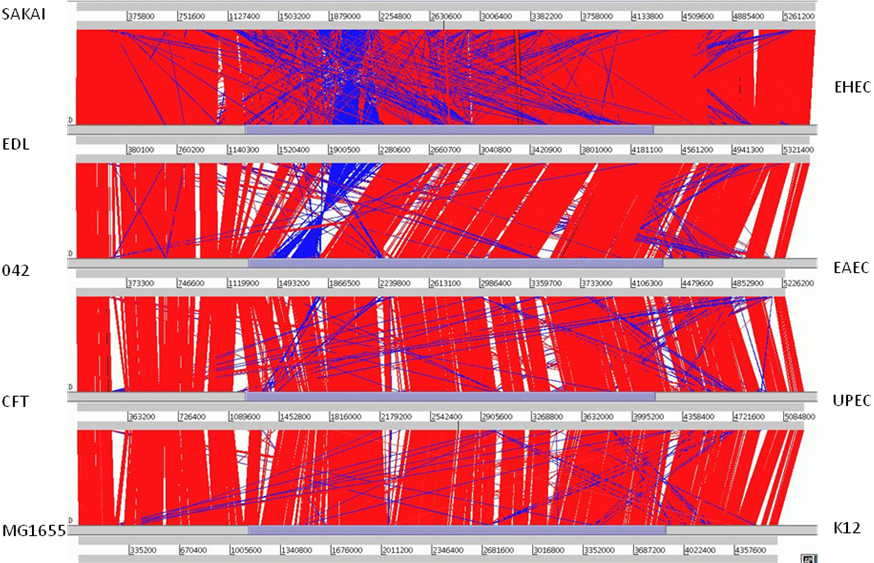

Supplement: Figure S3 — Global comparison between EAEC 042 chromosome and those of EHEC, UPEC and K-12. ACT comparison (http://www.sanger.ac.uk/Software/ACT) of amino-acid matches between the complete six-frame translations (computed using TBLASTX) of the whole genome sequences of enterohaemorrhagic E. coli O157:H7 str. Sakai (EHEC; EMBL acc: BA000007), enterohaemorrhagic E. coli O157:H7 EDL933 (EHEC; EMBL acc: AE005174), uropathogenic E. coli strain CFT073 (UPEC; EMBL acc: AE014075), and E. coli strain K-12 MG1655 (K-12; EMBL acc: U00096). Forward and reverse strands of DNA are shown for each genome (dark grey lines). The red bars between the DNA lines represent individual TBLASTX matches, with inverted matches coloured blue. (1.37 MB DOC) [file pone.0008801.s010.doc]

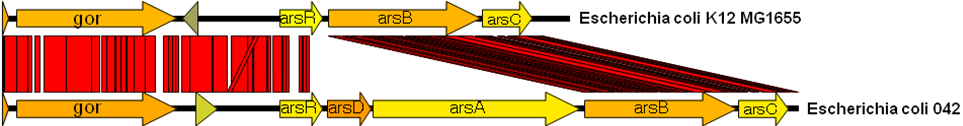

Supplement: Figure S7 — Alignment of the ars operon from of E. coli K12 with EAEC 042. Alignments reveal genetic lesions in the E. coli K12 locus which result in the lack of the arsD and arsA genes. The loss of these genes confer upon E. coli K12 a reduced ability to grow in the presence of arsenite and antimony chloride. (0.12 MB DOC) [file pone.0008801.s014.doc]

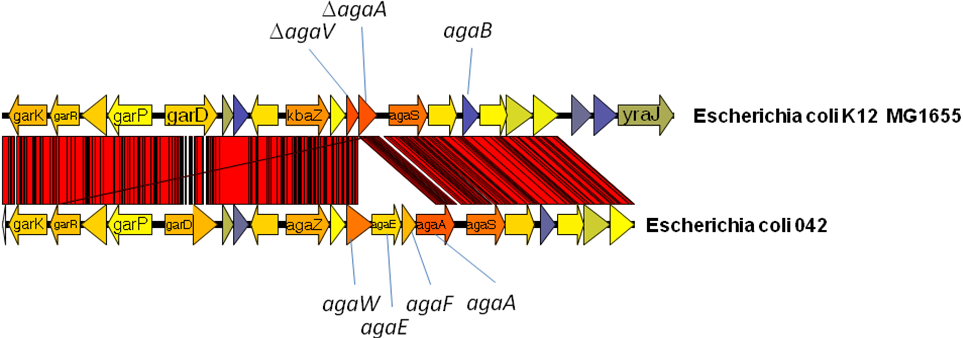

Supplement: Figure S8 — Alignment of the EAEC 042 and E. coli MG1655 aga gene clusters. E. coli MG1655 lacks the agaE and agaF genes and has a truncated agaA gene. These truncations result in decreased N-acetyl-D-galactosamine and N-acetyl-D-glucosamine utilisation. (0.22 MB DOC) [file pone.0008801.s015.doc]

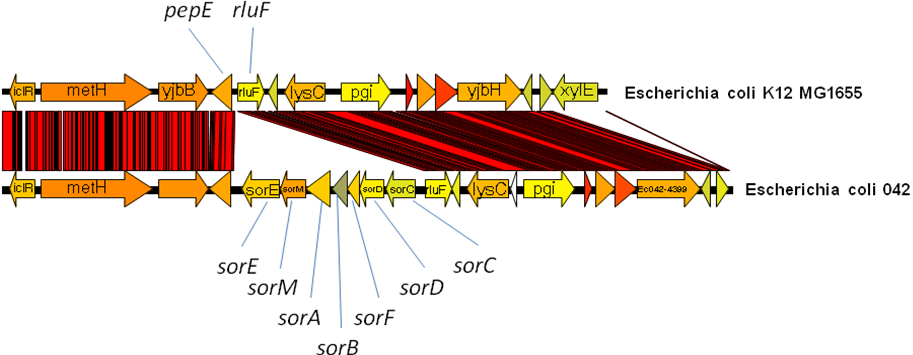

Supplement: Figure S9 — The sor operon of EAEC 042. EAEC 042 possesses the sor operon and has the ability to utilise sorbose as a sole carbon source. E. coli K-12 lacks the operon and can not utilise this carbon source, as confirmed by the BioLog phenotyping arrays. (0.22 MB DOC) [file pone.0008801.s016.doc]

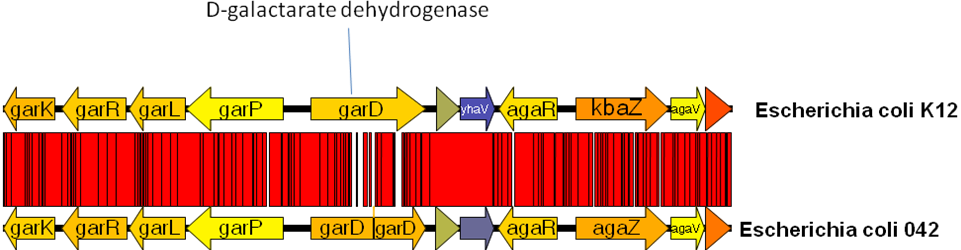

Supplement: Figure S10 — Comparison of the genomic region from E. coli K-12 and EAEC 042 encoding garD. The EAEC 042 gene is disrupted whereas it is uninterrupted in E. coli K-12. Disruption of garD resulted in the inability of EAEC 042 to utilise D-galactarate as a sole carbon source. E. coli K12 can utilise this substrate. (0.15 MB DOC) [file pone.0008801.s017.doc]

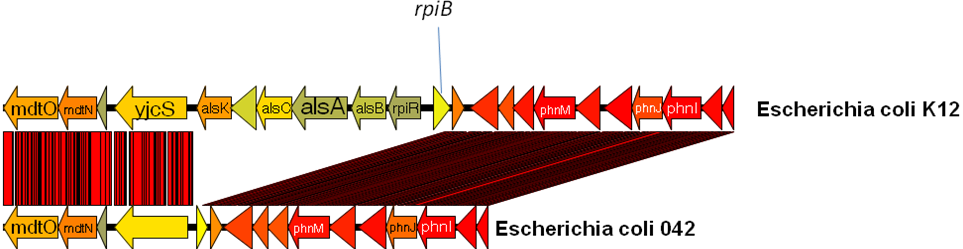

Supplement: Figure S11 — Genetic architecture of the region encoding the als locus from E. coli K-12 and the similar region from EAEC 042. The als locus is absent from EAEC 042 explaining the inability of EAEC 042 to utilise allose as a sole carbon source; E. coli K12 is capable of using allose as a sole carbon source. This prediction was confirmed by BioLog PMs. (0.20 MB DOC) [file pone.0008801.s018.doc]

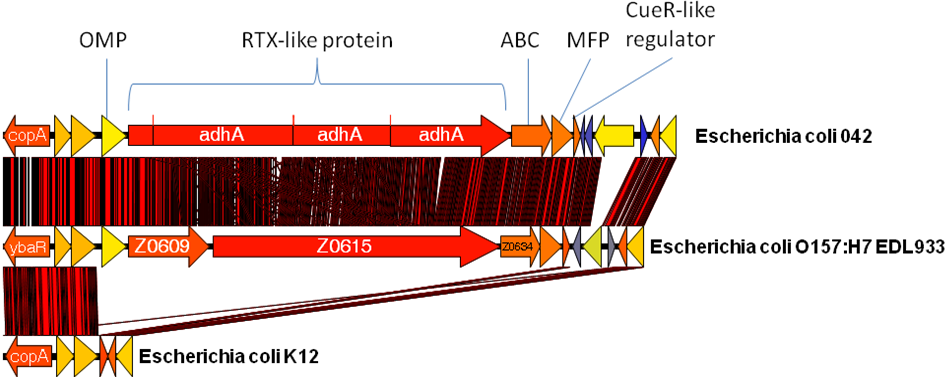

Supplement: Figure S14 — Genetic architecture of the RTX-like Type 1 secretion locus. An alignment of the genomic regions encompassing the T1SS is depicted, demonstrating the absence of the locus in E. coli K12 and the presence of the locus in EAEC 042 and E. coli O157:H7. In both pathogenic strains the gene encoding the putative secreted RTX-like protein is frameshifted and would be predicted to encode a non-functional protein. The frameshifts occur at different positions within the respective genes; in EAEC 042 there are 3 frameshifts whereas in E. coli O157:H7 there is only one frameshift. The position of the OMP, ABC, MFP and a putative regulator are indicated. (0.40 MB DOC) [file pone.0008801.s021.doc]

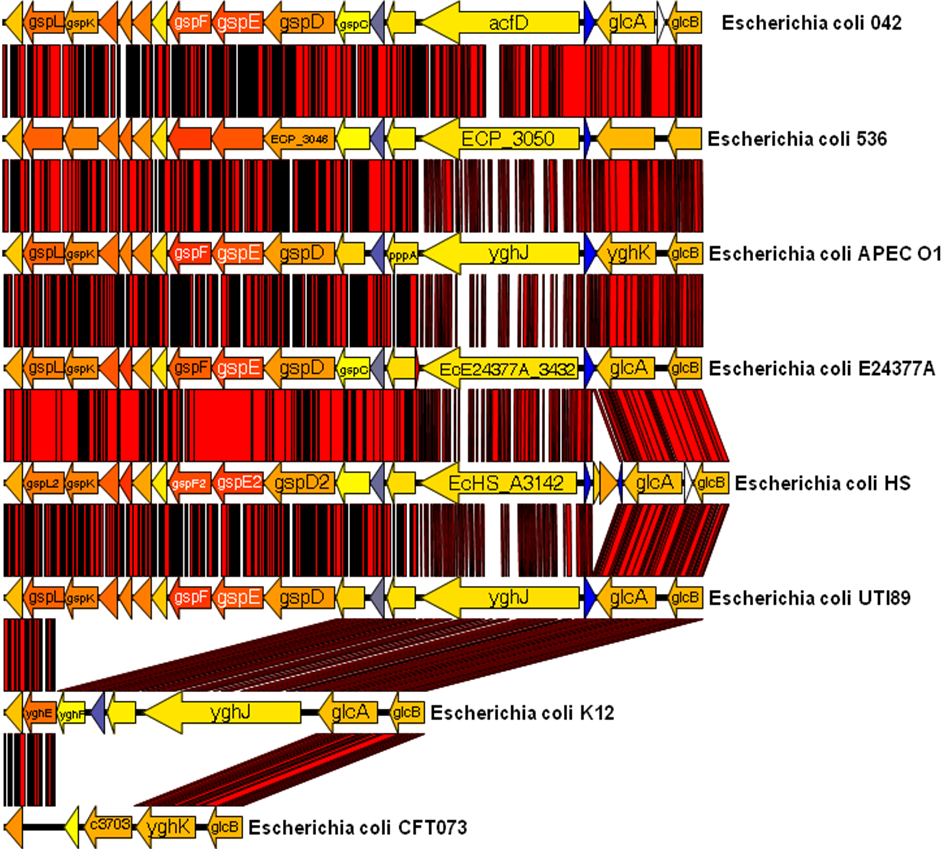

Supplement: Figure S15 — Genomic architecture of the Type II secretion system (T2SS) apparatus of EAEC 042 and representative E. coli strains. The T2SS locus (gsp) appears intact in EAEC 042 and a variety of phylogenetically disparate strains of pathogenic E. coli. In contrast, the locus possesses a lesion in E. coli K12 and is absent in E. coli CFT073. Where the locus is intact it is always syntenic with yghJ a gene encoding a putative secreted lipoprotein. (0.88 MB DOC) [file pone.0008801.s022.doc]

*aaiA*

*aaiG*

*aaiP*


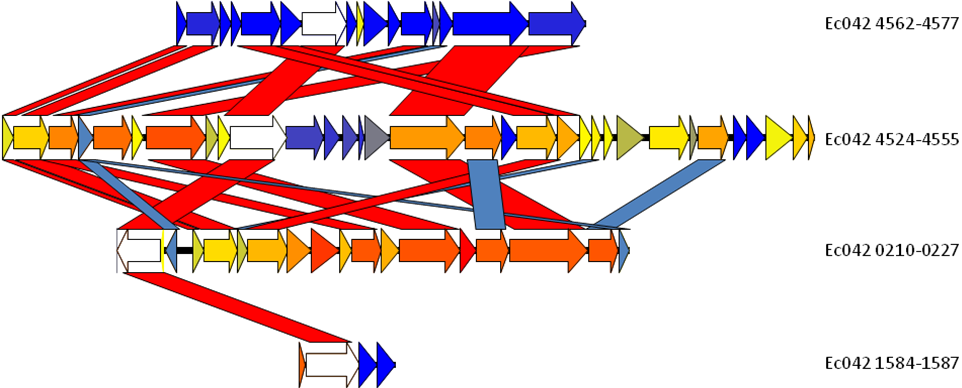

Supplement: Figure S17 — Comparison of three type VI secretion systems (T6SSs) of EAEC 042, and a separate Vgr-encoding region. Genes depicted by the schematic are indicated by the “Ec042” numbers on the right of the figure. Genes are designated by arrows, which are colored by xBASE to correspond to GC content, except for Hcp- and Vgr-homologs, which are shown in purple and white, respectively. Red blocks connecting genes indicate significant (P<0.05) identity on the amino acid level among homologs in all three T6SSs. Blue connectors indicate homologs that were only found within two T6SSs. The locus labeled Ec042-1584–1588 depicts a chromosomal region that encodes a Vgr-homolog, but is not in proximity to the other T6SSs. One of the T6SS identified (Ec042-4562–4577) had been previously characterized [7] as a locus under the control of AggR, the master regulator of virulence in this strain. The genes are part of a 117-kb pathogenicity island that is inserted at the pheU locus. This T6SS does not encode an Hcp homolog [7], however it is speculated that the third gene in this locus, previously designated aaiC, is a functional homolog. BLASTN results of this entire locus indicate that a nearly identical 18.4 kb sequence is found in enteroaggregative E. coli (EAEC) strain 55989 [8]. This is consistent with the previous report [7] that homologs of aaiC and the first gene of this locus designated aaiA are widely distributed among strains of EAEC. Unlike Hcp from other organisms, which is found widely within T6SSs [9], homologs of AaiC were only found within putative T6SSs of the atypical EAEC strain 101-1 (33% amino acid identity; GenBank AAMK02000010) and Citrobacter youngae ATCC 29220 (30% amino acid identity; GenBank EEK20055). Therefore, the function of this T6SS may be specific to the pathogenesis of EAEC and a limited number of other pathogens. (0.21 MB DOC) [file pone.0008801.s024.doc]

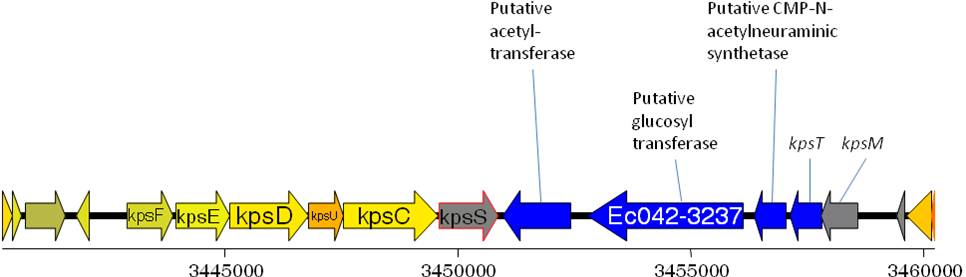

Supplement: Figure S18 — Genetic architecture of the capsular polysaccharide locus from EAEC 042. The locus is required for the biosynthesis of a group 2 capsular polysaccharide and appears to contain all the functional genes necessary for capsular production. Three CDS are present in the central region 2 and are likely to be important in the biosynthesis of the particular capsular polysaccharide expressed by this strain of E. coli. The ORF Ec042-3238 had 33% identity and 53% homology over 202 amino acids to CMP-N-acetylneuraminic acid synthetase enzymes from a number of bacterial species. This enzyme catalyses the conversion of CTP and neuraminic (NeuNAc) acid to form CMP-NeuNAc a key sugar activation step for the subsequent incorporation of NeuNAc into polysaccharides. At this stage in the absence of biochemical data one cannot be unequivocal about the enzymatic activity of the encoded protein but one can be sure it is involved in the activation of a nine-carbon sugar, which may be NeuNAc. Gene Ec042-3237 is predicted to encode a large protein of 1113 amino acids and has significant homology to the HAD super family of hydrolase enzymes and a number of putative glycosyl transferases from Salmonella enterica. This analysis would suggest a role in polysaccharide biosynthesis but in the absence of any structural data for the capsular polysaccharide expressed by this strain assigning a precise function to this protein is difficult. The remaining ORF in region 2 Ec042-3236 has significant homology (50% over 206 amino acids) with a putative acetyl-transfersae from Neisseria meningitidis and less homology to similar acetyl-transferases from a number of other bacteria. One possibility is that this is an acetyl-transferase that acetylates the capsular polysaccharide of this strain. Acetylation of the K1 polysaccharide of E. coli is known to occur and has been suggested as a mechanism by which the antigenicity of the cell surface polysaccharide may be modified in a stochastic fashion [10]. Analysis [file pone.0008801.s025.doc]

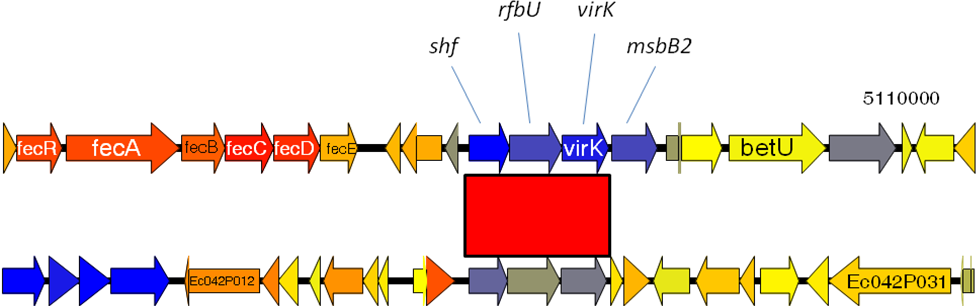


**A**

**B**

Supplement: Figure S19 — Comparison of the EAEC 042 chromosomal- and plasmid-based copies of the shf loci. The sequences surrounding the loci are not homologous. The ca. 3 kb of nucleotide sequence encompassing shf, rfbU and virK is identical; msbB2 is absent from the plasmid copy but present in the chromosomal copy. The similarity of Shf to the Staphylococcus epidermidis protein IcaB, which is required for exopolysaccharide modification and biofilm formation, indicated that Shf might also play a role in polysaccharide modification, a hypothesis supported by the presence of a polysaccharide deacetylase domain within Shf [14]. Recently, RfbU was discovered to be responsible for the addition of α-1,7-GlcN to the R3 core region of LPS and has been renamed WabB [15]. The R3 core is found in a variety of pathogenic E. coli and Shigella and is of significant biomedical interest. The function of VirK remains elusive. Initially described in Shigella, where it was found to be necessary for localisation of the autotransporter IcsA to the bacterial surface and subsequent intracellular spreading [16], VirK has been characterised in Salmonella where it has also been shown to be important for virulence [17]. MsbB2 was recently shown to act as a myristoyl transferase which modifies the lipid A portion of LPS and acts in a manner analogous to the chromosomal gene lpxM [18]. Furthermore, several investigations have demonstrated the importance of this gene for full virulence in Shigella and E. coli. Deletion of both msbB2 and lpxM results in altered membrane fatty acid composition and susceptibility to a variety of antibiotics and detergents suggesting defects in membrane biogenesis [19]. (0.15 MB DOC) [file pone.0008801.s026.doc]
