# Supplementary material for: Complete Genome Sequence and Comparative Metabolic Profiling of the Prototypical Enteroaggregative Escherichia coli Strain 042
Source: PLoS One. 2010 Jan 20;5(1):e8801. doi: 10.1371/journal.pone.0008801 (PMC2808357; doi:10.1371/journal.pone.0008801)
Supplement: Figure S12 — Sequence alignment of the E. coli K-12 and EAEC 042 porins. All the porins identified in EAEC 042 were aligned with those from E. coli K12 MG1655. NmpC (OmpD) is a pseudogene in E. coli K12 characterised by a C-terminal deletion. Identical residues are marked by asterisks, whereas similar residues are marked by periods and colons. The amino acid sequences of the OmpF, OmpC, PhoE and OmpN alleles from EAEC 042 and E. coli MG1655 are relatively well conserved (see Figure 4 in manuscript). In contrast, the OmpD proteins are more divergent and Ec042-2121 is sufficiently divergent to represent an apparently different lineage. Such divergence may be indicative of additional functions. (0.22 MB DOC) [file pone.0008801.s019.doc]

10 20 30 40 50 60 70 80 90 100

....|....|....|....|....|....|....|....|....|....|....|....|....|....|....|....|....|....|....|....|

**Ec042-1020**  **MKRNILAV--IVPALLVAGTANAAEIYNKDGNKVDLYGKAVGLHYFS---KDNGVNSYGGNGDKTYARLGFKGETQINSDLTGYGQWEYNFQGNNSEGAD**

**ompF**  **MKRNILAV--IVPALLVAGTANAAEIYNKDGNKVDLYGKAVGLHYFS---KGNGENSYGGNGDMTYARLGFKGETQINSDLTGYGQWEYNFQGNNSEGAD**

**Ec042-0302**  **MKKSTLAL--VVMGIVASASVQAAEIYNKDGNKLDVYGKVKAMHYMS---DNDSKD-----GDQSYIRFGFKGETQINDQLTGYGRWEAEFAGNKAESDT**

**phoE**  **MKKSTLAL--VVMGIVASASVQAAEIYNKDGNKLDVYGKVKAMHYMS---DNASKD-----GDQSYIRFGFKGETQINDQLTGYGRWEAEFAGNKAESDT**

**Ec042-1601**  **MKLKIVAV--VVTGLLAANVAHAAEVYNKDGNKLDLYGKVTALRYFT---DDKRDD-----GDKTYARLGFKGETQINDQMIGFGHWEYDFKGYNDEANG**

**nmpC**  **MKKLTVAISAVAASVLMAMSAQAAEIYNKDSNKLDLYGKVNAKHYFS---SNDADD-----GDTTYARLGFKGETQINDQLTGFGQWEYEFKGNRAESQG**

**Ec042-1523**  **MKSKVLAL--LIPALLAAGAAHAAEVYNKDGNKLDLYGKVDGLHYFS---DNSAKD-----GDQSYARLGFKGETQINDQLTGYGQWEYNIQANNTESSK**

**ompN**  **MKSKVLAL--LIPALLAAGAAHAAEVYNKDGNKLDLYGKVDGLHYFS---DNSAKD-----GDQSYARLGFKGETQINDQLTGYGQWEYNIQANNTESSK**

**Ec042-2456**  **MKVKVLSL--LVPALLVAGAANAAEVYNKDGNKLDLYGKVDGLHYFS---DNKSED-----GDQTYVRLGFKGETQVTDQLTGYGQWEYQIQGNTSEDNK**

**ompC**  **MKVKVLSL--LVPALLVAGAANAAEVYNKDGNKLDLYGKVDGLHYFS---DNKDVD-----GDQTYMRLGFKGETQVTDQLTGYGQWEYQIQGNSAE-NE**

**Ec042-2121**  **MKRKVLAM--LVPALLVAGAANAAEIYNKDGNKVDFYGKMVGERIWSNTDDNNSEN-----EDTSYARFGVKGETQITSELTGFGQFEYNLDASKPEGEN**

**Clustal Consensus** **** ::: : .:: : .:***:****.**:*.*** . : : .. : * :* *:*.*****:..:: *:*::* :: . ***

110 120 130 140 150 160 170 180 190 200

....|....|....|....|....|....|....|....|....|....|....|....|....|....|....|....|....|....|....|....|

**Ec042-1020**  **AQKGNKTRLAFAGLKFADAGSIDYGRNYGVVYDALGYTDMLPEFGGDTAY-SDDFFVGRVGGVATYRNSNFFGLVDGLNFAVQYLGKN------------**

**ompF**  **AQTGNKTRLAFAGLKYADVGSFDYGRNYGVVYDALGYTDMLPEFGGDTAY-SDDFFVGRVGGVATYRNSNFFGLVDGLNFAVQYLGKN------------**

**Ec042-0302**  **AQQ--KTRLAFAGLKYKDLGSFDYGRNLGALYDVEAWTDMFPEFGGDSSAQTDNFMTKRASGLATYRNTDFFGVIDGLNLTLQYQGKN------------**

**phoE**  **AQQ--KTRLAFAGLKYKDLGSFDYGRNLGALYDVEAWTDMFPEFGGDSSAQTDNFMTKRASGLATYRNTDFFGVIDGLNLTLQYQGKN------------**

**Ec042-1601**  **SRGN-KTRLAYAGLKISEFGSLDYGRNYGVGYDIGSWTDMLPEFGGDTWSQKDVFMTYRTTGVATYRNYDFFGLIEGLNFAAQYQGKNERTDNGH-----**

**nmpC**  **SSKD-KTRLAFAGLKFGDYGSIDYGRNYGVAYDIGAWTDVLPEFGGDTWTQTDVFMTQRATGVATYRNNDFFGLVDGLNFAAQYQGKNDRSD--------**

**Ec042-1523**  **NQS--WTRLAFAGLKFADYGSFDYGRNYGVMYDIEGWTDMLPEFGGDSYTNADNFMTGRANGVATYRNTDFFGLVNGLNFAVQYQGNNEGASNGQ----E**

**ompN**  **NQS--WTRLAFAGLKFADYGSFDYGRNYGVMYDIEGWTDMLPEFGGDSYTNADNFMTGRANGVATYRNTDFFGLVNGLNFAVQYQGNNEGASNGQ----E**

**Ec042-2456**  **ENS--WTRVAFAGLKFQDVGSFDYGRNYGVVYDVTSWTDVLPEFGGDTYG-SDNFMQQRGNGFATYRNTDFFGLVDGLNFAVQYQGKN-GSVSGE----G**

**ompC**  **NNS--WTRVAFAGLKFQDVGSFDYGRNYGVVYDVTSWTDVLPEFGGDTYG-SDNFMQQRGNGFATYRNTDFFGLVDGLNFAVQYQGKN-GNPSGEGFTSG**

**Ec042-2121**  **QEK---TRLTFAGLKYNELGSFDYGRNYGVAYDAAAYTDMLVEWGGDSWASADNFMNGRTNGVATYRNYDFFGLVDGLDFAIQYQGKN------------**

**Clustal Consensus**  ****:::**** : **:***** *. ** .:**:: *:***: * *: * *.***** :***:::**::: ** *:***

210 220 230 240 250 260 270 280 290 300

....|....|....|....|....|....|....|....|....|....|....|....|....|....|....|....|....|....|....|....|

**Ec042-1020**  **--ERAGIPERSNGDGVGGSISYEF--EGFGIVGAYGAADRTDAQEAEFRGQ------GKKAEQWATGLKYDANNIYLAANYGETRNATPIEG--------**

**ompF**  **--ERD-TARRSNGDGVGGSISYEY--EGFGIVGAYGAADRTNLQEAQPLGN------GKKAEQWATGLKYDANNIYLAANYGETRNATPITNKF------**

**Ec042-0302**  **---ENRDVKKQNGDGFGTSLTYDFGGSDFAISGAYTNSDRTNEQNLQSRGT------GKRAEAWATGLKYDANNIYLATFYSETRKMTPIT---------**

**phoE**  **---ENRDVKKQNGDGFGTSLTYDFGGSDFAISGAYTNSDRTNEQNLQSRGT------GKRAEAWATGLKYDANNIYLATFYSETRKMTPIT---------**

**Ec042-1601**  **--LYGADYTRANGDGFGISSTYVY--DGFGIGAVYTKSDRTNAQERAAANP--LNASGKNAELWATGIKYDANNIYFAANYAETLNMTTYG---------**

**nmpC**  **----FDNYTEGNGDGFGFSATYEY--EGFGIGATYAKSDRTDTQVNAGKVLPEVFASGKNAEVWAAGLKYDANNIYLATTYSETQNMTVFA---------**

**Ec042-1523**  **GTNNGRDVRHENGDGWGLSTTYDLG-MGFSAGAAYTSSDRTNDQVNHTAAG------GDKADAWTAGLKYDANNIYLATMYSETRNMTPFGD--------**

**ompN**  **GTNNGRDVRHENGDGWGLSTTYDLG-MGFSAGAAYTSSDRTNDQVNHTAAG------GDKADAWTAGLKYDANNIYLATMYSETRNMTPFGD--------**

**Ec042-2456**  **MTNNGRDALRQNGDGVGGSITYDY--EGFGIGAAVSSSKRTDAQNTAAYIGN-----GDRAETYTGGLKYDANNIYLAAQYTQTYNATRVG---------**

**ompC**  **VTNNGRDALRQNGDGVGGSITYDY--EGFGIGGAISSSKRTDAQNTAAYIGN-----GDRAETYTGGLKYDANNIYLAAQYTQTYNATRVG---------**

**Ec042-2121**  **---SNRSTKKQNGDGYALSVDYNIN--GFGIVGAYSKSDRTNDQVADGNGS--------NAELWSLAAKYDANNVYAAVMYGETRNMTPGSIDTGVADRE**

**Clustal Consensus**  **. **** . * * .*. .. :.**: * .*: :: . ******:* *. * :* : ***

310 320 330 340 350 360 370 380 390 400

....|....|....|....|....|....|....|....|....|....|....|....|....|....|....|....|....|....|....|....|

**Ec042-1020**  **-------GFANKTQDVLLVAQYQFDFGLRPSIAYTKSKAKDVEG---------IGDVDLVNYFEVGATYYFNKNMSTYVDYIINQIDSDNKLG----VGS**

**ompF**  **---TNTSGFANKTQDVLLVAQYQFDFGLRPSIAYTKSKAKDVEG---------IGDVDLVNYFEVGATYYFNKNMSTYVDYIINQIDSDNKLG----VGS**

**Ec042-0302**  **------GGFANKTQNFEAVAQYQFDFGLRPSLGYVLSKGKDIEG---------IGDEDLVNYIDVGATYYFNKNMSAFVDYKINQLDSDNKLN----INN**

**phoE**  **------GGFANKTQNFEAVAQYQFDFGLRPSLGYVLSKGKDIEG---------IGDEDLVNYIDVGATYYFNKNMSAFVDYKINQLDSDNKLN----INN**

**Ec042-1601**  **-----DGYISNKAQSFEVVAQYQFDFGLRPSLAYLKSKGRDLG---------RYGDQDMIEYIDVGATYFFNKNMSTYVDYKINLID-ESDFTRAVDIRT**

**nmpC**  **-----DHFVANKAQNFEAVAQYQFDFGLRPSVAYLQSKGKDLG---------VWGDQDLVKYVDVGATYYFNKNMSTFVDYKINLLD-KNDFTKALGVST**

**Ec042-1523**  **----SDYAVANKTQNFEVTAQYQFDFGLRPAVSFLMSKGRDLHAAGGADNPAGVDDKDLVKYADVGATYYFNKNMSTYVDYKINLLDEDDSFYAANGIST**

**ompN**  **----SDYAVANKTQNFEVTAQYQFDFGLRPAVSFLMSKGRDLHAAGGADNPAGVDDKDLVKYADIGATYYFNKNMSTYVDYKINLLDEDDSFYAANGIST**

**Ec042-2456**  **-----SLGWANKAQNFEAVAQYQFDFGLRPSVAYLQSKGKNLGVI----NGRNYDDEDILKYVDVGATYYFNKNMSTYVDYKINLLD-DNRFTRDAGINT**

**ompC**  **-----SLGWANKAQNFEAVAQYQFDFGLRPSLAYLQSKGKNL--------GRGYDDEDILKYVDVGATYYFNKNMSTYVDYKINLLD-DNQFTRDAGINT**

**Ec042-2121**  **GNTIMRDQLINETQNFEAVVQYQFDFGLRPSLGYVYSKGKDIKGVPG----HRYVDADRVNYIEVGTWYYFNKNMNVYTAYKFNMLDKDDAAITG--AAA**

**Clustal Consensus**  ***::*.. ..**********::.: **.::: * * ::* ::*: *:*****..:. * :* :* ..**

410

....|....|..

**Ec042-1020**  **DDTVAVGIVYQF**

**ompF**  **DDTVAVGIVYQF**

**Ec042-0302**  **DDIVAVGMTYQF**

**phoE**  **DDIVAVGMTYQF**

**Ec042-1601**  **DNIVATGITYQF**

**nmpC**  **DDIVA---** ---

**Ec042-1523**  **DDIVALGLVYQF**

**ompN**  **DDIVALGLVYQF**

**Ec042-2456**  **DDIVALGLVYQF**

**ompC**  **DNIVALGLVYQF**

**Ec042-2121**  **DDQFAVGIVYQF**

**Clustal Consensus** ***: .* *:.*****
