# Supplementary material for: Complete Genome Sequence and Comparative Metabolic Profiling of the Prototypical Enteroaggregative Escherichia coli Strain 042
Source: PLoS One. 2010 Jan 20;5(1):e8801. doi: 10.1371/journal.pone.0008801 (PMC2808357; doi:10.1371/journal.pone.0008801)
Supplement: Figure S13 — Genetic architecture and distribution of the Chaperone-Usher systems of EAEC 042. (A) The organisation of the fimbrial loci from EAEC 042 are depicted. CDS flanking the fimbrial loci are depicted by green arrows, CDS encoding regulators are depicted by purple arrows, CDS encoding chaperones are indicated with black arrows, ushers are depicted with red arrows and the secreted fimbrial subunits by blue arrows. CDS of unknown function are depicted by orange arrows. The EAEC CDS designation for the first flanking gene is indicated along with the common name for the loci. The loci designated 0022 and 3336 are uncharacterised fimbrial loci and the numbers represent the EAEC CDS designation for the gene encoding the putative usher protein. (B) Phylogenetic distribution of the fimbrial loci amongst pathogenic and non-pathogenic lineages of E. coli. The loci encoding the EAEC fimbrial systems demonstrate a differential distribution amongst the E. coli phylogeny but with some evidence of phylogenetic clustering. (0.26 MB DOC) [file pone.0008801.s020.doc]

**A B**
