# Supplementary material for: Complete Genome Sequence and Comparative Metabolic Profiling of the Prototypical Enteroaggregative Escherichia coli Strain 042
Source: PLoS One. 2010 Jan 20;5(1):e8801. doi: 10.1371/journal.pone.0008801 (PMC2808357; doi:10.1371/journal.pone.0008801)
Supplement: File S1 — References for supplementary data. (0.04 MB DOC) [file pone.0008801.s027.doc]

1. Hayashi T, Makino K, Ohnishi M, Kurokawa K, Ishii K, et al. (2001) Complete genome sequence of enterohemorrhagic *Escherichia coli* O157:H7 and genomic comparison with a laboratory strain K-12. DNA Res 8: 11-22.

2. Iguchi A, Thomson NR, Ogura Y, Saunders D, Ooka T, et al. (2009) Complete genome sequence and comparative genome analysis of enteropathogenic *Escherichia coli* O127:H6 strain E2348/69. J Bacteriol 191: 347-354.

3. Casjens S (2003) Prophages and bacterial genomics: what have we learned so far? Mol Microbiol 49: 277-300.

4. Allison GE, Angeles D, Tran-Dinh N, Verma NK (2002) Complete genomic sequence of SfV, a serotype-converting temperate bacteriophage of *Shigella flexneri*. J Bacteriol 184: 1974-1987.

5. Pedulla ML, Ford ME, Karthikeyan T, Houtz JM, Hendrix RW, et al. (2003) Corrected sequence of the bacteriophage p22 genome. J Bacteriol 185: 1475-1477.

6. Deho G, Ghisotti D (2006) The Satellite Phage P4. In: Calendar R, editor. The Bacteriophages. 2nd ed. New York: Oxford University Press. pp. 391-408.

7. Dudley EG, Thomson NR, Parkhill J, Morin NP, Nataro JP (2006) Proteomic and microarray characterization of the AggR regulon identifies a *pheU* pathogenicity island in enteroaggregative *Escherichia coli*. Mol Microbiol 61: 1267-1282.

8. Touchon M, Hoede C, Tenaillon O, Barbe V, Baeriswyl S, et al. (2009) Organised genome dynamics in the *Escherichia coli* species results in highly diverse adaptive paths. PLoS Genet 5: e1000344.

9. Pukatzki S, McAuley SB, Miyata ST (2009) The type VI secretion system: translocation of effectors and effector-domains. Current Opinion in Microbiology 12: 11-17.

10. Deszo EL, Steenbergen SM, Freedberg DI, Vimr ER (2005) *Escherichia coli* K1 polysialic acid O-acetyltransferase gene, *neuO*, and the mechanism of capsule form variation involving a mobile contingency locus. Proc Natl Acad Sci U S A 102: 5564-5569.

11. Corbett D, Bennett HJ, Askar H, Green J, Roberts IS (2007) SlyA and H-NS regulate transcription of the *Escherichia coli* K5 capsule gene cluster, and expression of slyA in *Escherichia coli* is temperature-dependent, positively autoregulated, and independent of H-NS. J Biol Chem 282: 33326-33335.

12. Rowe S, Hodson N, Griffiths G, Roberts IS (2000) Regulation of the *Escherichia coli* K5 capsule gene cluster: evidence for the roles of H-NS, BipA, and integration host factor in regulation of group 2 capsule gene clusters in pathogenic E. coli. J Bacteriol 182: 2741-2745.

13. Stevens MP, Clarke BR, Roberts IS (1997) Regulation of the *Escherichia coli* K5 capsule gene cluster by transcription antitermination. Mol Microbiol 24: 1001-1012.

14. Heilmann C, Schweitzer O, Gerke C, Vanittanakom N, Mack D, et al. (1996) Molecular basis of intercellular adhesion in the biofilm-forming *Staphylococcus epidermidis*. Mol Microbiol 20: 1083-1091.

15. Kaniuk NA, Vinogradov E, Li J, Monteiro MA, Whitfield C (2004) Chromosomal and plasmid-encoded enzymes are required for assembly of the R3-type core oligosaccharide in the lipopolysaccharide of *Escherichia coli* O157:H7. J Biol Chem 279: 31237-31250.

16. Nakata N, Sasakawa C, Okada N, Tobe T, Fukuda I, et al. (1992) Identification and characterization of *virK*, a virulence-associated large plasmid gene essential for intercellular spreading of *Shigella flexneri.* Mol Microbiol 6: 2387-2395.

17. Detweiler CS, Monack DM, Brodsky IE, Mathew H, Falkow S (2003) virK, *somA* and *rcsC* are important for systemic *Salmonella enterica* serovar Typhimurium infection and cationic peptide resistance. Mol Microbiol 48: 385-400.

18. Kim SH, Jia W, Bishop RE, Gyles C (2004) An *msbB* homologue carried in plasmid pO157 encodes an acyltransferase involved in lipid A biosynthesis in *Escherichia coli* O157:H7. Infect Immun 72: 1174-1180.

19. Yoon JW, Lim JY, Park YH, Hovde CJ (2005) Involvement of the *Escherichia coli* O157:H7(pO157) ecf operon and lipid A myristoyl transferase activity in bacterial survival in the bovine gastrointestinal tract and bacterial persistence in farm water troughs. Infect Immun 73: 2367-2378.
