# Supplementary material for: Complete Genome Sequence and Comparative Metabolic Profiling of the Prototypical Enteroaggregative Escherichia coli Strain 042
Source: PLoS One. 2010 Jan 20;5(1):e8801. doi: 10.1371/journal.pone.0008801 (PMC2808357; doi:10.1371/journal.pone.0008801)
Supplement: Supplementary file 12 [file pone.0008801.s011.doc]

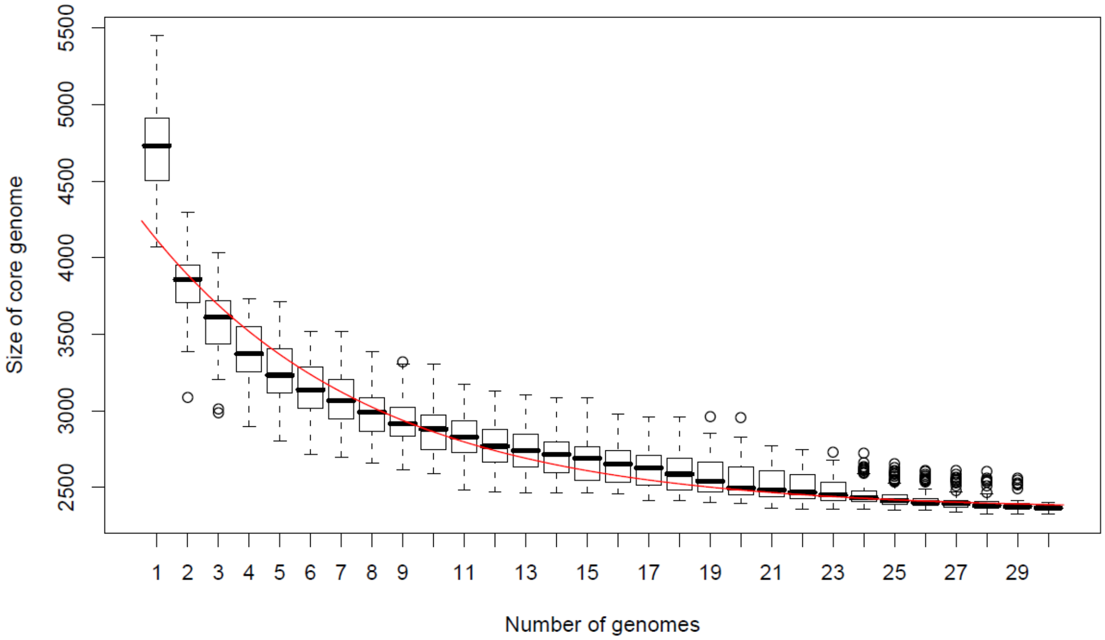
Size of core genome
2500
3000
3500
4000
4500
5000
5500
1
2
3
4
5
6
7
8
9
11
13
15
17
19
21
23
25
27
29
Number of genomes
