# Supplementary material for: Complete Genome Sequence and Comparative Metabolic Profiling of the Prototypical Enteroaggregative Escherichia coli Strain 042
Source: PLoS One. 2010 Jan 20;5(1):e8801. doi: 10.1371/journal.pone.0008801 (PMC2808357; doi:10.1371/journal.pone.0008801)
Supplement: Supplementary file 13 [file pone.0008801.s013.doc]

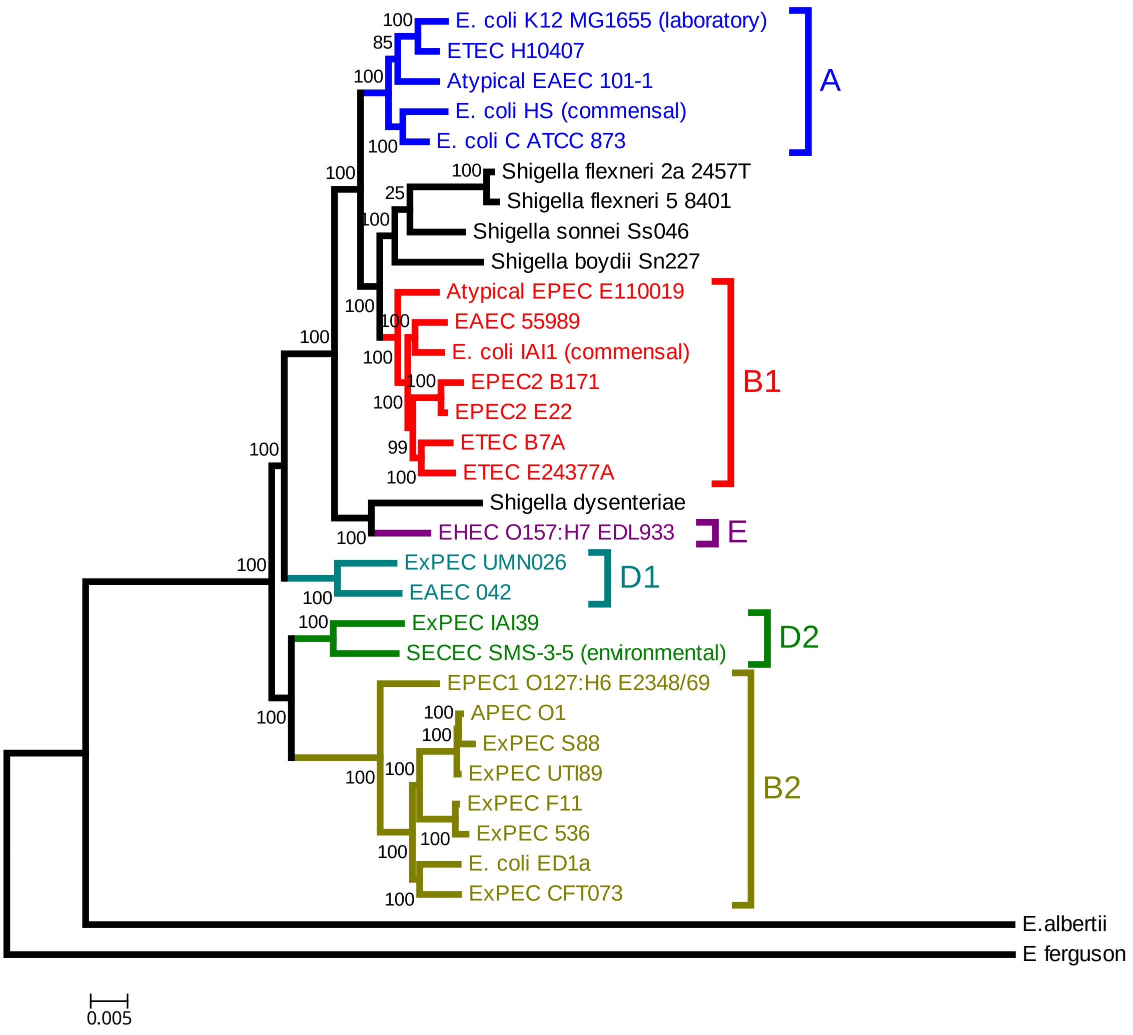

E. coli K12 MG1655 (laboratory)
ETEC H10407
Atypical EAEC 101-1
E. coli HS (commensal)
E. coli C ATCC 873
A
Shigella flexneri 2a 2457T
Shigella flexneri 5 8401
Shigella sonnei Ss046
Shigella boydii Sn227
Atypical EPEC E110019
EAEC 55989
E. coli IAI1 (commensal)
EPEC2 B171
EPEC2 E22
ETEC B7A
ETEC E24377A
B1
Shigella dysenteriae
EHEC O157:H7 EDL933
E
ExPEC UMN026
EAEC 042
D1
ExPEC IAI39
SECEC SMS-3-5 (environmental)
D2
EPEC1 O127:H6 E2348/69
APEC O1
ExPEC S88
ExPEC UTI89
ExPEC F11
ExPEC 536
E. coli ED1a
ExPEC CFT073
B2
E.albertii
E ferguson
0.005

1
2
